# Synthesis of Cyclic and Acyclic *ortho*‐Aryloxy Diaryliodonium Salts for Chemoselective Functionalizations

**DOI:** 10.1002/chem.202202453

**Published:** 2022-10-19

**Authors:** Erika Linde, Niels Knippenberg, Berit Olofsson

**Affiliations:** ^1^ Department of Organic Chemistry Arrhenius Laboratory Stockholm University SE-106 91 Stockholm Sweden

**Keywords:** atom-efficient transformations, diaryl ethers, diaryliodonium salts, hypervalent compounds, transition metal-free

## Abstract

Two regioselective, high‐yielding one‐pot routes to oxygen‐bridged cyclic diaryliodonium salts and *ortho*‐aryloxy‐substituted acyclic diaryliodonium salts are presented. Starting from easily available *ortho*‐iodo diaryl ethers, complete selectivity in formation of either the cyclic or acyclic product could be achieved by varying the reaction conditions. The complimentary reactivities of these novel ortho‐oxygenated iodonium salts were demonstrated through a series of chemoselective arylations under metal‐catalyzed and metal‐free conditions, to deliver a range of novel, *ortho*‐functionalized diaryl ether derivatives.

## Graphical abstract

*Ortho*‐iodo diaryl ethers are selectively transformed into either oxygen‐bridged cyclic diaryliodonium salts or *ortho*‐aryloxy‐substituted acyclic diaryliodonium salts through regioselective and high‐yielding one‐pot routes. A series of chemoselective arylations under metal‐catalyzed and metal‐free conditions shows the complimentary reactivities of these novel electrophilic arylation reagents and deliver a range of novel, *ortho*‐functionalized diaryl ether derivatives.

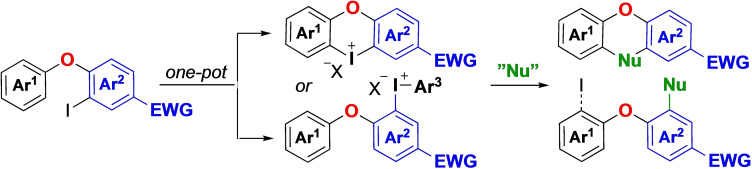

## Introduction

**Scheme 1:**
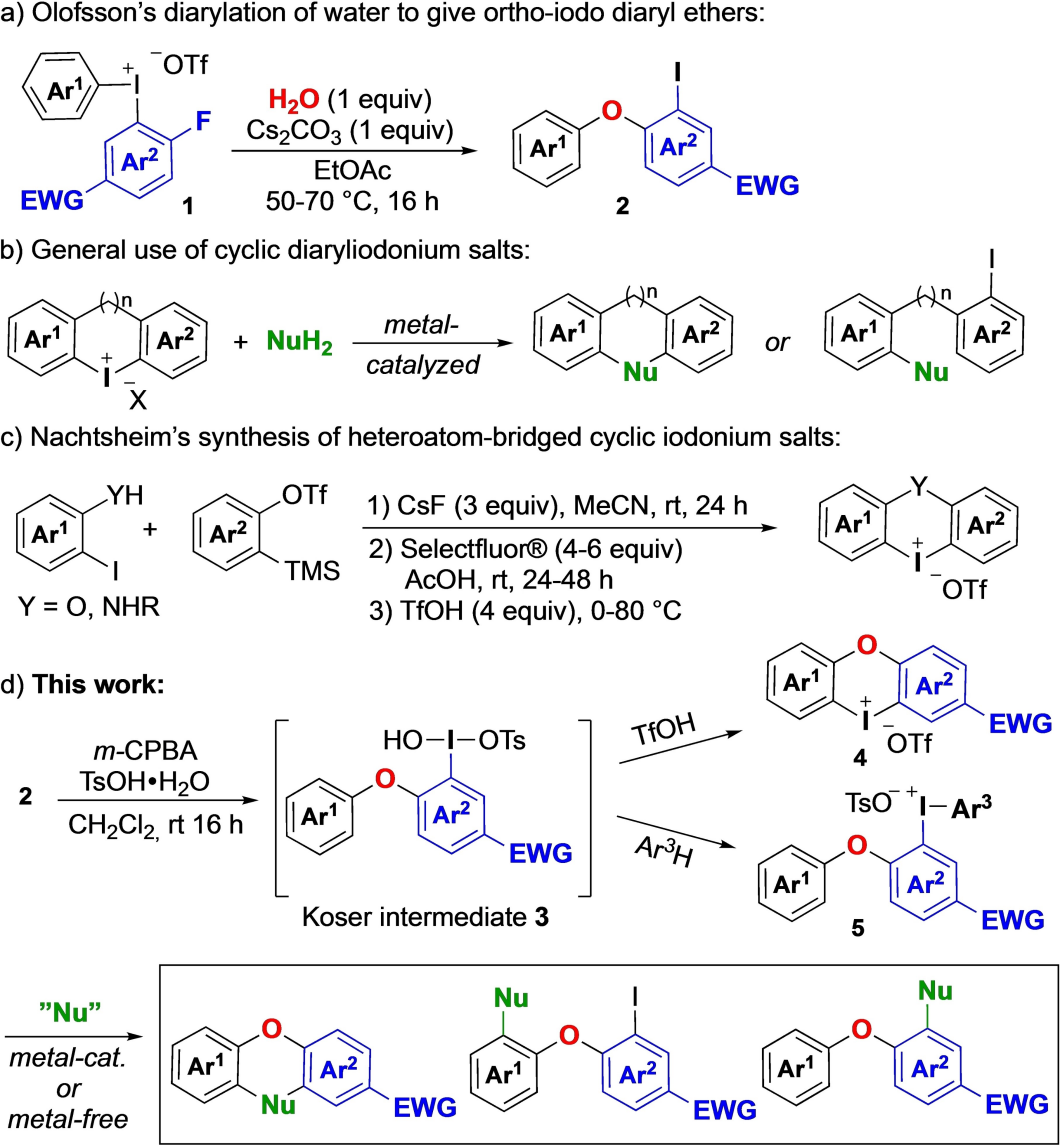
Synthesis and applications of cyclic and acyclic Ar_2_IX.

Diaryl ethers are important building blocks for pharmaceuticals, agrochemicals and natural products,^[1]^ and efficient synthetic routes to such motifs are of high interest. Functionalization methods of diaryl ethers would further simplify the access to a wide variety of products of potential biological interest.^[1]^ The most common strategies for diaryl ether preparation includes metal‐catalyzed cross couplings,^[2]^ nucleophilic aromatic substitution^[3]^ and oxidative couplings with phenols.^[4]^ We recently reported an efficient diarylation of nitrogen and oxygen nucleophiles, whereby both aryl groups of diaryliodonium salts **1** were transferred to the nucleophile under mild conditions.^[5]^ *Ortho*‐iodo‐substituted diaryl ethers **2** decorated with electron‐withdrawing groups (EWG) were formed with water as the nucleophile (Scheme 1a).^[5]^ To increase the functionalization possibilities of diaryl ethers **2** beyond iodoarene cross couplings, we envisioned that the iodine handle could be reoxidized to iodine(III) and form a diaryliodonium salt.

Diaryliodonium salts are well‐known electrophilic arylating reagents that can be employed under both metal‐free and metal‐catalyzed conditions.^[6]^ While they generally give rise to stoichiometric ArI waste formation, a range of atom‐efficient applications have recently been developed.[^5^, ^7^] In that vein, cyclic diaryliodonium salts have received considerable attention as both aryl groups are transferred to the nucleophile (Scheme 1b).[^7c^, ^8^] Application areas of cyclic diaryliodonium salts include the preparation of various heterocyclic compounds, ligands and large conjugated systems.^[9]^ Furthermore, they have been used as halogen‐bonding organocatalysts,^[10]^ and dibenziodolium chloride (diphenyleneiodonium, DPI) is commonly used in biological studies.^[11]^ The structures of several types of cyclic iodonium salts have also been investigated.[^8d^, ^12^]

Efficient one‐pot routes from iodoarenes and arenes under acidic, oxidative conditions give facile access to symmetric and unsymmetric acyclic diaryliodonium salts.^[13]^ Cyclic iodonium salts with a carbon backbone can be synthesized under similar conditions, and recent progress in the field includes the discovery of heterocyclic diaryliodonium salts,^[14]^ as well as heteroatom‐bridged cyclic salts.[^10b^, ^15^] The latter compound class was until very recently only reported in modest yields without scope evaluations or reactivity studies.[^10b^, ^15^]

In 2022, Nachtsheim and coworkers presented an elegant route to oxygen‐bridged cyclic diaryliodonium triflates by treating *ortho*‐iodo phenols with arynes followed by in situ oxidation with Selectfluor (Scheme 1c).^[16]^ A large variety of phenols could be used, whereas the reaction was limited to only two aryne precursors due to regioselectivity or decomposition problems. The products were obtained in moderate to good yields, and were demonstrated as useful coupling partners mainly in transition metal‐catalyzed transformations.^[16]^

In this work, we present the high‐yielding oxidation of *ortho*‐iodo diaryl ethers **2** with inexpensive *m*‐CPBA to Koser intermediates **3**, which can either be transformed to cyclic oxygen‐bridged diaryliodonium salts **4** or reacted with a suitable arene to form acyclic iodonium salts **5** with complete regioselectivity (Scheme 1d). Reactivity investigations of iodonium reagents **4** & **5** in chemoselective arylations to yield functionalized diaryl ethers are also presented. The work is complimentary to Nachtsheim's study,^[16]^ which was reported during our investigations, and goes beyond that as both transition metal‐catalyzed and metal‐free conditions are efficient in the applications of the iodonium salts and the chemoselectivity of the reactions is studied.

## Results and Discussion

We started exploring the one‐pot synthesis of cyclic iodonium salts **4** using *m*‐CPBA under strongly acidic conditions, according to our previously developed methodology for one‐pot synthesis of acyclic diaryliodonium salts.[^13b^, ^13c^, ^13d^] The reagent combination *m*‐CPBA/TfOH has also proven efficient in the synthesis of cyclic iodonium salts.[^8c^, ^9^, ^10b^, ^14c^, ^17^] When *ortho*‐iodo diaryl ether **2 a** was treated with *m*‐CPBA and triflic acid the reaction did indeed yield the desired product **4**, however in an inseparable mixture with an unidentified byproduct (Table 1, entry 1). While BF_3_ ⋅ OEt_2_ was a poor reactant for this transformation (entry 2), the use of tosic acid in CH_2_Cl_2_ led to successful oxidation of **2 a**, yet no cyclization occurred under these conditions^[18]^ and the corresponding Koser's derivative **3 a** (X=OTs) was obtained in 93 % isolated yield (entry 3). With these results, we envisioned that addition of the stronger acid TfOH upon the in situ formation of **3 a** would allow the cyclization to take place faster. Delightfully, this strategy was realized and when lowering the loading of TsOH from 3.5 to 1.5 equiv and performing the reaction in CH_2_Cl_2_ : TFE,^[13d]^ the target product was isolated in excellent yield (entry 4).

**Table 1:** Optimization of the synthesis of cyclic salt **4**.

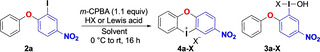					
Entry	Acid (equiv)	Solvent	Additive	Yield of **4 a** (%)^[a]^	Yield of **3 a** (%)^[a]^
1	TfOH (2.0)	CH_2_Cl_2_	–	Impure^[b]^ (X=OTf)	0^[b]^
2	BF_3_ ⋅ OEt_2_ (2.5)	CH_2_Cl_2_	–	traces^[b]^ (X=BF_4_)	0^[b]^
3	TsOH ⋅ H_2_O (3.5)	CH_2_Cl_2_	–	0	93 (X=OTs)
4	TsOH ⋅ H_2_O (1.5)	CH_2_Cl_2_ : TFE 1 : 1	TfOH^[c]^	98 (X=OTf)	0

[a] Isolated yields. [b] An unidentified byproduct was isolated with **4 a**. [c] TfOH added after 16 h at 0 °C, the reaction was stirred for 6 additional hours at rt.

**Scheme 2:**
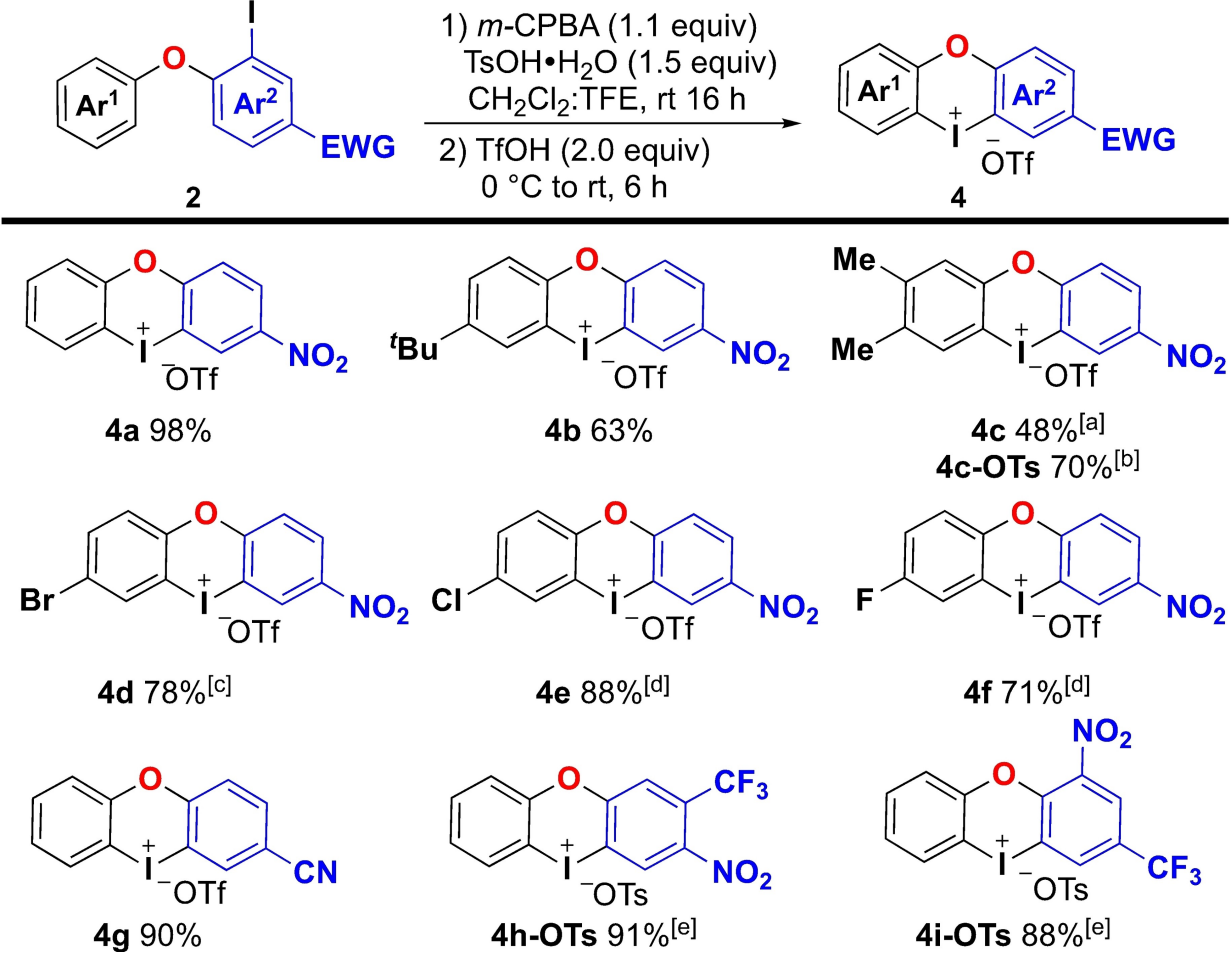
Substrate scope of cyclic salts **4**. [a] 3.0 equiv. of TfOH used. [b] Reaction conditions as in Scheme 4. [c] Step 2 reaction time 24 h. [d] Step 2 at 50 °C for 24 h. [e] Reaction performed without addition of TfOH at rt for 30 h.

A small substrate scope was conducted under the optimized conditions, utilizing the diaryl ethers **2** that were available from iodonium salts **1** (Scheme 2). Substituents in the Ar^1^ group of ether **2** were first evaluated, and electron‐donating alkyl groups were well tolerated giving iodonium salts **4 b** and **4 c**. The corresponding tosylate salt **4 c**‐**OTs** could later be obtained in 70 % yield through slightly different conditions (see below). Substrates with electron‐withdrawing substituents in Ar^1^ were less reactive under the standard conditions, likely due to a reduced rate in the electrophilic aromatic substitution step. The yields could be increased significantly by increasing the reaction time to 24 h and in some instances also the temperature to 50 °C during the cyclization step,^[19]^ which afforded product **4 d**–**f4** in good yields.

Variations in the Ar^2^‐group of diaryl ethers **2** were also feasible, as demonstrated with products **4 g**–**i**. Interestingly, when a trifluoromethyl substituent was added to the Ar^2^ group, triflic acid was no longer required to achieve the cyclization of intermediate **3**, and tosylate salts **4 h** and **4 i** were obtained in 91 % and 88 % yield, respectively. The strong electron‐withdrawing nature of the Ar^2^ group in those substrates likely results in an increased electrophilic aromatic substitution (EAS) cyclization rate.

Our scope investigation was limited to the substrates **2** that could be obtained from iodonium salts **1**, and further substituents might be tolerated through alternative routes to **2**. In comparison with previous methodology for synthesis of oxygen‐bridged iodonium salts,^[16]^ our method avoids excess reagents and gives higher yields.^[20]^

**Scheme 3:**
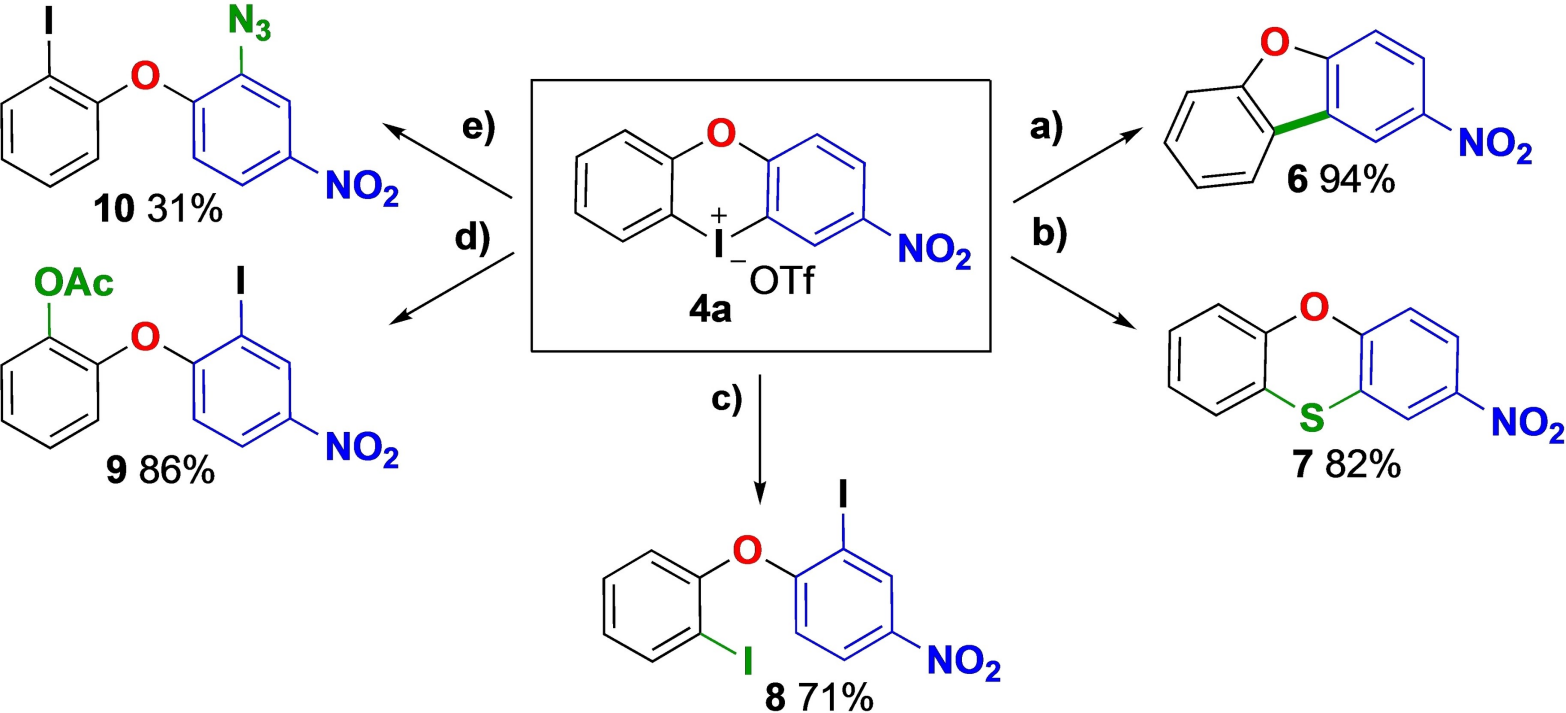
Arylations with cyclic salt **4 a**. a) NaOAc (3.0 equiv), Pd/C (5 mol %), DMA, 140 °C, 16 h. b) Triethylammonium benzylcarbamodithioate (2.0 equiv), CuSO_4_ (20 mol %), bpy (40 mol %), MeCN, rt to 90 °C, 20 h. c) Bu_4_NI (2.0 equiv), CuI (20 mol %) DMEDA (20 mol %), dioxane, 50 °C, 24 h. d) NaOAc (3.0 equiv) CuSO_4_ ⋅ 5H_2_O (5 mol %), AcOH/Ac_2_O, 120 °C, 16 h; e) NaN_3_ (1.2 equiv), DMA, 120 °C, 1 h. DMA=*N,N*‐dimethylacetamide, bpy=2,2’‐bipyridyl, DMEDA=*N,N’*‐dimethylethylenediamine.

Nachtsheim and co‐workers demonstrated the reactivity of their unsubstituted model product, diphenyliodaoxinium triflate, in a range of literature‐based applications.^[16]^ Inspired by this work, we explored the reactivity of our electron‐deficient, unsymmetrical product **4 a** in a series of atom‐efficient transformations following literature precedent (Scheme 3). The annulation^[21]^ of **4 a** to the corresponding benzophenone **6** was achieved in 94 % yield. Double S‐arylation was also efficient, as demonstrated by the Cu‐catalyzed sulfur‐iodine exchange,^[15b]^ yielding the phenoxathiine derivative **7**. Mono‐functionalizations giving ring‐opened products were also productive. In this way, a Cu‐catalyzed cross‐coupling with Bu_4_NI^[9b]^ delivered the diiodinated compound **8** in 71 % yield. The Cu‐catalyzed acetoxylation^[22]^ of **4 a** occurred with complete chemoselectivity for functionalization of the phenyl group, giving product **9** in 86 % yield. Unfortunately, the Cu‐catalyzed amination with 4‐chloroaniline proceeded with moderate chemoselectivity to deliver a 2 : 1 product mixture.^[8c]^ In comparison to the unsubstituted diphenyliodaoxinium triflate,^[16]^ nitro‐substituted salt **4 a** gave similar yields in the transformations described above. To the contrary, no reactivity was observed in a metal‐free bromination through thermolysis,^[23]^ illustrating the influence of the electron‐withdrawing nitro group.

The low reactivity of cyclic diaryliodonium salts is well documented, and transition metal catalysis is required for the vast majority of their transformations.[^8a^, ^8b^] Thus, we were pleased that the transition metal‐free azidation^[24]^ of **4 a** gave product **10** with complete chemoselectivity towards functionalization of the most electron‐deficient aryl group. Notably, metal‐free arylations provide an opportunity to transfer the iodine atom from Ar^2^ to Ar^1^ in regards to the diaryl ether **2**, due to the reversed chemoselectivity compared to the metal‐catalyzed arylations.

Due to the limited reactivity of cyclic salts **4** under transition metal‐free conditions, we envisioned that arylations with the corresponding acyclic salts **5** would be of high utility as valuable building blocks for complex diaryl ethers. Chemoselective reactions with unsymmetric diaryliodonium salts require a suitable “dummy” aryl group that is transferred to the nucleophile, e.g an anisyl, trimethoxyphenyl or a mesityl group.^[25]^

Koser's derivative **3 a** (Table 1, entry 3) could indeed be transformed to iodonium salt **5 a** by treatment with anisole in TFE.[^19^, ^26^] We then investigated the one‐pot synthesis^[13d]^ of product **5 a** from **2 a** with *m*‐CPBA and TsOH (Table 2).^[19]^ When the reaction was performed in CH_2_Cl_2_ : TFE, cyclic salt **4 a**‐**OTs** was the only product (entry 1). By excluding TFE in the oxidation step, undesired cyclization of the in situ formed **3 a** was avoided, and the desired product **5 a** was obtained in excellent yield upon addition of anisole and TFE (entry 2). TFE has proven to be an efficient solvent for ligand exchange reactions with Koser's reagents,[^26^, ^27^] and excluding this solvent in the second step indeed reduced the yield slightly (entry 3). Exclusion of TFE also allowed a one pot‐one step reaction setup with the anisole present from the start of the reaction, giving the product **5 a** in 86 % yield within 16 h (entry 4).

**Table 2:** Optimization of the synthesis of acyclic salt **5 a**.

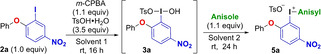				
Entry	Solvent 1	Solvent 2	Yield of **5 a** (%)^[a]^	Yield of **4 a**‐**OTs** (%)^[a]^
1	CH_2_Cl_2_ : TFE 1 : 1	–	0	85
2	CH_2_Cl_2_	TFE	95	0
3	CH_2_Cl_2_	–	86	0
4	CH_2_Cl_2_	–	86^[b]^	0

[a] Isolated yields. [b] Reaction performed in one pot for 16 h, with anisole present from the start of the reaction.

**Scheme 4:**
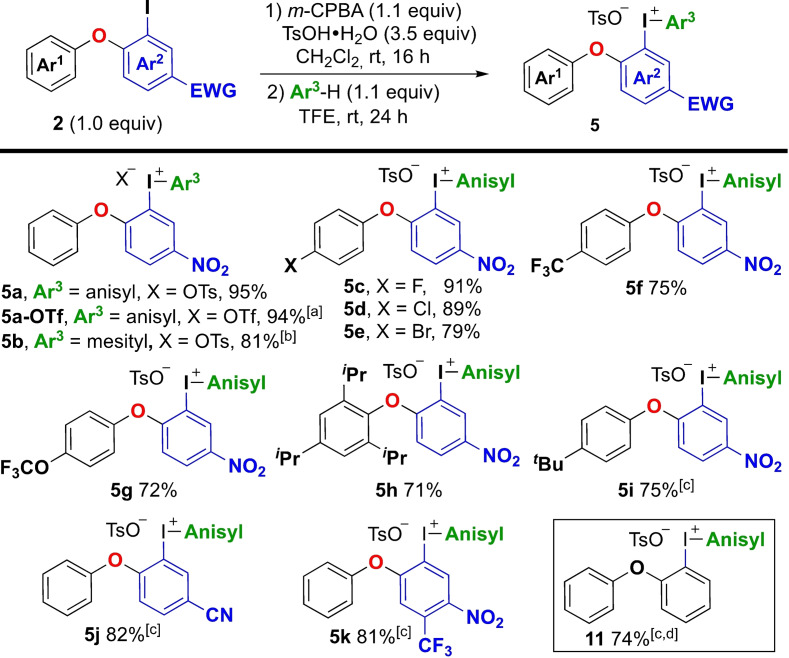
Substrate scope of salt **5**. [a] Upon completion of the reaction, TfOH (2.0 equiv) was added and the reaction was stirred 30 min at rt. [b] By reaction of mesitylene (1.1 equiv) with isolated **3 a**. [c] Anisole (1.1–3.0 equiv) present from the start of the reaction in the absence of TFE, reaction time 16 h. [d] Synthesized from (2‐iodophenyl)phenyl ether.

The scope of the methodology was evaluated under the optimized conditions (Scheme 4). The counter ion (X) of Ar_2_IX reagents has a great impact on their reactivity, and triflate salts usually perform better than tosylate salts.^[6b]^

An in situ anion exchange by addition of TfOH upon completion of the reaction was hence performed,^[13d]^ and delivered **5 a**‐**OTf** in 94 % yield. Since the sterically demanding mesityl group is an efficient dummy ligand in transition metal‐catalyzed cross couplings with Ar_2_IX reagents,^[6c]^ the corresponding mesityl salt **5 b** was also of interest. Under the optimized conditions, **5 b** was obtained as an inseparable mixture with the corresponding cyclic salt **4 a**‐**OTs**. Fortunately, the reaction of mesitylene with the isolated Koser intermediate **3 a** resolved this issue and provided compound **5 b** in 81 % yield.

Next, we evaluated the substrate scope of diaryl ethers **2**. Electron deficient Ar^1^‐groups were well tolerated, giving products **5 c**–**5 g** in high yields. Furthermore, electron rich and highly sterically strained TRIP‐product **5 h** was obtained in 71 % yield.

Products **5** were sometimes formed as mixtures with the competing cyclized product **4**‐**OTs**, and dimethylphenoxy substrate **2 c** selectively delivered the cyclic salt **4 c**‐**OTs** (see Scheme 2).^[19]^ In such cases, selective formation of **5** was achieved by performing the reaction as a one‐step reaction without TFE, with anisole present from the start of the reaction (see Table 2, entry 4). Under these conditions, compound **5 i** was obtained with complete selectivity in 75 % yield. Variations on Ar^2^ were also feasible, allowing alternative or additional electron withdrawing groups, as shown by the synthesis of products **5 j**–**k**. Furthermore, the methodology could be extended to *ortho*‐iodo diaryl ethers lacking electron‐withdrawing groups (i. e. not prepared from diaryliodonium salts **1**), as demonstrated by the synthesis of product **11** in 74 % yield.

**Scheme 5:**
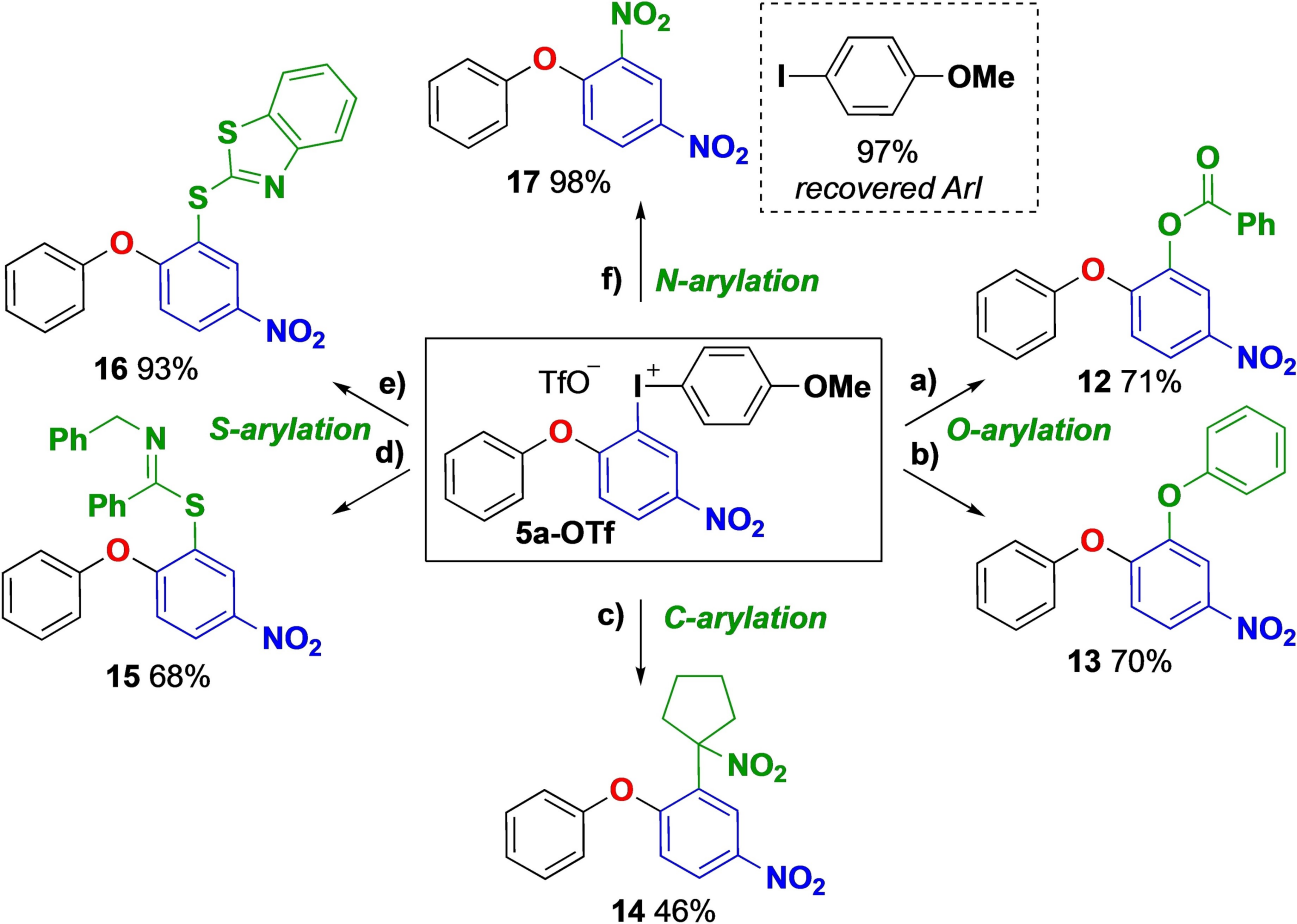
Transition metal‐free arylations with **5 a**‐**OTf**. a) Benzoic acid (1.0 equiv), ^*t*^BuOK (1.2 equiv), toluene, 130 °C, 1 h. b) Phenol (1.0 equiv), ^*t*^BuOK (1.2 equiv), THF, 40 °C, 3 h. c) Nitrocyclopentane (1.0 equiv), ^*t*^BuOK (1.2 equiv), DME, rt, 16 h. d) *N*‐Benzyl‐thiobenzamide (1.0 equiv), ^*t*^BuOLi (1.1 equiv), toluene, 80 °C, 2 h. e) 2‐Benzylmercaptothiol (1.0 equiv), DBU (1.1 equiv), MeCN, 80 °C, 3 h. f) NaNO_2_ (1.1 equiv), EtOAc, 70 °C, 16 h. DME=1,2‐dimethoxyethane, DBU=1,8‐diazabicyclo[5.4.0]undec‐7‐ene.

To demonstrate the utility of these complex diaryliodonium salts, **5 a**‐**OTf** was used in a series of synthetic transformations under transition metal‐free conditions (Scheme 5).^[28]^ O‐arylations of benzoic acid^[28a]^ and phenol^[28b]^ with **5 a**‐**OTf** were achieved with complete chemoselectivity for functionalization of the most electron‐deficient aryl group, giving **12** and **13** in good yields. Less reactive carbon nucleophiles such as nitro‐cyclopentane were productive in the late‐stage functionalization of the ether scaffold, giving product **14**.^[28c]^ S‐arylation[^28d^, ^28e^] was efficient with both thioamides and 2‐mercabenzothiazole, affording the corresponding products **15** and **16** in 68 % and 93 % yield, respectively. Also the nitration^[28f]^ of **5 a**‐**OTf** proceeded in excellent yield, and the iodoanisole byproduct was recovered in 97 %, making it accessible for further transformations. This is important to decrease the drawback of lower atom economy in reactions with acyclic Ar_2_IX compared to their cyclic analogues.

Notably, all products derived from salts **5 a**‐**OTf** and **4 a**, with the exception of **6** and **17**, are new compounds with unexplored and potentially interesting properties. The corresponding functionalizations of diaryl ethers **2** would be difficult to perform under transition metal‐free conditions, for example through a nucleophilic aromatic substitution pathway.

## Conclusion

We have reported synthetic methodology for one‐pot preparations of oxygen‐bridged cyclic diaryliodonium salts **4**, as well as highly functionalized acyclic diaryliodonium salts **5**, starting from *ortho*‐iodinated diaryl ethers **2**. The synthesis proceeds with complete regioselectivity, and is thus complimentary to previous synthetic methods. Both methodologies are compatible with electron rich and electron poor ether substrates, and highly sterically demanding ethers were tolerated in the synthesis of the acyclic salts. The reactivity of these novel reagents was demonstrated in a series of arylations, giving access to a range of functionalized products. The oxygen‐bridged cyclic salts proved most productive under transition metal‐catalyzed conditions, and were employed in various atom‐efficient transformations giving both mono‐ and di‐functionalized products. The acyclic ether‐functionalized iodonium salts displayed higher reactivity compared to their cyclic analogues and could be used in arylations where the anisole dummy ligand proved to be an efficient choice to achieve chemoselective coupling of the ether moiety with various nucleophiles. In this way, a versatile range of transition metal‐free transformations were achieved to provide novel, functionalized diaryl ether products in good to excellent yields.

## Experimental Section

**Synthesis of cyclic diaryliodonium salts 4**: Diaryl ether **2** (1.0 equiv), *m*‐CPBA (1.1 equiv), and TsOH ⋅ H_2_O (1.5 equiv) were added to a round‐bottomed flask, followed by CH_2_Cl_2_ : TFE (1 : 1 (v/v) 0.2 m). The reaction was stirred at room temperature for 16 h before being placed in an ice bath followed by drop wise addition of TfOH (2.0 equiv). When the reaction had stopped fuming, it was stirred for 6 additional hours at room temperature. Afterwards, the solvent was removed *in vacuo* and the product was triturated in Et_2_O. Product **4** was isolated by filtration, washed with Et_2_O and dried under vacuum.

**Synthesis of acyclic diaryliodonium salts 5**: Diaryl ether **2** (1.0 equiv), *m*‐CPBA (1.1 equiv), and TsOH ⋅ H_2_O (3.5 equiv) were added to a round‐bottomed flask followed by CH_2_Cl_2_ (0.2 m). The reaction mixture was stirred at room temperature for 16 h. TFE (0.2 m) was added, followed by drop wise addition of anisole (1.1 equiv). The reaction was stirred for additional 24 h at room temperature. Afterwards, the solvent was removed *in vacuo* and the product was triturated in Et_2_O. Product **5** was isolated by filtration, washed with Et_2_O and dried under vacuum

## Conflict of interest

The authors declare no conflict of interest.

1

## Supporting information

As a service to our authors and readers, this journal provides supporting information supplied by the authors. Such materials are peer reviewed and may be re‐organized for online delivery, but are not copy‐edited or typeset. Technical support issues arising from supporting information (other than missing files) should be addressed to the authors.

Supporting Information

## Data Availability

The data that support the findings of this study are available in the supplementary material of this article.
