# Supplementary material for: Synthesis of Cyclic and Acyclic ortho‐Aryloxy Diaryliodonium Salts for Chemoselective Functionalizations
Source: Chemistry. 2022 Oct 19;28(69):e202202453. doi: 10.1002/chem.202202453 (PMC10092902; doi:10.1002/chem.202202453)
Supplement: Supplementary file 1 — Supporting Information [file CHEM-28-0-s001.pdf]

Chemistry–A European Journal

Supporting Information

Synthesis of Cyclic and Acyclic *ortho*-Aryloxy Diaryliodonium Salts for Chemoselective Functionalizations

Erika Linde, Niels Knippenberg, and Berit Olofsson*

Table of content

Methods and materials 2

1. Optimization studies 3

1.1 Optimization of the synthesis of cyclic salts 4 3

1.1.2 Reactions with electron poor and electron rich substrates 3

1.2 Optimization of the synthesis of acyclic salts 5 5

1.2.1 Reactions with mesitylene as dummy ligand 6

2. Limitations in applications of cyclic salt 4a 7

3. Synthesis of diaryliodonium salts 1 8

4. Synthesis of diaryl ethers 2 (GP3) 11

4.1 Alternative method to prepare diaryl ethers 12

5. Synthesis of cyclic diaryliodonium salts 4 13

5.1 General procedure 4 (GP4) 13

5.2 Synthetic details and analytical data of 4 13

6. Synthesis of acyclic diaryliodonium salts 5 16

6.1 General procedure 5 (GP5): 16

6.2 General procedure 6 (GP6): 16

6.3 Synthetic details and analytical data of 5 16

6.4 Stepwise synthesis of mesityl salt 5b 19

7. Derivatization of products 4a and 5a-OTf 21

References: 25

NMR spectra 25

Methods and materials

The reagents were bought from commercial suppliers and used as received, unless noted otherwise. Moisture and air sensitive reactions were carried out under argon environment using standard Schlenk techniques. Reactions performed above the boiling point of the solvent(s) were performed in pressure-stable microwave vials. Solvents were obtained as P.A. grade. The EtOAc used for the synthesis of the diaryl ethers was stored over molecular sieves (4 Å) and degassed by bubbling argon through it with a needle before use. *m*-CPBA (Aldrich, 77 % active oxidant) was dried under vacuum, after which the amount of active oxidant was determined through an iodometric titration.[1] Thin layer chromatography (TLC) was performed using TLC Silica gel 60 F^{254} plates (Merck) and visualized using UV-light. Purification of the products was conducted by flash column chromatography on SiO_2 purchased from Aldrich (technical grade, 60 Å pore size, 230-400 mes). Melting points were measured using a STUART SMP3 and are reported uncorrected. NMR measurements were carried out using a 400 MHz Bruker AVANCE II with a BBO probe at 298 K. Chemical shifts are given in ppm relative to the residual solvent peak (^{1}H NMR δ: $CDCl_3$ = 7.26; DMSO-d_6 = 2.50; CD_3OD = 3.35, 4.78. ^{13}C NMR δ: $CDCl_3$ = 77.16; DMSO-d_6 = 39.52; CD_3OD = 49.3, 39.52) [2] Shift values are reported in ppm and all coupling constants (J) are printed in Hertz (Hz) with their multiplicity: s (singlet), br (broad signal), d (doublet), t (triplet), q (quartet), pent (pentet), m (multiplet). HRMS spectra were measured on a Bruker microTOF with electron spray ionization (ESI).

1. Optimization studies

1.1 Optimization of the synthesis of cyclic salts 4

The optimization of reaction conditions for the synthesis of diaryliodonium salt **4** from *ortho*-iodo diaryl ether **2a** is summarized in Table S1. With *m*-CPBA as the oxidant in combination with TfOH the reaction did indeed yield the desired product **4**, however in an inseparable mixture with an unidentified side-product (entries 1-3). Hence the yields could not be determined. Extending the reaction time did not have an effect on the yield (entry 2), nor adding an excess of H_2SO_4 (entry 3), in an attempt to facilitate the cyclization step. The reaction with the Lewis acid $BF_3{\cdot}OEt_2$ provided product **4** in only traces, also in a mixture with side-products (entry 4). Use of TsOH in CH_2Cl_2 led to successful oxidation of the iodine, but no intramolecular cyclization (entry 5). Instead the reaction stopped after the oxidation, giving the corresponding Koser's derivative **3a** (X = OTs) in 93% isolated yield. With these results, we anticipated that if the Koser's derivative could be formed *in situ*, adding a stronger acid such as TfOH would allow the cyclization to take place. Delightfully, this did indeed yield the desired product **4a** in an excellent yield of 98% (entry 6). By performing the reaction in CH_2Cl_2:TFE (1:1) we could lower the loading of TsOH from 3.5 to 1.5 equiv.

Table S1. Optimization of conditions for synthesis of salt **4a**.

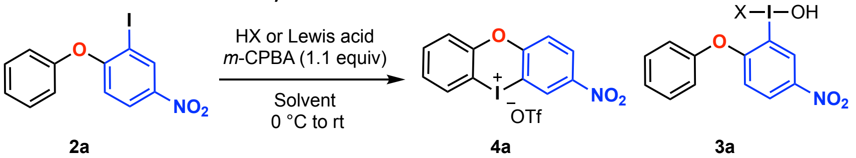

Entry	Acid (equiv)	Solvent	Time (h)	Additive (equiv)	Yield of 4a (%)	Yield of 3a (%)
**1**	TfOH (2.0)	CH_2Cl_2	3		impure[a]	n.d
**2**	TfOH (2.0)	CH_2Cl_2	16		impure[a]	n.d
**3**	TfOH (2.0)	CH_2Cl_2	16	H_2SO_4 (5.0 equiv), 0 °C to rt, 6 h[a]	impure[a]	n.d
**4**	$BF_3{\cdot}OEt_2$ (2.5)	CH_2Cl_2	16		traces	n.d
**5**	TsOH (3.5)	CH_2Cl_2	16		0	93 X= OTs
**6**	TsOH (1.5)	CH_2Cl_2:TFE (1:1)	16	TfOH (2.0 equiv), 0 °C to rt, 6 h[b]	98 X = OTf	0

All isolated yields. n.d = not detected. [a] An unidentified byproduct was isolated with **4a**. [b] The additive was added after 16 h at 0 °C, the reaction was stirred for 6 additional hours at rt.

1.1.2 Reactions with electron poor and electron rich substrates

When performing the reaction with the brominated diaryl ether **2d**, the product **4d** was obtained in a poor yield of 19% (Table S2, entry 1). We speculated that the low yield was due to a slower cyclization step as a result of the electron withdrawing bromine substituent. A few conditions were screened to improve the yield, and by performing the cyclization step at 24 h, the yield could be significantly increased to 79% (entry 2). Keeping the reaction time but increasing the temperature to 50 °C had a similar positive effect, giving the product **4d** in 65% yield (entry 3). Surprisingly, increasing the temperature further to 70 °C resulted in the formation of the cyclic salt **4d-OTs** as the only product (entry 4). We speculate that the elevated temperature results in a fast cyclization of the Koser intermediate before the ion exchange with OTf has taken place. In an attempt to increase the efficiency of the latter counter ion exchange, the solvent for the cyclization step was changed to MeCN (entry 5). However, this also provided **4d-OTs** as the only product. Thus, we established that the best conditions

for preparation of **4d** was to perform the cyclization at room temperature for 24 h (entry 2). Nevertheless, the reaction of the chlorine and fluorine analogues **2e** and **2f** under these conditions yielded the corresponding products in only 52% and 30% yield, respectively (entries 6-7). Delightfully, these yields could be increased significantly by performing the cyclization step at 50 °C for 24 h (entries 8-9).

Table S2. Optimization of conditions for synthesis of salt **4d-f.**

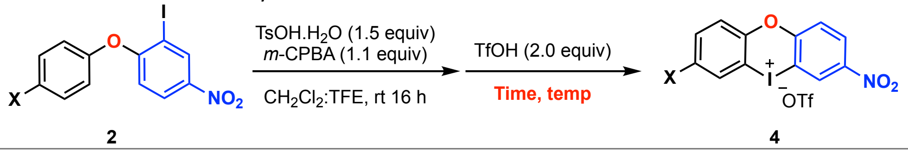

Entry	2	X	Temp (°C)	Time (h)	4	Yield (%)
**1**	**2d**	Br	rt	6	**4d**	19
**2**	**2d**	Br	rt	24	**4d**	78
**3**	**2d**	Br	50	6	**4d**	65
**4**	**2d**	Br	70	6	**4d-OTs**	74
**5**	**2d**	Br	70	6	**4d-OTs**	52[a]
**6**	**2e**	Cl	rt	24	**4e**	52
**7**	**2f**	F	rt	24	**4f**	30
**8**	**2e**	Cl	50	24	**4e**	88
**9**	**2f**	F	50	24	4f	71

All isolated yields. [a]: The solvents were evaporated after step 1, then MeCN was added together with TfOH.

Diaryl ethers with strong EDG's such as methoxy and phenoxy proved problematic and led to complex mixtures under the optimized conditions (Scheme S1a). We speculated that this was due to undesired oxidation of the electon rich aromatic system. To evaluate this further, we attempted to perform only the first step of the reaction, the synthesis of the Koser's intermediate (Scheme S1b). Even in the absence of TfOH and TFE, the reaction led to a complex mixture where no Koser's intermediate could be observed.

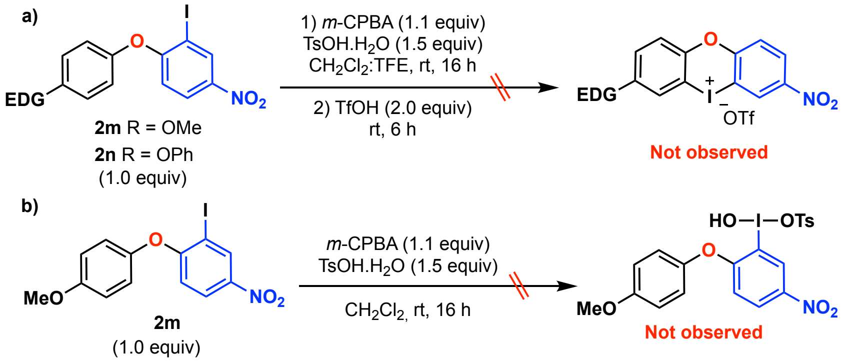

Scheme S1. Oxidation of electron rich substrate **2m-n**.

1.2 Optimization of the synthesis of acyclic salts 5

The optimization of reaction conditions for the synthesis of diaryliodonium salt **5a** from diaryl ether **2a** with anisole as dummy ligand is summarized in Table S3. With the conditions we found for preparation of the cyclic salts **4**, we anticpated that our target **5a** could be obtained from Koser derivative **3a** *via* a ligand exchange with anisole.[3] Indeed, the reaction of the isolated **3a** with anisole in TFE provided product **5a** in 74% yield, giving a overall yield of 69% over two steps (Scheme S2).

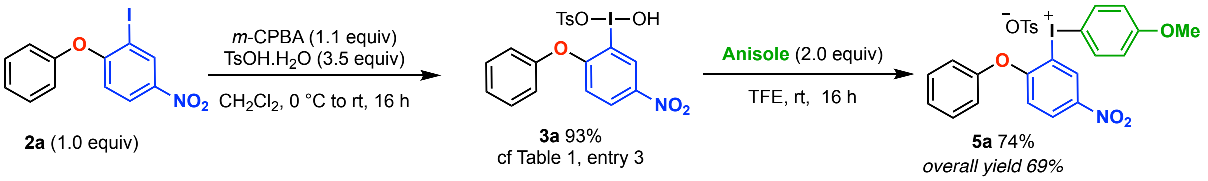

Scheme S2. Synthesis of iodonium salt **5a** via isolated Koser's reagent **3a**.

To facilitate the synthetic setup, we developed a one-pot two-step reaction by oxidation of **2a** in CH_2Cl_2 for 16 hours, followed by addition of anisole and TFE giving target **5a** in an excellent yield of 95% (Table S3, entry 1). Performing the reaction in CH_2Cl_2:TFE from the start fully suppressed the desired pathway, resulting in formation of the corresponding cyclic product **4a-OTs** with complete selectivity in 85% yield (entry 2). The TFE did not prove crucial for reaction, as excluding this solvent only reduced the yield slightly to 86% (entry 3). Decreasing the reaction time for the second step reduced the yield (entry 4), however decreased loading of the TsOH resulted in only a slight yield lowering (entry 5). At a late stage of the project we also discovered that the reaction could be performed as a one-pot one-step reaction by having the anisole present from the start of the reaction, giving the product **5a** in 86% yield (entry 6). With these results, we decided to evaluate the scope of the reaction under the conditions of entry 2, and use the conditions of entry 6 when needed (see below).

Table S3. Optimization of conditions for synthesis of salt **5a**.

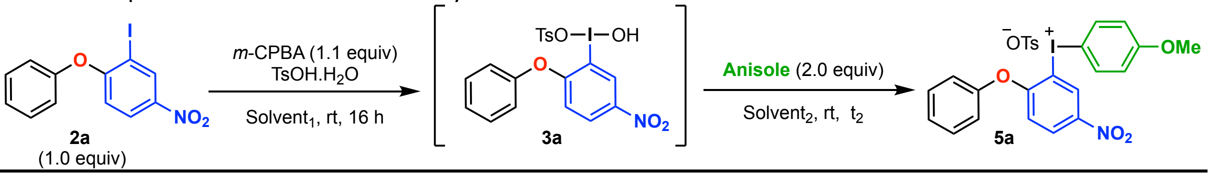

Entry	Solvent$_1$	TsOH (equiv)	Solvent$_2$	t_2 (h)	Yield 5a (%)
1	CH_2Cl_2	3.5	TFE	24	95
2	CH_2Cl_2:TFE (1:1)	3.5	--	24	0[a]
3	CH_2Cl_2	3.5	--	24	86
4	CH_2Cl_2	3.5	TFE	6	70
5	CH_2Cl_2	1.5	TFE	24	87
6	CH_2Cl_2	3.5	--	--	86[b]

All isolated yields. [a] Cyclic salt **4a-OTs** formed as only product in 85% yield. [b] Anisole (1.1 equiv) present from the start of the reaction, reaction time 16 h.

With some substrates the one-pot two-step approach was not as efficient, since these reactions led to mixtures of the desired product **5** and the competing cyclized product **4-OTs** (Scheme S3). This issue could be circumvented by performing the reaction in one step, with all reagents present from the start of the reaction (conditions of Table S3, entry 6). For instance, the reaction with ether **2b** under the two-step conditions provided a product mixture of **5i** and **4f-OTs** in 44% and 33% yield, respectively. Fortunately, when the reaction was performed with excess anisole present from the start of the reaction, product **5i** was obtained as the only product in 75% yield.

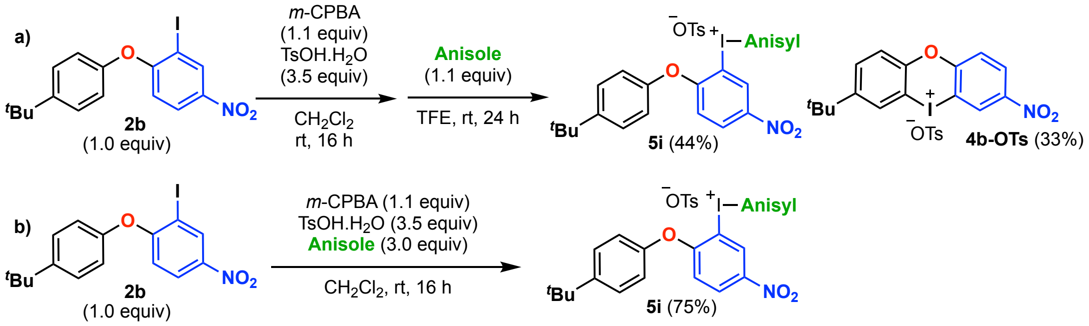

Scheme S3. Competing cyclization with some substrates

1.2.1 Reactions with mesitylene as dummy ligand

When attempting to synthesize salt **5b** with a mesitylene "dummy"- ligand under the optimized conditions, the product was obtained in an inseparable mixture with the cyclic salt **4a-OTs** (Scheme S2a) We repeated the reaction by having excess of mesitylene present from the start of the reaction, however this only led to decomposition and no product **5b** or **4a-OTs** was observed under these conditions (Scheme S4b). Fortunately, **5b** could be obtained as the only product in 81% yield from the reaction with the Koser's intermediate **3a** in TFE at room temperature for 16 hours (Scheme S4c).

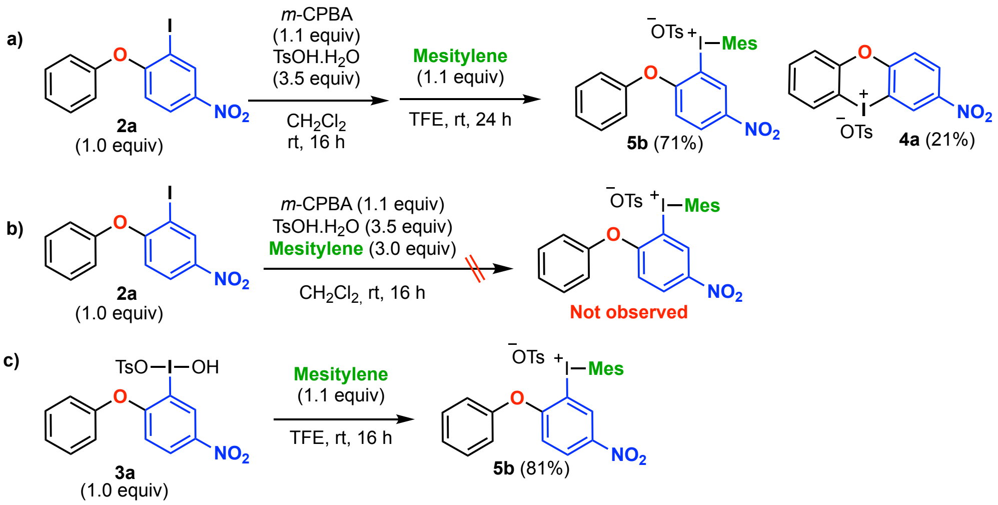

Scheme S4. Reactions with mesitylene as dummy ligand.

2. Limitations in applications of cyclic salt 4a

Nachtsheim and co-workers demonstrated the reactivity of their unsubstituted model product, diphenyliodaoxinium triflate, in a range of literature-based applications.[4] Inspired by this work, we demonstrated the utility of our salt **4a** in a series of atom-efficient transformations following literature precedent (Scheme 3). Unfortunately, the Cu-catalyzed amination with 4-chloroaniline proceeded with moderate chemoselectivity to deliver a 2:1 product mixture (Scheme S5a).[5] In comparison to the unsubstituted diphenyliodaoxinium triflate,[4] nitro-substituted salt **4a** gave similar yields in the transformations described in Scheme 3. To the contrary, no reactivity was observed in a metal-free bromination through thermolysis,[6] illustrating the influence of the electron-withdrawing nitro group (Scheme S5).

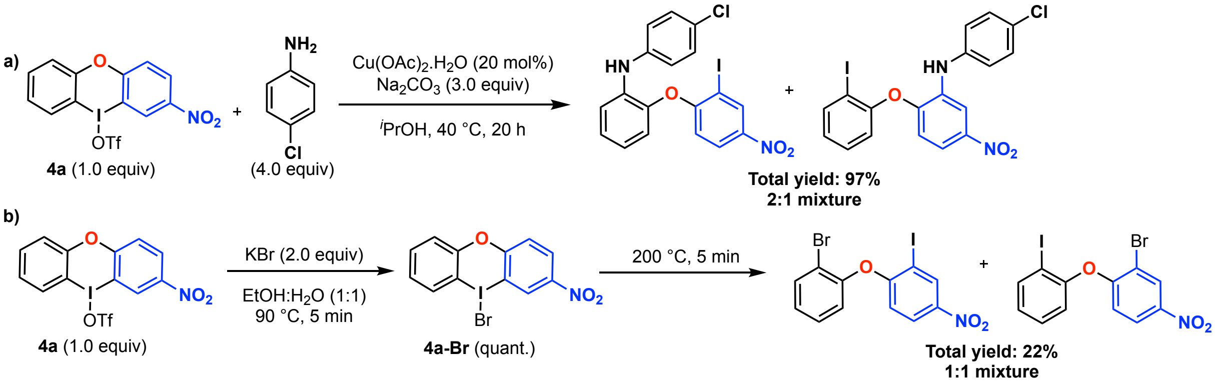

Scheme S5. Unproductive reactions with salt **4a**.

3. Synthesis of diaryliodonium salts 1

All diaryliodonium salts **1** used in this investigation were synthesized according to the one-pot procedures developed in the Olofsson group (GP1-2).[7] The synthesis was performed in round bottom flasks without any precautions to avoid moisture or air.

General procedure 1 (GP1):

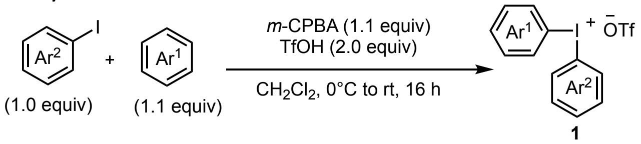

1

Following a literature procedure developed by Olofsson and co-workers for synthesis of diaryliodonium triflate salts.[7a] The aryl iodide (5.0 mmol, 1.0 equiv) and *m*-CPBA (5.5 mmol, 1.1 equiv) were dissolved in DCM (25 mL) in a round bottom flask. The mixture was cooled to 0 °C, followed by subsequent addition of TfOH (10.0 mmol, 2.0 equiv) with a Hamilton syringe. After the mixture had stopped fuming, the flask was brought to room temperature, followed by the addition of the arene (5.5 mmol, 1.1 equiv). The reaction mixture was stirred at ambient temperature for 16 hours. The solvent was evaporated under vacuum, and the product was precipitated by addition of Et_2O (50 mL). The mixture was stirred for an additional 10 min at room temperature before being stored in the freezer for 2-6 hours to allow full product precipitation. The product **1** was isolated by filtration on a glass-filter, washed with Et_2O (10 mL) and dried under vacuum.

General procedure 2 (GP2):

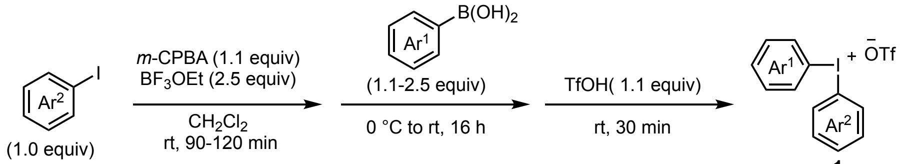

1

Following a literature procedure developed by Olofsson and co-workers for synthesis of diaryliodonium tetrafluoroborate salts.[7b] The iodoarene (1.0 equiv) and *m*-CPBA (1.1 equiv) were dissolved in DCM (0.2 M) followed by subsequent addition of boron trifluoride etherate (2.5 equiv). The mixture was stirred at room temperature for 90-120 minutes to allow oxidation of the aryl iodide. The suspension was cooled to 0 °C and the arylboronic acid (1.1-2.5 equiv) was added portion wise. The reaction was stirred at room temperature over night. When the reaction time had elapsed, the reaction was cooled to 0 °C followed by drop wise addition of TfOH (1.1 equiv). The reaction was stirred at ambient temperature for 30 additional min. The suspension was filtered through a short silica plug, and the crude mixture was eluted with DCM followed by DCM:MeOH (20:1). The latter solution was concentrated under vacuum and the product was triturated in Et_2O. The mixture was stirred vigorously for 10 minutes and was then stored in the freezer overnight. The product was isolated by filtration, washed with Et_2O and dried under vacuum.

All iodonium salts except **1c** were reported in our recent *Chem* paper,**[8]** the details of each synthesis is given in Table S4.

Table S4. Synthesis of reported diaryliodonium salts **1**

Diaryliodonium salt	GP	Temp	Time	Yield (%)	Ref
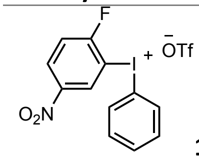 **1a**	**1**	**rt**	**16**	**89**	[8]
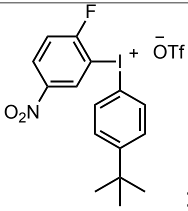 **1b**	**1**	**rt**	**16**	**73**	[8]
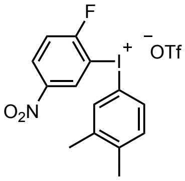 **1c**	**1**	**rt**	**16**	**94**	*Novel, analytical data given below*
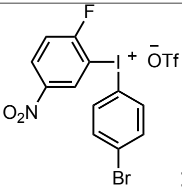 **1d**	**1**	**rt**	**16**	**64**	[8]
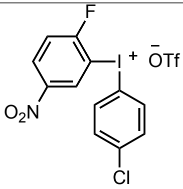 **1e**	**1**	**rt**	**16**	**70**	[8]
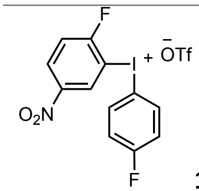 **1f**	**1**	**rt**	**16**	**93**	[8]
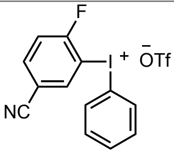 **1g**	**1**	**rt**	**16**	**80**	[8]
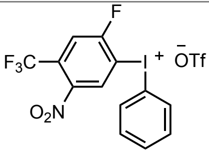 **1h**	**1**	**rt**	**16**	**83**	[8]
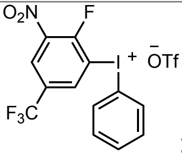 **1i**	**1**	**rt**	**16**	**61**	[8]

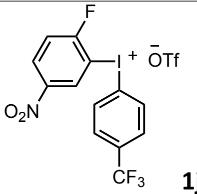 **1j**	**2**	**rt**	**16**	**49**	**[8]**
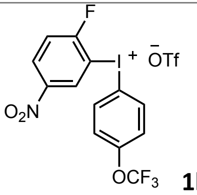 **1k**	**2**	**rt**	**16**	**24**	**[8]**
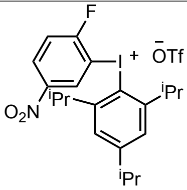 **1l**	**1**	**rt**	**16**	**66**	**[8]**
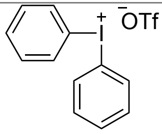 **18**	**1**	**rt**	**10 min**	**92**	**[7a]**

Synthesis of (2-fluoro-5-nitrophenyl)(3,4-dimethylphenyl)iodonium triflate (1c)

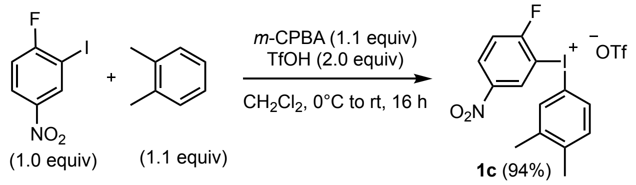

Synthesized from 1-fluoro-2-iodo-4-nitrobenzene (300 mg, 0.865 mmol, 1.0 equiv, 77%) and *o*-xylene (0.115 mL, 0.952 mmol, 1.1 equiv) according to GP1, giving product **1c** as an off-white solid (423 mg, 0.81 mmol, 94 %); m.p.: 133.4 °C; ^{1}H NMR (400 MHz, DMSO-d$_6$): δ 9.38 – 9.32 (m, 1H), 8.58 – 8.50 (m, 1H), 8.09 (s, 1H), 8.04 (d, J = 8.3 Hz, 1H), 7.83 (t, J = 8.3 Hz, 1H), 7.32 (d, J = 8.2 Hz, 1H), 2.25 (s, 6H); ^{13}C NMR (101 MHz, DMSO-d$_6$): δ 163.3 (d, J = 258.2 Hz), 145.3 (d, J = 2.9 Hz), 142.5, 141.5, 136.0, 133.3 (d, J = 8.4 Hz), 133.1, 133.0, 131.3 (d, J = 10.3 Hz), 121.2 (q, J = 322.2 Hz), 118.3 (d, J = 25.5 Hz), 114.0, 105.0 (d, J = 26.9 Hz), 19.8, 19.7; ^{19}F NMR (377 MHz, DMSO-d$_6$): δ -77.74 (s, 3F), -88.73 (s, 1F); HRMS (ESI): Calcd for $C_{14}H_{12}FINO_2$ [M-OTf]$^+$: 371.9891; found: 371.9894.

4. Synthesis of diaryl ethers 2 (GP3)

Our methodology for synthesis of diaryl ethers **2** from fluorinated diaryliodonium salts **1** by diarylation of water was recently reported (GP3).[8] Diaryl ethers **2a, 2c-j and 2l-n** were reported in that paper (Scheme S5) whereas **2c** and **2k** are novel and further described below.

General procedure 3 (GP3):

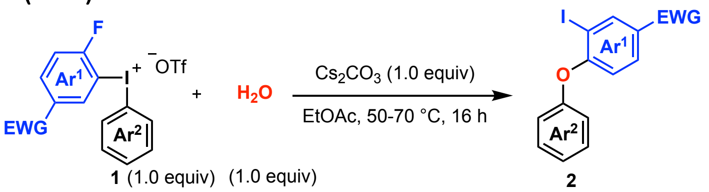

The diaryliodonium salt **1** (0.7 mmol, 1.0 equiv) and Cs_2CO_3 (0.7 mmol, 1.0 equiv) were added to an oven-dried microwave vial. The vial was sealed with a cap and the atmosphere was exchanged to argon. EtOAc (3.6 mL) was added to the vial followed by subsequent addition of the water (0.7 mmol, 1.0 equiv). *NOTE:* Care should be taken to not add any excess of water since this reduces the yield of the reaction significantly. The reaction mixture was transferred to a pre-heated oil bath, and stirred at 50-70 °C for 16 hours. The products were isolated by column chromatography without prior work up. The diaryl ethers were eluted with P:Et_2O or P:EtOAc.

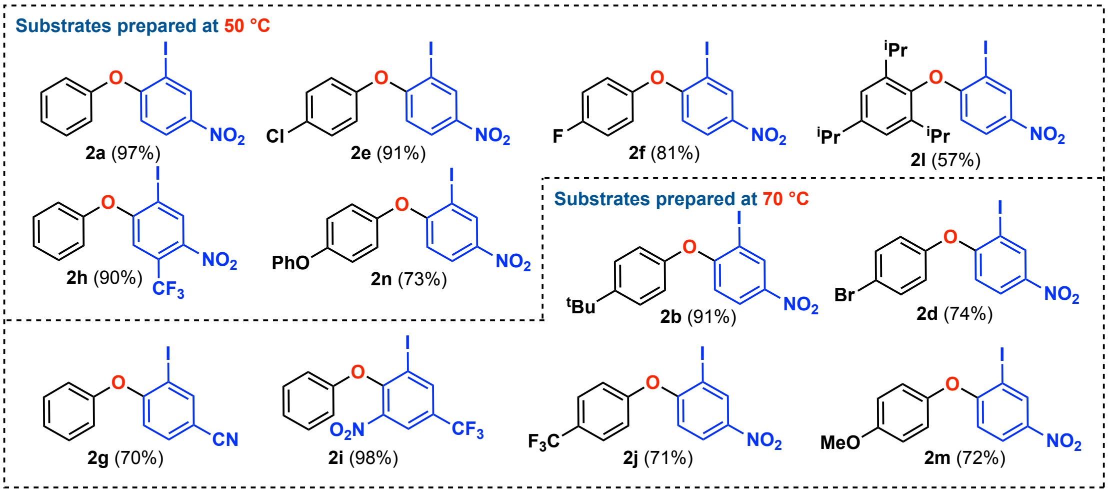

Scheme S5. Preparation of literature known ethers **2** by diarylation of water.

Synthesis of 1-(3,4-dimethylphenoxy)-2-iodo-4-nitrobenzene (2c)

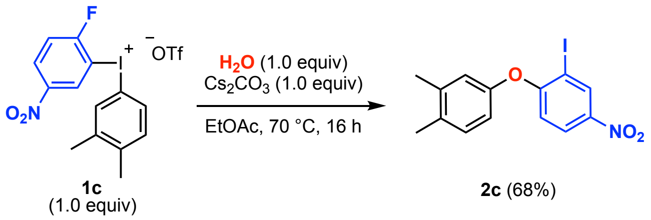

Diaryl ether **2c** was synthesized following a literature procedure developed by Olofsson and co-workers.[8] Water (0.010 mL, 0.58 mmol, 1.0 equiv) was arylated with diaryliodonium salt **1c** (300 mg, 0.58 mmol, 1.0 equiv) according to GP3 at 70 °C, giving product **2b** as a yellow solid (144 mg, 0.40 mmol, 68%); R_f = 0.28 in PE:Et_2O 98:2; m.p.: 78.8 °C; ^{1}H NMR (400 MHz, $CDCl_3$): δ 8.72 (d, J = 2.7 Hz, 1H), 8.09 (dd, J = 9.1, 2.7 Hz, 1H), 7.18 (d, J = 8.2 Hz, 1H), 6.88 (d, J = 2.6 Hz, 1H), 6.82 (dd, J = 8.2, 2.6 Hz, 1H), 6.71 (d, J = 9.1 Hz, 1H), 2.30 – 2.25 (m, 6H); ^{13}C NMR (101 MHz, $CDCl_3$): δ 163.2, 152.6, 142.9, 139.2, 135.6, 134.4, 131.3, 125.4, 121.6, 117.7, 114.9, 85.8, 20.1, 19.3; HRMS (ESI): Calcd for $C_{14}H_{12}INO_3$ $[M+Na]^+$: 391.9754; found: 391.9754.

Synthesis of 2-iodo-4-nitro-1-(4-(trifluoromethoxy)phenoxy)benzene (2k)

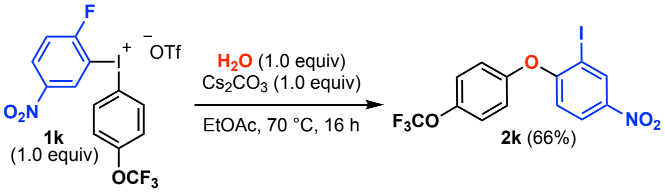

Diaryl ether **2k** was synthesized following a literature procedure developed by Olofsson and co-workers.[8] Water (0.012 mL, 0.69 mmol, 1.0 equiv) was arylated with diaryliodonium salt **1k** (400 mg, 0.69 mmol, 1.0 equiv) according to GP3 at 70 °C, giving product **2k** as a colorless oil (193 mg, 0.46 mmol, 66%); R_f = 0.25 in PE:Et_2O 98.5:1.5; ^{1}H NMR (400 MHz, $CDCl_3$): δ 8.76 (d, J = 2.6 Hz, 1H), 8.16 (dd, J = 9.1, 2.7 Hz, 1H), 7.30 (d, J = 8.5 Hz, 2H), 7.11 (d, J = 9.1 Hz, 2H), 6.79 (d, J = 9.1 Hz, 1H); ^{13}C NMR (101 MHz, $CDCl_3$): δ 162.1, 153.2, 146.4 (d, J = 1.9 Hz), 143.6, 135.7, 125.5, 123.3, 121.5, 120.5 (q, J = 257.0 Hz), 115.8, 86.5; ^{19}F NMR (377 MHz, $CDCl_3$): δ -58.18 (s, 3F); HRMS (ESI): Calcd for $C_{13}H_7F_3INO_4$ $[M+Na]^+$ 447.9264; found: 447.9259.

4.1 Alternative method to prepare diaryl ethers

Synthesis of 1-iodo-2-phenoxybenzene (19)

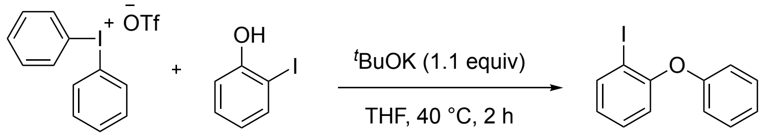

Following a literature procedure by Olofsson.[9] 2-iodophenol (220 mg, 1.0 mmol, 1.0 equiv) was dissolved in THF (4.3 mL) in a microwave vial and the atmosphere was exchanged to argon. The vial was placed in an ice bath and ^{t}BuOK (123 mg, 1.1 mmol, 1.1 equiv) was added at 0 °C. The suspension was stirred for 15 minutes before diaryliodonium salt **18** (645 mg, 1.5 mmol, 1.5 equiv) was added in one portion. The vial was transferred to a preheated oil bath set to 40 °C where the reaction was stirred for 2 hours. When the reaction time had elapsed, the product was purified by column chromatograpgy without prior work up, eluted with 100% P (rf = 0.43), to give product **19** as a colorless oil (264 mg, 0.089 mmol, 89%); ^{1}H NMR (400 MHz, $CDCl_3$) δ 7.86 (dd, *J* = 7.8, 1.6 Hz, 1H), 7.34 (dd, *J* = 8.7, 7.4 Hz, 2H), 7.29 (ddd, *J* = 8.2, 7.3, 1.6 Hz, 1H), 7.15 – 7.09 (m, 1H), 6.98 (dd, *J* = 8.7, 1.1 Hz, 2H), 6.92 – 6.83 (m, 2H); ^{13}C NMR (101 MHz, $CDCl_3$) δ 157.0, 156.6, 140.0, 130.0, 129.8, 125.5, 123.6, 119.6, 118.5, 89.0. Analytical data was in agreement with literature.[9]

5. Synthesis of cyclic diaryliodonium salts 4

5.1 General procedure 4 (GP4)

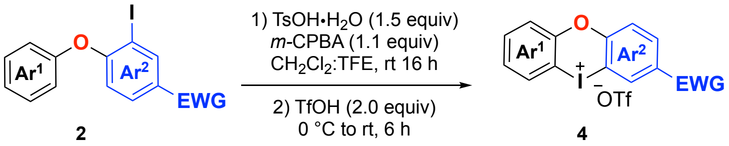

The diaryl ether **2** (1.0 equiv), *m*-CPBA (1.1 equiv), and TsOH·H_2O (1.5 equiv) were added to a round-bottomed flask followed by CH_2Cl_2 and TFE (1:1, 0.2 M). The reaction was stirred at room temperature for 16 hours. When the reaction time had elapsed, the reaction was cooled to 0 °C and TfOH (2.0 equiv) was added drop wise. The reaction was stirred at room temperature for 6 additional hours, before the solvent was removed *in vacuo* and the product was triturated in Et_2O. The mixture was stirred vigorously for 10 minutes in ether before being stored in the freezer for 2-6 hours to allow full product precipitation. The product **4** was isolated by filtration, washed with Et_2O and dried under vacuum.

5.2 Synthetic details and analytical data of 4

3-Nitrodibenzo[*b,e*][1,4]iodaoxin-5-ium triflate (4a)

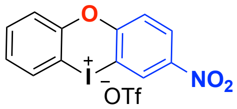

Diaryl ether **2a** (100 mg, 0.29 mmol, 1.0 equiv) was oxidized by *m*-CPBA (70 mg, 0.32 mmol, 1.1 equiv, 80%) in the presence of TsOH·H_2O (84 mg, 0.44 mmol, 1.5 equiv) according to GP4, giving the product **4a** as a white solid (140 mg, 0.286 mmol, 98%); ^{1}H NMR (400 MHz, DMSO-d_6): δ 8.87 (d, J = 2.6 Hz, 1H), 8.53 (dd, J = 9.0, 2.7 Hz, 1H), 8.06 (dd, J = 8.1, 1.4 Hz, 1H), 7.92 (d, J = 9.0 Hz, 1H), 7.80 – 7.70 (m, 2H), 7.51 (ddd, J = 8.5, 7.1, 1.7 Hz, 1H); ^{13}C NMR (101 MHz, DMSO-d_6): δ 158.0, 152.4, 145.4, 133.8, 133.5, 129.3, 128.8, 128.8, 122.2, 121.8, 120.7 (q, J = 321.2 Hz, $SO_2CF_3^-$), 102.3, 100.9; ^{19}F NMR (377 MHz, DMSO-d_6): δ -77.73 (s, 3F). The spectral data is in accordance with literature.[4]

3-(*Tert*-butyl)-7-nitrodibenzo[*b,e*][1,4]iodaoxin-5-ium triflate (4b)

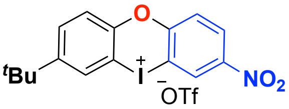

Diaryl ether **2b** (96 mg, 0.24 mmol, 1.0 equiv) was oxidized by *m*-CPBA (51 mg, 0.27 mmol, 1.1 equiv, 89%) in the presence of TsOH·H_2O (69 mg, 0.36 mmol, 1.5 equiv) according to GP4, giving the product **4b** as a white solid (83 mg, 0.15 mmol, 63%); m.p.: 182.0 - 183.5 °C (decomp.); ^{1}H NMR (400 MHz, DMSO-d_6): δ 8.86 (s, 1H), 8.52 (d, J = 8.7 Hz, 1H), 8.00 (s, 1H), 7.90 (d, J = 8.9 Hz, 1H), 7.75 (d, J = 7.7 Hz, 1H), 7.69 (d, J = 8.5 Hz, 1H), 1.31 (s, 9H); ^{13}C NMR (101 MHz, DMSO-d_6) δ 158.1, 151.6, 150.2, 145.3, 130.9, 129.7, 129.2, 128.7, 122.2, 121.2, 120.7 (q, J = 320.2 Hz, $SO_2CF_3^-$), 101.9, 100.5, 34.9, 30.9; ^{19}F NMR (377 MHz, DMSO-d_6): δ -77.74 (s, 3F); HRMS (ESI): Calcd for $C_{16}H_{15}INO_3$[M-OTf]$^+$: 396.0091; found: 396.0090.

2,3-Dimethyl-7-nitrodibenzo[*b,e*][1,4]iodaoxin-5-ium triflate (4c)

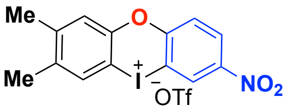

Diaryl ether **2c** (90 mg, 0.24 mmol, 1.0 equiv) was oxidized by *m*-CPBA (61 mg, 0.27 mmol, 1.1 equiv, 76%) in the presence of TsOH·H_2O (70 mg, 0.37 mmol, 1.5 equiv) according to a modified GP4, using 3.0 equiv of TfOH for the cyclization. Isolation of the product according to GP1 gave **4c** (61 mg, 0.12 mmol, 48%); m.p.: 239.5 °C (decomp.); ^{1}H NMR (400 MHz, DMSO-d_6): δ 8.86 (s, 1H), 8.52 (d, J = 8.2 Hz, 1H), 7.86 (d, J = 8.8 Hz, 1H), 7.76 (s, 1H), 7.59 (s, 1H), 2.32 (s, 3H), 2.29 (s, 3H); ^{13}C NMR (101 MHz, DMSO-d_6): δ 158.3, 150.4, 145.2, 143.2, 137.7, 132.6, 129.2, 128.7, 122.1, 120.7 (q, J = 322.8 Hz, $SO_2CF_3^-$), 102.0, 96.4, 19.2, 18.8; one aromatic carbon signal missing; ^{19}F NMR (377 MHz, DMSO-d_6): δ -77.74 (s, 3F); HRMS (ESI): Calcd for $C_{14}H_{11}INO_3$[M-OTf]$^+$: 367.9778; found: 367.9767.

2,3-Dimethyl-7-nitrodibenzo[*b,e*][1,4]iodaoxin-5-ium tosylate (4c-OTs)

The tosylate salt **4c-OTs** was obtained following according to GP5 (see Section 5.1) using diaryl ether **2c** (87 mg, 0.24 mmol, 1.0 equiv), *m*-CPBA (48.2 mg, 0.26 mmol, 1.1 equiv, 93%) and TsOH·H_2O (157 mg, 0.83 mmol, 3.5 equiv) in CH_2Cl_2 (1.2 mL). After 16 h, TFE (1.2 mL) and anisole (28 µL, 0.26 mmol, 1.1 equiv) were added, continued reaction for 24 h at rt. Isolation of the product according to GP5 gave **4c-OTs** (89 mg, 0.16 mmol, 70%);^{1}H NMR (400 MHz, DMSO-d_6): δ 8.86 (d, J = 2.6 Hz, 1H), 8.51 (dd, J = 8.9, 2.6 Hz, 1H), 7.86 (d, J = 9.0 Hz, 1H), 7.76 (s, 1H), 7.59 (s, 1H), 7.47 (d, J = 8.0 Hz, 1H), 7.10 (d, J = 7.9 Hz, 1H), 2.35 – 2.22 (m, 6H), 2.08 (s, 6H).

3-Bromo-7-nitrodibenzo[*b,e*][1,4]iodaoxin-5-ium triflate (4d)

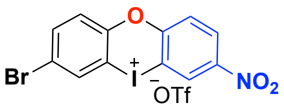

Diaryl ether **2d** (75 mg, 0.18 mmol, 1.0 equiv) was oxidized by *m*-CPBA (38 mg, 0.20 mmol, 1.1 equiv, 89%) in the presence of TsOH·H_2O (51 mg, 0.27 mmol, 1.5 equiv) according to a modified GP4, performing the cyclization for 24 h at room temperature. Isolation of the product according to GP1 gave **4d** as a white solid (79 mg, 0.14 mmol, 78%); m.p.: 179.6 - 180.4 °C (decomp.); ^{1}H NMR (400 MHz, DMSO-d_6): δ 8.86 (d, J = 2.3 Hz, 1H), 8.54 (dd, J = 9.0, 2.4 Hz, 1H), 8.19 (d, J = 1.4 Hz, 1H), 7.97 – 7.88 (m, 2H), 7.72 (d, J = 8.6 Hz, 1H); ^{13}C NMR (101 MHz, DMSO-d_6): δ 157.8, 152.0, 145.5, 136.4, 135.0, 129.1, 128.8, 123.4, 122.3, 120.7 (q, J = 322.2 Hz, $SO_2CF_3^-$), 119.4, 102.2, 102.1; ^{19}F NMR (377 MHz, DMSO-d_6) δ -77.73 (s, 3F); HRMS (ESI): Calcd for $C_{12}H_6BrINO_3$ [M-OTf]$^+$ 417.8570; found: 417.8573.

3-Chloro-7-nitrodibenzo[*b,e*][1,4]iodaoxin-5-ium triflate (4e)

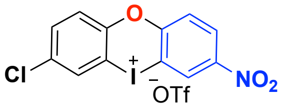

Diaryl ether **2e** (75 mg, 0.20 mmol, 1.0 equiv) was oxidized by *m*-CPBA (41 mg, 0.22 mmol, 1.1 equiv, 93%) in the presence of TsOH·H_2O (57 mg, 0.30 mmol, 1.5 equiv) according to a modified GP4, performing the cyclization for 24 h at 50 °C. Isolation of the product according to GP1 gave **4e** as a white solid (92 mg, 0.18 mmol, 88%); m.p.: 245.1 °C (decomp.); ^{1}H NMR (400 MHz, DMSO-d_6): δ 8.86 (d, J = 2.6 Hz, 1H), 8.54 (dd, J = 9.1, 2.7 Hz, 1H), 8.08 (d, J = 2.2 Hz, 1H), 7.92 (d, J = 9.0 Hz, 1H), 7.86 – 7.74 (m, 2H); ^{13}C NMR (101 MHz, DMSO-d_6): δ 158.3, 152.1, 146.0, 134.0, 132.8, 132.1, 129.6, 129.3, 123.5, 122.8, 102.7, 102.3; ^{19}F NMR (377 MHz, DMSO-d_6): δ -77.74 (s, 3F); HRMS (ESI): Calcd for $C_{12}H_6ClINO_3$ [M-OTf]$^+$: 373.9075; found: 373.9078.

3-Fluoro-7-nitrodibenzo[*b,e*][1,4]iodaoxin-5-ium triflate (4f)

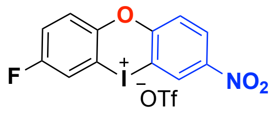

Diaryl ether **2f** (100 mg, 0.28 mmol, 1.0 equiv) was oxidized by *m*-CPBA (57 mg, 0.31 mmol, 1.1 equiv, 93%) in the presence of TsOH·H_2O (80 mg, 0.42 mmol, 1.5 equiv) according to a modified GP4, performing the cyclization for 24 h at 50 °C. Isolation of the product according to GP1 gave **4f** as a white solid (101 mg, 0.20 mmol, 71%); m.p.: 235.7 °C (decomp.); ^{1}H NMR (400 MHz, DMSO-d_6): δ 8.87 (d, J = 2.5 Hz, 1H), 8.55 (dd, J = 8.9, 2.7 Hz, 1H), 7.92 (d, J = 8.9 Hz, 2H), 7.84 (dd, J = 9.0, 4.5 Hz, 1H), 7.65 (td, J = 8.5, 2.8 Hz, 1H); ^{13}C NMR (101 MHz, DMSO-d_6): δ 160.1 (d, J = 248.6 Hz), 158.6, 149.8 (d, J = 2.6 Hz), 145.9, 129.6, 129.3, 123.4 (d, J = 8.5 Hz), 122.7, 121.2 (d, J = 23.6 Hz), 120.5 (d, J = 28.0 Hz), 103.2, 102.1 (d, J = 9.7 Hz); ^{19}F NMR (377 MHz, DMSO-d_6): δ -77.75 (s, 3F), -112.67 (s, 1F); HRMS (ESI): Calcd for $C_{12}H_6FINO_3$ [M-OTf]$^+$: 357.9371; found: 357.9367.

3-Cyanodibenzo[*b,e*][1,4]iodaoxin-5-ium triflate (4g)

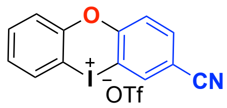

Diaryl ether **2g** (81 mg, 0.252 mmol, 1.0 equiv) was oxidized by *m*-CPBA (60 mg, 0.277 mmol, 1.1 equiv, 80%) in the presence of TsOH·H_2O (72 mg, 0.378 mmol, 1.5 equiv) according to GP4, giving the product **4g** as an off-white solid (0.106 g, 0.226 mmol, 90%); ^{1}H NMR (400 MHz, DMSO-d_6): δ 8.48 (d, *J* = 2.0 Hz, 1H), 8.20 (dd, *J* = 8.5, 2.0 Hz, 1H), 8.05 (dd, *J* = 8.2, 1.4 Hz, 1H), 7.89 (d, *J* = 8.5 Hz, 1H), 7.78 – 7.69 (m, 2H), 7.50 (ddd, *J* = 8.5, 6.9, 1.9 Hz, 1H); ^{13}C NMR (101 MHz, DMSO-d_6): δ 156.9, 152.6, 137.7, 137.5, 133.7, 133.4, 128.7, 122.4, 121.7, 116.8, 110.3, 102.7, 101.5; ^{19}F NMR (377 MHz, DMSO-d_6) δ -77.78; Spectral data is in agreement with literature.[4]

3-Nitro-4-trifluoromethyldibenzo[*b,e*][1,4]iodaoxin-5-ium tosylate (4h-OTs)

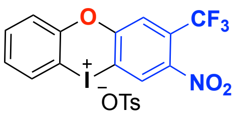

Diaryl ether **2h** (106 mg, 0.259 mmol, 1.0 equiv) was oxidized by *m*-CPBA (61 mg, 0.285 mmol, 1.1 equiv, 80%) in the presence of TsOH·H_2O (74 mg, 0.389 mmol, 1.5 equiv) according to a slightly modified GP4. No triflic acid was added and the reaction time was extended from 16 h to 30 h, giving the product **4h** as an off-white solid (0.138 g, 0.226 mmol, 92%); m.p.: 225.2-227.8 °C; ^1H NMR (400 MHz, DMSO-d_6): δ 8.81 (s, 1H), 8.40 (s, 1H), 8.06 (d, *J* = 7.7 Hz, 1H), 7.78 – 7.71 (m, 2H), 7.53 (ddd, *J* = 8.6, 5.7, 3.2 Hz, 1H), 7.47 (d, *J* = 8.1 Hz, 2H), 7.11 (d, *J* = 7.8 Hz, 2H), 2.28 (s, 3H); ^{13}C NMR (101 MHz, DMSO-d_6): δ 156.8, 152.3, 144.2, 133.8, 133.5, 131.4, 129.0, 128.1, 126.2, 125.9, 125.5, 122.5, 121.9, 121.4, 119.8, 117.0, 107.2, 101.2, 20.8; ^{19}F NMR (377 MHz, DMSO-d_6): δ -58.95; HRMS (ESI): Calcd for $C_{12}H_9INO_4$ $[M\text{-}OTs]^+$: 357.9571; found: 357.9585; HRMS (ESI): Calcd for $C_{13}H_6F_3INO_3$ $[M\text{-}OTs]^+$: 407.9939; found: 407.9930.

5-Nitro-3-trifluoromethyldibenzo[*b,e*][1,4]iodaoxin-5-ium tosylate (4i-OTs)

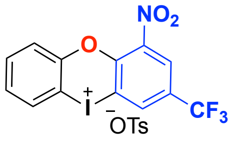

Diaryl ether **2i** (100 mg, 0.244 mmol, 1.0 equiv) was oxidized by *m*-CPBA (58 mg, 0.269 mmol, 1.1 equiv, 80%) in the presence of TsOH·H_2O (70 mg, 0.367 mmol, 1.5 equiv) according to a slightly modified GP4. No triflic acid was added and the reaction time was extended from 16 h to 30 h, giving the product **4i** as an off-white solid (0.124 g, 0.214 mmol, 88%); m.p.: 241.7-248.2 °C; ^1H NMR (400 MHz, DMSO-d_6): δ 8.76 (d, *J* = 2.1 Hz, 1H), 8.65 (d, *J* = 2.2 Hz, 1H), 8.10 (dd, *J* = 8.1, 1.5 Hz, 1H), 7.80 – 7.70 (m, 2H), 7.56 (ddd, *J* = 8.6, 7.0, 1.8 Hz, 1H), 7.47 (d, *J* = 8.0 Hz, 2H), 7.11 (d, *J* = 8.1 Hz, 2H), 2.28 (s, 3H); ^{13}C NMR (101 MHz, DMSO-d_6): δ 152.1, 149.2, 145.3, 141.7, 137.9, 135.0, 133.8, 133.6, 129.3, 128.1, 127.3 (q, *J* = 34.9 Hz), 125.8, 125.5, 122.0 (q, *J* = 273.2 Hz), 121.8, 107.3, 102.7, 20.8; ^{19}F NMR (377 MHz, DMSO-d_6): δ -60.97; HRMS (ESI): Calcd for $C_{12}H_9INO_4$ $[M\text{-}OTs]^+$: 357.9571; found: 357.9585; HRMS (ESI): Calcd for $C_{13}H_6F_3INO_3$ $[M\text{-}OTs]^+$: 407.9939; found: 407.9939.

6. Synthesis of acyclic diaryliodonium salts 5

The synthesis of iodonium salts **5a, 5c-5k** was performed according to General procedure 5 or 6, whereas iodonium salt **5b** was performed stepwise, through isolation of the Koser reagent as described in section 5.4.

6.1 General procedure 5 (GP5):

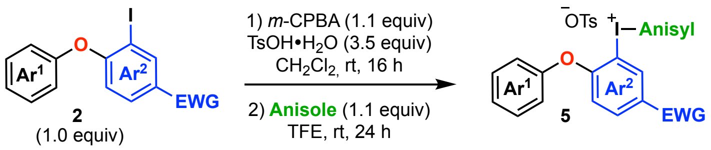

The diaryl ether **2** (1.0 equiv), *m*-CPBA (1.1 equiv), and TsOH·H_2O (3.5 equiv) were added to a round-bottomed flask followed by CH_2Cl_2 (0.2 M). The reaction mixture was stirred at room temperature for 16 hours. TFE (0.2 M) was added, followed by the dropwise addition of anisole (1.1 equiv). The reaction mixture was stirred at room temperature for 24 hours. Afterwards, the solvent was removed *in vacuo* and the product was triturated in Et_2O. The mixture was stirred vigorously for 10 minutes before being stored in the freezer for 2-6 hours. The product **5** was isolated by filtration, washed with Et_2O and dried under vacuum.

For *in situ* counterion exchange from OTs to OTf: Upon completion of the reaction; to the solution of compound **5** in CH_2Cl_2:TFE, TfOH (2.0 equiv) was added with a Hamilton syringe at 0 °C to the reaction mixture. The reaction was stirred for 30 min at room temperature. The purification was performed as described above by trituration in Et_2O.

6.2 General procedure 6 (GP6):

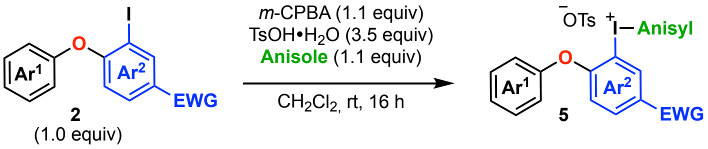

The diaryl ether **2** (1.0 equiv), *m*-CPBA (1.1 equiv), and TsOH·H_2O (3.5 equiv) were added to a round-bottomed flask followed by CH_2Cl_2 (0.2 M). Anisole (1.1-3.0 equiv) was added drop-wise and the reaction mixture was stirred at room temperature for 16 hours. Afterwards, the solvent was removed *in vacuo* and the product was triturated in Et_2O. The mixture was stirred vigorously for 10 minutes before being stored in the freezer for 2-6 hours. The product **5** was isolated by filtration, washed with Et_2O and dried under vacuum.

6.3 Synthetic details and analytical data of 5

(4-Methoxyphenyl)(5-nitro-2-phenoxyphenyl)iodonium tosylate (5a)

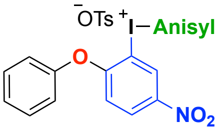

Diaryl ether **2a** (100 mg, 0.29 mmol, 1.0 equiv) was oxidized by *m*-CPBA (65 mg, 0.32 mmol, 1.1 equiv, 85%) in the presence of TsOH·H_2O (195 mg, 1.03 mmol, 3.5 equiv) in CH_2Cl_2 (1.5 mL) according to GP5. After 16 h, anisole (35 µL, 0.32 mmol, 1.1 equiv) and TFE (1.5 mL) was added, giving the product **5a** as a white solid (172 mg, 0.28 mmol, 95%); m.p.: 191.7 °C; ^{1}H NMR (400 MHz, DMSO-d_6): δ 9.37 (d, J = 2.7 Hz, 1H), 8.38 (dd, J = 9.1, 2.7 Hz, 1H), 8.17 (d, J = 8.9 Hz, 2H), 7.55 (t, J = 7.8 Hz, 2H), 7.46 (d, J = 8.0 Hz, 2H), 7.40 (t, J = 7.5 Hz, 1H), 7.09 (t, J = 9.0 Hz, 6H), 6.99 (d, J = 9.2 Hz, 1H), 3.80 (s, 3H), 2.28 (s, 3H). ^{13}C NMR (101 MHz, DMSO-d_6): δ 162.5, 160.6, 154.0, 146.1, 143.1, 138.1, 138.0, 133.5, 131.4, 130.5, 128.5, 127.1, 125.9, 121.0, 118.0, 116.7, 108.6, 106.0, 56.2, 21.2; HRMS (ESI): Calcd for $C_{19}H_{15}INO_4$ [M-OTs]$^+$: 448.0040; found: 448.0025.

[2-(4-Fluorophenoxy)-5-nitrophenyl](4-methoxyphenyl)iodonium tosylate (5c)

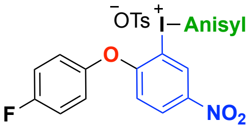

Diaryl ether **2f** (100 mg, 0.27 mmol, 1.0 equiv) was oxidized by *m*-CPBA (57 mg, 0.31 mmol, 1.1 equiv, 93%) in the presence of TsOH·H_2O (185 mg, 0.98 mmol, 3.5 equiv) in CH_2Cl_2 (1.4 mL) according to GP5. After 16 h, anisole (33 µL, 0.30 mmol, 1.1 equiv) and TFE (1.4 mL) were added, giving the product **5c** as a white solid (161 mg, 0.25 mmol, 91%); m.p.: 173.2 °C; ^{1}H NMR (400 MHz, DMSO-d$_6$): δ 9.36 (d, *J* = 2.7 Hz, 1H), 8.37 (dd, *J* = 9.2, 2.7 Hz, 1H), 8.18 (d, *J* = 9.0 Hz, 2H), 7.46 (d, *J* = 8.0 Hz, 2H), 7.39 (t, *J* = 8.7 Hz, 2H), 7.16 – 7.05 (m, 6H), 7.02 (d, *J* = 9.2 Hz, 1H), 3.80 (s, 3H), 2.28 (s, 3H); ^{13}C NMR (101 MHz, DMSO-d$_6$): δ 162.1, 161.1, 160.2, 158.7, 149.7 (d, *J* = 2.5 Hz), 145.5, 142.7, 137.7, 137.5, 133.0, 129.9, 128.1, 125.5, 122.6 (d, *J* = 9.0 Hz), 117.5 (d, J = 23.8 Hz), 116.1, 107.9, 105.5, 55.7, 20.8; ^{19}F NMR (377 MHz, DMSO-d$_6$): δ -115.78; HRMS (ESI): Calcd for $C_{19}H_{14}FINO_4$ [M-OTs]$^+$: 465.9946; found: 465.9956.

[2-(4-Chlorophenoxy)-5-nitrophenyl](4-methoxyphenyl)iodonium tosylate (5d)

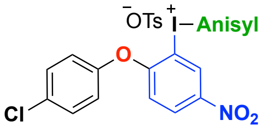

Diaryl ether **2e** (100 mg, 0.27 mmol, 1.0 equiv) was oxidized by *m*-CPBA (55 mg, 0.29 mmol, 1.1 equiv, 93%) in the presence of TsOH·H_2O (177 mg, 0.93 mmol, 3.5 equiv) in CH_2Cl_2 (1.3 mL) according to GP5. After 16 h, anisole (32 µL, 0.29 mmol, 1.1 equiv) and TFE (1.3 mL) were added, giving the product **5d** as a white solid (155 mg, 0.24 mmol, 89%); m.p.: 171.6 °C; ^{1}H NMR (400 MHz, DMSO-d$_6$): δ 9.36 (s, 1H), 8.37 (d, J = 7.8 Hz, 1H), 8.16 (d, J = 8.4 Hz, 2H), 7.59 (d, J = 8.3 Hz, 2H), 7.46 (d, J = 7.5 Hz, 2H), 7.15 – 7.02 (m, 7H), 3.79 (s, 3H), 2.28 (s, 3H); ^{13}C NMR (101 MHz, DMSO-d$_6$): δ 162.1, 159.8, 152.5, 145.4, 142.9, 137.7, 137.5, 133.0, 130.7, 130.5, 129.9, 128.1, 125.5, 122.3, 117.5, 116.7, 108.3, 105.5, 55.7, 20.8; HRMS (ESI): Calcd for $C_{19}H_{14}ClINO_4$ [M-OTs]$^+$: 481.9651; found: 481.9660.

[2-(4-Bromophenoxy)-5-nitrophenyl](4-methoxyphenyl)iodonium tosylate (5e)

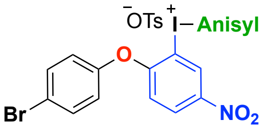

Diaryl ether **2d** (100 mg, 0.24 mmol, 1.0 equiv) was oxidized by *m*-CPBA (49 mg, 0.26 mmol, 1.1 equiv, 93%) in the presence of TsOH·H_2O (159 mg, 1.03 mmol, 3.5 equiv) in CH_2Cl_2 (1.2 mL) according to GP5. After 16 h, anisole (28 µL, 0.26 mmol, 1.1 equiv) and TFE (1.2 mL) were added, giving the product **5e** as a white solid (131 mg, 0.19 mmol, 79%); m.p.: 179.2 °C; ^{1}H NMR (400 MHz, DMSO-d$_6$): δ 9.37 (d, J = 2.5 Hz, 1H), 8.38 (dd, J = 9.2, 2.5 Hz, 1H), 8.16 (d, J = 8.9 Hz, 2H), 7.72 (d, J = 8.7 Hz, 2H), 7.46 (d, J = 7.8 Hz, 2H), 7.10 (d, J = 8.3 Hz, 3H), 7.05 (t, J = 8.2 Hz, 4H), 3.80 (s, 3H), 2.28 (s, 3H); ^{13}C NMR (101 MHz, DMSO-d$_6$): δ 162.1, 159.6, 153.1, 145.5, 142.9, 137.7, 137.5, 133.6, 133.0, 129.9, 128.0, 125.5, 122.7, 118.6, 117.5, 116.7, 108.4, 105.5, 55.7, 20.8; HRMS (ESI): Calcd for $C_{19}H_{14}BrINO_4$ [M-OTs]$^+$: 525.9145; found: 525.9135.

(5-Nitro-2-[4-(trifluoromethyl)phenoxy]phenyl)(4-methoxyphenyl)iodonium tosylate (5f)

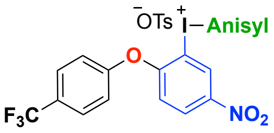

Diaryl ether **2j** (100 mg, 0.24 mmol, 1.0 equiv) was oxidized by *m*-CPBA (50 mg, 0.27 mmol, 1.1 equiv, 92.9 %) in the presence of TsOH·H_2O (163 mg, 0.86 mmol, 3.5 equiv) in CH_2Cl_2 (1.2 mL) according to GP5. After 16 h, anisole (29 µL, 0.27 mmol, 1.1 equiv) and TFE (1.2 mL) were added, giving the product **5f** as a white solid (126 mg, 0.18 mmol, 75%); m.p.: 175.8 °C; ^{1}H NMR (400 MHz, DMSO-d$_6$): δ 9.39 (d, J = 2.7 Hz, 1H), 8.40 (d, J = 8.8 Hz, 1H), 8.15 (d, J = 8.5 Hz, 2H), 7.89 (d, J = 8.3 Hz, 2H), 7.45 (d, J = 7.8 Hz, 2H), 7.32 – 7.17 (m, 3H), 7.09 (d, J = 7.6 Hz, 2H), 7.03 (d, J = 8.6 Hz, 2H), 3.78 (s, 3H), 2.28 (s, 3H); ^{13}C NMR (101 MHz, DMSO-d$_6$): δ 162.0, 159.0, 157.0, 145.4, 143.4, 137.8, 137.5, 133.1, 129.9, 128.1, 128.1, 126.5 (q, *J* = 32.2 Hz), 125.5, 123.9 (q, *J* = 272.0 Hz), 120.7, 117.9, 117.4, 109.2, 105.5, 55.7, 20.8; ^{19}F NMR (377 MHz, DMSO-d$_6$): δ -60.58 (s, 3F); HRMS (ESI): Calcd for $C_{20}H_{14}F_3INO_4$ [M-OTs]$^+$: 515.9914; found: 515.9910.

(5-Nitro-2-[4-(trifluoromethoxy)phenoxy]phenyl}(4-methoxyphenyl)iodonium tosylate (5g)

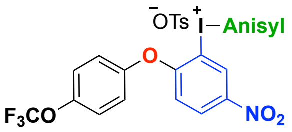

Diaryl ether **2k** (100 mg, 0.24 mmol, 1.0 equiv) was oxidized by *m*-CPBA (48 mg, 0.26 mmol, 1.1 equiv, 93%) in the presence of $TsOH{\cdot}H_2O$ (157 mg, 0.82 mmol, 3.5 equiv) in CH_2Cl_2 (1.2 mL) according to GP5. After 16 h, anisole (28 µL, 0.26 mmol, 1.1 equiv) and TFE (1.2 mL) were added, giving the product **5g** as a white solid (119 mg, 0.17 mmol, 72%); m.p.: 177.2 °C; ^{1}H NMR (400 MHz, DMSO-d_6): δ 9.38 (d, *J* = 2.5 Hz, 1H), 8.39 (dd, *J* = 9.1, 2.5 Hz, 1H), 8.17 (d, *J* = 8.9 Hz, 2H), 7.55 (d, *J* = 8.5 Hz, 2H), 7.46 (d, *J* = 7.9 Hz, 2H), 7.21 (d, *J* = 8.9 Hz, 2H), 7.14 – 7.03 (m, 5H), 3.79 (s, 3H), 2.28 (s, 3H); ^{13}C NMR (101 MHz, DMSO-d_6): δ 162.1, 159.6, 152.4, 145.9, 145.6, 143.0, 137.6, 137.5, 133.0, 130.0, 128.0, 125.5, 123.6, 122.2, 120.0 (q, J = 256.4 Hz), 117.5, 116.8, 108.4, 105.5, 55.7, 20.8; ^{19}F NMR (377 MHz, DMSO-d_6): δ -57.06 (s, 3F); HRMS (ESI): Calcd for $C_{20}H_{14}F_3INO_5$ [M-OTs]$^+$: 531.9863; found: 531.9863.

[5-Nitro-2-(2,4,6-triisopropylphenoxy)phenyl](4-methoxyphenyl)iodonium tosylate (5h)

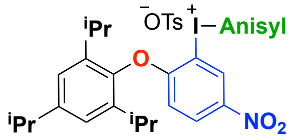

Diaryl ether **2l** (91 mg, 0.20 mmol, 1.0 equiv) was oxidized by *m*-CPBA (40 mg, 0.22 mmol, 1.1 equiv, 93%) in the presence of $TsOH{\cdot}H_2O$ (130 mg, 0.68 mmol, 3.5 equiv) in CH_2Cl_2 (1.0 mL) according to GP5. After 16 h, anisole (23 µL, 0.22 mmol, 1.1 equiv) and TFE (1.0 mL) were added, giving the product **5h** as a white solid (104 mg, 0.14 mmol, 71%); ***NOTE***: The product was stored in the freezer for 72 hours to assure full precipitation of the product; m.p.: 159.8 °C; ^{1}H NMR (400 MHz, DMSO-d_6): δ 9.44 (d, J = 2.7 Hz, 1H), 8.40 (dd, J = 9.2, 2.8 Hz, 1H), 8.15 (d, J = 9.0 Hz, 2H), 7.46 (d, J = 8.0 Hz, 2H), 7.19 (s, 2H), 7.13 – 7.06 (m, 4H), 6.65 (d, J = 9.3 Hz, 1H), 3.80 (s, 3H), 2.94 (hept, J = 6.8 Hz, 1H), 2.43 (hept, J = 6.8 Hz, 2H), 2.28 (s, 3H), 1.23 (d, J = 6.9 Hz, 6H), 1.08 (d, J = 6.8 Hz, 6H), 0.91 (d, J = 6.9 Hz, 6H); ^{13}C NMR (101 MHz, DMSO-d_6): δ 162.0, 160.7, 147.8, 145.7, 144.5, 142.2, 139.9, 137.6, 137.3, 133.7, 130.3, 128.0, 125.5, 123.0, 117.4, 113.9, 105.6, 104.6, 55.7, 33.4, 26.4, 24.2, 24.0, 22.7, 20.8; HRMS (ESI): Calcd for $C_{28}H_{33}INO_4$ [M-OTs]$^+$: 574.1449; found: 574.1444.

(2-[4-(*Tert*-butyl)phenoxy]-5-nitrophenyl)(4-methoxyphenyl)iodonium tosylate (5i)

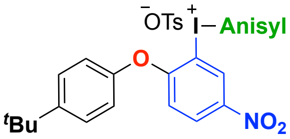

Diaryl ether **2b** (75 mg, 0.19 mmol, 1.0 equiv), was oxidized by *m*-CPBA (42 mg, 0.21 mmol, 1.1 equiv, 85%) in the presence of $TsOH{\cdot}H_2O$ (126 mg, 0.66 mmol, 3.5 equiv) in CH_2Cl_2 (1 mL) according to GP6. Anisole was added (62 µL, 0.57 mmol, 3.0 equiv) and the reaction was stirred for 16 h, giving product **5i** as a white solid (96 mg, 0.14 mmol, 75%); m.p.: 171.5 °C; ^{1}H NMR (400 MHz, DMSO-d_6): δ 9.36 (d, J = 2.7 Hz, 1H), 8.38 (dd, J = 9.2, 2.8 Hz, 1H), 8.17 (d, J = 8.6 Hz, 2H), 7.55 (d, J = 8.4 Hz, 2H), 7.46 (d, J = 7.7 Hz, 2H), 7.09 (t, J = 8.5 Hz, 4H), 6.99 (t, J = 8.3 Hz, 3H), 3.80 (s, 3H), 2.28 (s, 3H), 1.32 (s, 9H); ^{13}C NMR (101 MHz, DMSO-d_6): δ 162.5, 160.7, 151.7, 149.5, 146.0, 143.0, 138.1, 138.0, 133.6, 130.5, 128.5, 128.0, 125.9, 120.4, 117.9, 116.5, 108.4, 105.9, 56.2, 34.9, 31.6, 21.2; HRMS (ESI): Calcd for $C_{23}H_{29}INO_4$ [M-OTs]$^+$: 504.0666; found: 504.0664.

(4-Methoxyphenyl)(5-cyano-2-phenoxyphenyl)iodonium tosylate (5j)

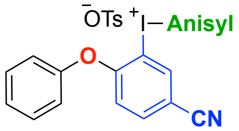

Diaryl ether **2g** (140 mg, 0.436 mmol, 1.0 equiv) was oxidized by *m*-CPBA (103 mg, 0.480 mmol, 1.1 equiv, 80%) in the presence of $TsOH{\cdot}H_2O$ (290 mg, 1.56 mmol, 3.5 equiv) in CH_2Cl_2 (2.2 mL) according to GP6. Anisole was added (52 µL, 0.480 mmol, 1.1 equiv) and the reaction was stirred for 16 h, giving product **5j** as an off-white solid (215 mg, 0.359 mmol, 83%); m.p.: 151.2-155.6 °C; ^{1}H NMR (400 MHz, DMSO-d_6): δ 9.01 (d, *J* = 2.1 Hz, 1H), 8.15 – 8.09 (m, 2H), 8.02 (dd, *J* = 8.7, 2.0 Hz, 1H), 7.53 (t, *J* = 7.9 Hz, 2H), 7.47 (d, *J* = 8.1 Hz, 2H), 7.38 (t, *J* = 7.4 Hz, 1H), 7.14 – 7.01 (m, 6H), 6.96 (d, *J* = 8.7 Hz, 1H), 3.80 (s, 3H), 2.29 (s, 3H); ^{13}C NMR (101 MHz, DMSO-d_6): δ 162.1, 158.6, 153.5, 146.0, 141.4, 138.7, 137.6, 137.4, 130.8, 128.0, 126.4, 125.5, 120.4, 117.6, 117.0, 117.0, 108.8, 107.2, 105.3, 55.7, 20.8; HRMS (ESI): Calcd for $C_{20}H_{15}INO_2$ [M-OTs]$^+$: 428.0134; found: 428.0142.

(4-Methoxyphenyl)(4-trifluoromethyl-5-nitro-2-phenoxyphenyl)iodonium tosylate (5k)

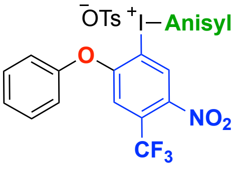

Diaryl ether **2h** (100 mg, 0.244 mmol, 1.0 equiv) was oxidized by *m*-CPBA (58 mg, 0.269 mmol, 1.1 equiv, 80%) in the presence of TsOH·H_2O (163 mg, 0.856 mmol, 3.5 equiv) in CH_2Cl_2 (1.2 mL) according to GP6. Anisole was added (29 µL, 0.269 mmol, 1.1 equiv) and the reaction was stirred for 16 h, giving product **5k** as a white solid (136 mg, 0.198 mmol, 81%); m.p.: 171.9-175.2 °C; ^{1}H NMR (400 MHz, DMSO-d$_6$): δ 9.36 (s, 1H), 8.16 (d, *J* = 8.8 Hz, 2H), 7.54 (t, *J* = 7.8 Hz, 2H), 7.46 (d, *J* = 7.7 Hz, 2H), 7.40 (t, *J* = 7.4 Hz, 1H), 7.22 (br s, 1H), 7.10 (q, *J* = 9.4, 8.9 Hz, 6H), 3.80 (s, 3H), 2.28 (s, 3H); ^{13}C NMR (101 MHz, DMSO-d$_6$): δ 162.2, 158.3, 153.5, 1419, 137.7, 135.1, 130.9, 128.0, 126.7, 125.5, 122.6, 120.1, 119.8, 117.6, 115.4, 112.1, 105.5, 55.8, 20.8. two carbon signals missing, one aromatic and the CF_3; ^{19}F NMR (377 MHz, DMSO-d$_6$): δ -59.53; HRMS (ESI): Calcd for $C_{20}H_{14}F_3INO_4$ [M-OTs]$^+$: 515.9903; found: 515.9914.

(4-methoxyphenyl)(2-phenoxyphenyl)iodonium tosylate (11)

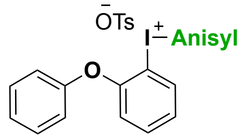

Diaryl ether **19** (46 mg, 0.155 mmol, 1.0 equiv) was oxidized by *m*-CPBA (37 mg, 0.171 mmol, 1.1 equiv, 80%) in the presence of TsOH·H_2O (103 mg, 0.544 mmol, 3.5 equiv) in CH_2Cl_2 (0.8 mL) according to GP6. Anisole was added (51 µL, 0.466 mmol, 3.0 equiv) and the reaction was stirred for 16 h, giving product **11** as a off-white solid (66 mg, 0.115 mmol, 74%); m.p.: 118.9-122.2 °C; ^{1}H NMR (400 MHz, DMSO-d$_6$): δ 8.46 – 8.40 (m, 1H), 8.03 (d, *J* = 8.7 Hz, 2H), 7.61 (t, *J* = 7.8 Hz, 1H), 7.49 (d, *J* = 8.1 Hz, 4H), 7.27 (dt, *J* = 15.2, 7.5 Hz, 2H), 7.12 (d, *J* = 7.8 Hz, 3H), 7.07 – 6.94 (m, 4H), 3.79 (s, 3H), 2.29 (s, 3H); ^{13}C NMR (101 MHz, DMSO-d$_6$) δ 161.9, 155.0, 154.9, 145.6, 137.7, 137.5, 137.2, 134.8, 130.5, 128.1, 125.9, 125.5, 125.2, 119.4, 118.1, 117.4, 109.3, 105.1, 55.7, 20.8.HRMS (ESI): Calcd for $C_{19}H_{16}IO_2$ [M-OTs]$^+$: 403.0189; found: 403.0187

6.4 Stepwise synthesis of mesityl salt 5b

Hydroxy(5-nitro-2-phenoxyphenyl)-λ^3-iodaneyl tosylate (3a)

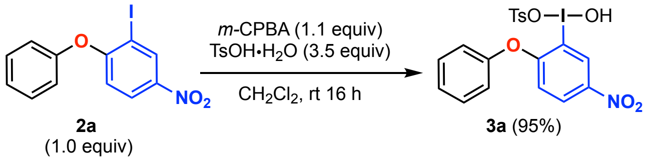

Diaryl ether **2a** (100 mg, 0.29 mmol, 1.0 equiv), *m*-CPBA (65 mg, 0.32 mmol, 1.1 equiv, 85%), and TsOH·H_2O (195 mg, 1.03 mmol, 3.5 equiv) were added to a round-bottomed flask followed by CH_2Cl_2 (1.5 mL). The reaction mixture was stirred at room temperature for 16 hours. Afterwards, the solvent was removed *in vacuo* and the product was triturated in Et_2O. The mixture was stirred vigorously for 10 minutes before being stored in the freezer for 2 hours. The product was isolated by filtration, washed with Et_2O and dried under vacuum, to give **3a** as an off-white solid (148 mg, 0.28 mmol, 95%); m.p.: 210.5 °C (decomp.); ^{1}H NMR (400 MHz, CD_3OD): δ 9.36 (d, J = 2.7 Hz, 1H), 8.52 (dd, J = 9.2, 2.7 Hz, 1H), 7.64 (d, J = 7.9 Hz, 2H), 7.57 (t, J = 7.9 Hz, 2H), 7.42 (t, J = 7.4 Hz, 1H), 7.27 (d, J = 8.1 Hz, 2H), 7.24 – 7.16 (m, 3H), 2.36 (s, 3H); ^{13}C NMR (101 MHz, CD_3OD): δ 161.5, 154.6, 143.5, 142.1, 141.4, 134.5, 131.8, 131.4, 129.2, 127.4, 126.3, 121.3, 116.4, 113.6, 20.6; HRMS (ESI): Calcd for $C_{12}H_9INO_4$ [M-OTs]$^+$: 357.9571; found: 357.9585.

Mesityl(5-nitro-2-phenoxyphenyl)iodonium tosylate (5b)

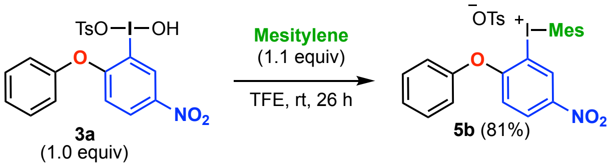

Koser's derivate **3a** (150 mg, 0.283 mmol, 1.0 equiv) was dissolved in TFE (1.4 mL) in a round bottom flask that was placed in an ice bath. Mesitylene (0.043 mL, 0.312 mmol, 1.1 equiv) was added drop wise at 0 °C and the reaction was left to stir at room temperature for 16 hours. Afterwards, the solvent

was removed *in vacuo* and the product was triturated in Et_2O. The mixture was stirred vigorously for 10 minutes before being stored in the freezer for 2 hours. The product was isolated by filtration, washed with Et_2O and dried under vacuum to give product **5b** as an off-white solid (145 mg, 0.239 mmol, 81%); m.p.: 185.6-187.4 °C; ^{1}H NMR (400 MHz, DMSO-d_6): δ 9.23 (s, 1H), 8.38 (dd, *J* = 9.3, 2.7 Hz, 1H), 7.46 (d, *J* = 7.9 Hz, 4H), 7.35 (t, *J* = 7.4 Hz, 1H), 7.20 – 7.05 (m, 4H), 6.97 (d, *J* = 9.2 Hz, 1H), 6.83 (d, *J* = 7.9 Hz, 2H), 2.57 (s, 6H), 2.28 (s, 3H), 2.27 (s, 3H); ^{13}C NMR (101 MHz, DMSO-d_6): δ 160.4, 153.2, 143.0, 142.8, 142.0, 137.6, 133.5, 130.8, 129.9, 129.7, 128.0, 126.6, 125.5, 122.1, 120.0, 116.8, 105.1, 26.0, 20.8, 20.4. One aromatic carbon signal missing; HRMS (ESI): Calcd for $C_{21}H_{19}INO_3$ $[M-OTs]^+$: 460.0404; found: 460.0393.

7. Derivatization of products 4a and 5a-OTf

Synthesis of 2-nitrodibenzo[*b*,*d*]furan (6)

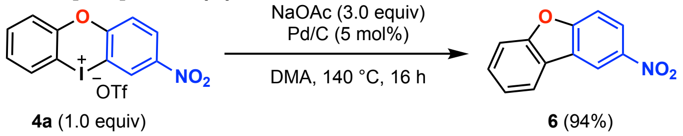

Product **6** was synthesized following a procedure reported by Panda and co-workers.[10] Diaryliodonium salt **4a** (49 mg, 0.1 mmol, 1.0 equiv), Pd/C (1.8 mg, 5.00 µmol, 5 mol%) and NaOAc (25 mg, 0.3 mmol, 3.0 equiv) were dissolved in DMA (2 mL) in a microwave vial. The vial was capped and the reaction was stirred at 140 °C for 16 h. When the reaction time had elapsed, the mixture was filtered throguh a short celite plug, eluted with EtOAc. Water was added (10 mL) and the layers were separated. The organic phase was washed with EtOAc (2 x 5 mL), dried over Na_2SO_4 and the solvent was concentrated *in vacuo*. The product was isolated by column chromatography, eluted with P:Et_2O 99:1 to give compound **9** as a colorless oil (20 mg, 0.094 mmol, 94%); R_f = 0.30 in P: Et_2O 99:1; ^1H NMR (400 MHz, $CDCl_3$): δ 8.87 (d, *J* = 2.4 Hz, 1H), 8.40 (dd, *J* = 9.0, 2.4 Hz, 1H), 8.03 (dd, *J* = 7.7, 1.3 Hz, 1H), 7.66 – 7.61 (m, 2H), 7.57 (ddd, *J* = 8.3, 7.1, 1.3 Hz, 1H), 7.45 (td, *J* = 7.5, 1.1 Hz, 1H); ^{13}C NMR (101 MHz, $CDCl_3$): δ 159.3, 157.6, 129.1, 125.2, 124.1, 123.2, 123.1, 121.4, 117.3, 112.4, 112.1. one carbon signal missing; The spectral data is in agreement with literature.[11]

Synthesis of triethylammonium benzylcarbamodithioate

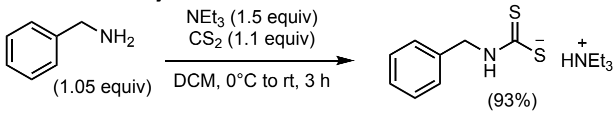

Triethylammonium benzylcarbamodithioate was synthesized following a literature procedure developed by Wen and co-workers.[12] To a solution of benzylamine (0.23 mL, 0.23 g, 2.10 mmol, 1.05 equiv) and Et_3N (0.28 mL, 0.20 g, 2.00 mmol, 1.0 equiv) in CH_2Cl_2 (5 mL), CS_2 (0.13 mL, 0.17 g, 2.20 mmol, 1.1 equiv) was added drop wise at 0 °C. The solution was stirred at room temperature for 3 hours, after which the solvent was removed *in vacuo*. The product was triturated in THF. After drying under vacuum, the dithiocarbamate salt was obtained as white crystals (0.53 g, 1.87 mmol, 93%); ^1H NMR (400 MHz, $CDCl_3$): δ 7.72 (br s, 1H), 7.38 – 7.26 (m, 4H), 7.26 – 7.20 (m, 1H), 4.82 (d, J = 5.4 Hz, 2H), 3.28 (q, J = 7.3 Hz, 6H), 1.42 (t, J = 7.3 Hz, 9H). The spectral data is in accordance with literature.[12-13]

Synthesis of 2-nitrophenoxathiine (7)

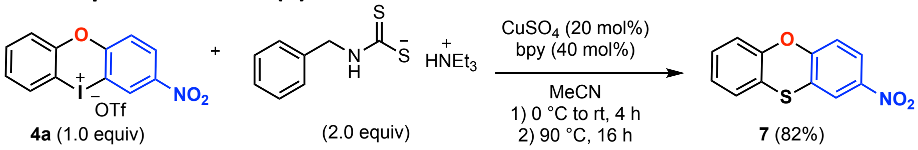

Product **7** was synthesized according to a literature procedure developed by Wen and co-workers.[12] Diaryliodonium salt **4a** (49 mg, 0.10 mmol, 1.0 equiv), triethylammonium benzylcarbamodithioate (57 mg, 0.20 mmol, 2.0 equiv), $CuSO_4$ (5.0 mg, 0.02 mmol, 0.2 equiv), and 2,2'-bipyridine (7 mg, 0.04 mmol, 0.4 equiv) were added to a microwave vial. The atmosphere was exchanged to argon and anhydrous MeCN (1.0 mL) was added. The reaction mixture was stirred at room temperature for 4 hours before being transferred to an oil bath set at 90 °C, where it was stirred for 16 hours. Afterwards, H_2O was added to the reaction mixture and the product was extracted with EtOAc (3 x 10 mL). The combined organic layers were washed with brine, dried over Na_2SO_4 and concentrated *in vacuo.* The crude product was purified by column chromatography (PE:EtOAc 4:1) to yield the product **7** as a yellow solid (20 mg, 0.08 mmol, 82%). R_f = 0.17 in PE:EtOAc 4:1; m.p.: 150.2 °C; ^1H NMR (400 MHz, $CDCl_3$): δ 8.03 – 7.95 (m, 2H), 7.17 (ddd, J = 8.0, 5.7, 3.3 Hz, 1H), 7.09 – 6.98 (m, 4H); ^{13}C NMR (101 MHz, $CDCl_3$): δ 156.8, 150.7, 144.5, 128.6, 126.9, 125.8, 123.7, 122.5, 121.9, 118.1, 118.0, 117.8; HRMS (ESI): Calcd for $C_{12}H_7NO_3S$ $[M+Na]^+$: 268.0039; found: 268.0030

Synthesis of 2-iodo-1-(2-iodophenoxy)-4-nitrobenzene (8)

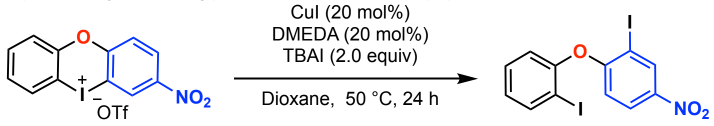

4a (1.0 equiv) **8** (71%)

Product **9** was synthesized following a procedure reported by Yoshikai and co-workers.[14] Diaryliodonium salt **4a** (49 mg, 0.1 mmol, 1.0 equiv), CuI (4 mg, 20.0 µmol, 0.20 equiv), and TBAI (74 mg, 0.2 mmol, 2.0 equiv) were dissolved in 1 mL dioxane in a microwave vial. The vial was capped, and DMEDA (3 µL, 20.0 µmol, 0.20 equiv) was added with a micro-syringe before the reaction was transferred to a pre-heated oil bath set to 50 °C, where it was stirred for 24 hours. When the reaction time had elapsed, the crude was filtered through a short celite plug eluted with Et_2O. The product was purified by column chromatography by dry loading on silica, eluted with P:Et_2O 98:2, to give the product as a light-brown oil (33 mg, 0.071 mmol, 71%); R_f = 0.27 in PE: Et_2O 98:2; ^{1}H NMR (400 MHz, $CDCl_3$): δ 8.77 (d, *J* = 2.7 Hz, 1H), 8.11 (dd, *J* = 9.1, 2.7 Hz, 1H), 7.93 (dd, *J* = 7.8, 1.6 Hz, 1H), 7.44 (td, *J* = 7.7, 1.6 Hz, 1H), 7.07 (ddd, *J* = 16.7, 7.9, 1.6 Hz, 2H), 6.54 (d, *J* = 9.0 Hz, 1H); ^{13}C NMR (101 MHz, $CDCl_3$): δ 161.5, 154.5, 140.7, 135.8, 130.4, 127.9, 126.2, 125.4, 122.0, 114.5, 89.9, 85.9; HRMS (ESI): Calcd for $C_{12}H_7I_2NO_3$ $[M+Na]^+$: 489.8408; found: 489.8406

Synthesis of 2-(2-iodo-4-nitrophenoxy)phenyl acetate (9)

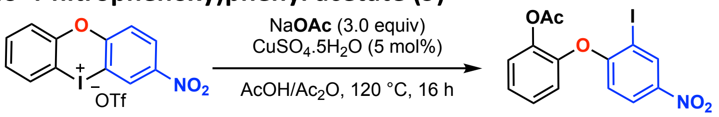

4a (1.0 equiv) **9** (86%)

Product **9** was synthesized following a procedure reported by Yu and co-workers.[15] Diaryliodonium salt **4a** (49 mg, 0.1 mmol, 1.0 equiv) was dissolved in AcOH (1.0 mL) and AcO_2 (0.2 mL) in a microwave vial. $CuSO_4{\cdot}5H_2O$ (2.5 mg, 10.0 µmol, 0.10 equiv) was added and the mixture was stirred at 120 °C for 20 hours. When the reaction time had elapsed, EtOAc (5 mL) and water (5 mL) were added and the phases were separated. The organic phase was washed with EtOAc (2 x 5 mL), dried over Na_2SO_4 and the solvent was concentrated *in vacuo*. The product was isolated by column chromatography, eluted with P:EtOAc 10:1 to give compound **9** as a colorless oil (35 mg, 0.086 mmol, 86%); R_f = 0.29 in P:EtOAc 10:1; ^{1}H NMR (400 MHz, $CDCl_3$): δ 8.73 (d, *J* = 2.7 Hz, 1H), 8.11 (dd, *J* = 9.1, 2.7 Hz, 1H), 7.36 – 7.30 (m, 2H), 7.27 – 7.22 (m, 1H), 7.19 – 7.14 (m, 1H), 6.76 (d, *J* = 9.1 Hz, 1H), 2.17 (s, 3H); ^{13}C NMR (101 MHz, $CDCl_3$): δ 168.6, 162.0, 146.1, 143.4, 142.4, 135.5, 127.5, 126.8, 125.5, 124.7, 122.2, 114.8, 85.3, 20.8; HRMS (ESI): Calcd for $C_{14}H_{10}INO_5$ $[M+Na]^+$: 421.9496; found: 421.9486

Synthesis of 2-azido-1-(2-iodophenoxy)-4-nitrobenzene (10)

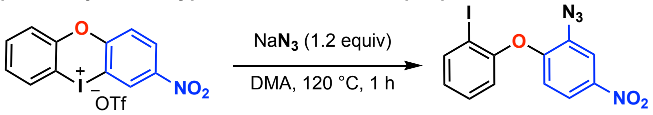

4a (1.0 equiv) **10** (31%)

Compound **10** was synthesized according to a modified literature procedure developed by Jiang and co-workers.[16] Diaryliodonium salt **4a** (50 mg, 0.10 mmol, 1.0 equiv) and NaN_3 (7.8 mg, 0.12 mmol, 1.2 equiv) were added to an oven-dried microwave vial. The atmosphere was exchanged to argon, after which DMA (1 mL) was added and the reaction mixture was stirred at 120 °C for 1 hour. After completion of the reaction time, water (5 mL) was added and the product was extracted with EtOAc. The combined organic layers were washed with brine (5 x 10 mL), dried over Na_2SO_4, and evaporated *in vacuo*. After column chromatography eluting with PE:EtOAc 10:1, the product **10** was isolated as a yellow solid (12 mg, 0.03 mmol, 31%). R_f = 0.25 in PE:EtOAc 10:1; ^{1}H NMR (400 MHz, $CDCl_3$): δ 7.99 (ddd, J = 8.8, 2.5, 0.7 Hz, 1H), 7.92 (dd, J = 8.0, 1.5 Hz, 1H), 7.53 (d, J = 2.5 Hz, 1H), 7.40 (ddt, J = 8.1, 7.4, 1.1 Hz, 1H), 7.22 (d, J = 9.5 Hz, 1H), 7.05 – 6.96 (m, 2H); ^{13}C NMR (101 MHz, $CDCl_3$): δ 154.4, 149.0, 144.8, 140.6, 137.2, 130.3, 127.3, 121.0, 120.1, 119.6, 113.0, 88.7; Note: product decomposes during HRMS.

Synthesis of 2-(2-iodo-4-nitrophenoxy)phenyl benzoate (12)

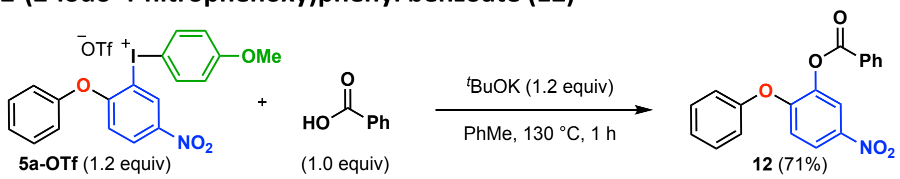

Product **12** was synthesized following a literature procedure from Olofsson and co-workers[17] To an oven-dried microwave vial, ^{t}BuOK (27 mg, 0.24 mmol, 1.2 equiv) was added and the vial was capped. The atmosphere was exchanged to argon before anhydrous toluene (1.2 mL) was added followed by benzoic acid (24 mg, 0.2 mmol, 1.0 equiv). The reaction was stirred at ambient temperature for 10 minutes before diaryliodonium salt **5a-OTf** (143 mg, 0.24 mmol, 1.2 equiv) was added in one portion. The reaction was transferred to a preheated oil bath set at 130 °C where it was stirred for 1 hour. When the reaction time had elapsed, Et_2O (5 mL) and water (5 mL) was added and the phases where separated. The organic phase was washed with Et_2O (2 x 5 mL), dried over Na_2SO_4 and the solvent was concentrated *in vacuo*. The reaction mixture was purified by column chromatography by dry-loading on silica, eluted with P:Et_2O, gradient: 95:5 to 90:10, to give compound **12** as a colorless oil (47 mg, 0.140 mmol, 71%); R_f = 0.27 in P:Et_2O 90:10; ^{1}H NMR (400 MHz, $CDCl_3$): δ 8.22 (d, *J* = 2.7 Hz, 1H), 8.15 (dd, *J* = 8.4, 1.4 Hz, 2H), 8.10 (dd, *J* = 9.1, 2.7 Hz, 1H), 7.67 – 7.62 (m, 1H), 7.50 (t, *J* = 8.1 Hz, 2H), 7.42 – 7.35 (m, 2H), 7.24 – 7.18 (m, 1H), 7.12 – 7.06 (m, 2H), 6.97 (d, *J* = 9.1 Hz, 1H); ^{13}C NMR (101 MHz, $CDCl_3$): δ 155.7, 154.9, 142.7, 140.9, 134.2, 130.6, 130.3, 128.8, 128.4, 125.6, 122.9 120.3, 120.3, 117.5, 114.7; HRMS (ESI): Calcd for $C_{19}H_{13}NO_5$ $[M+Na]^+$: 358.0686; found: 358.0675.

Synthesis of 2-iodo-4-nitro-1-(2-phenoxyphenoxy)benzene (13)

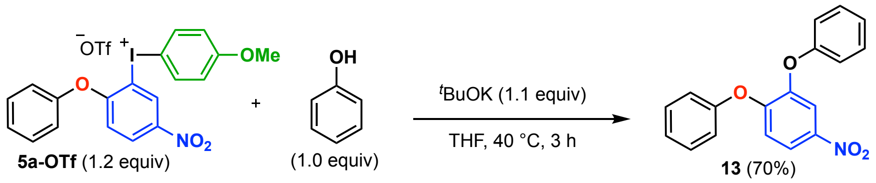

Product **13** was synthesized following a literature procedure from Olofsson and co-workers.[18] ^{t}BuOK (15 mg, 0.132 mmol, 1.1 equiv) was added to an oven-dried microwave vial. The vial was placed in an ice bath, and THF (1.2 mL) was added. The vial was capped and the reaction was stirred at 0 °C for 15 minutes before diaryliodonium salt **5a-OTf** (89 mg, 0.144 mmol, 1.2 equiv) was added in one portion under open air. The vial was transferred to a pre-heated oil bath where it was stirred at 40 °C for 3 hours. When the reaction time had elapsed, Et_2O (5 mL) and water (5 mL) was added and the phases where separated. The organic phase was washed with Et_2O (2 x 5 mL), dried over Na_2SO_4 and the solvent was concentrated *in vacuo*. The reaction mixture was purified by column chromatography. The crude was dry-loaded on silica and then eluted with P:Et_2O, 97:2, to give compound **13** as a colorless oil (26 mg, 0.085 mmol, 70%); R_f = 0.23 in P:Et_2O 97:3; ^{1}H NMR (400 MHz, $CDCl_3$): δ 7.94 (dd, *J* = 9.0, 2.7 Hz, 1H), 7.88 (d, *J* = 2.7 Hz, 1H), 7.43 – 7.34 (m, 4H), 7.19 (dt, *J* = 14.7, 7.4 Hz, 2H), 7.08 – 7.01 (m, 4H), 6.98 (d, *J* = 9.0 Hz, 1H); ^{13}C NMR (101 MHz, $CDCl_3$): δ 156.2, 155.3, 154.4, 147.4, 143.2, 130.3, 130.2, 125.2, 124.5, 120.0, 119.8 118.6, 118.2, 115.9; HRMS (ESI): Calcd for $C_{18}H_{13}INO_4$ $[M+Na]^+$: 330.0737; found: 330.0733.

Synthesis of 4-nitro-2-(1-nitrocyclopentyl)-1-phenoxybenzene (14)

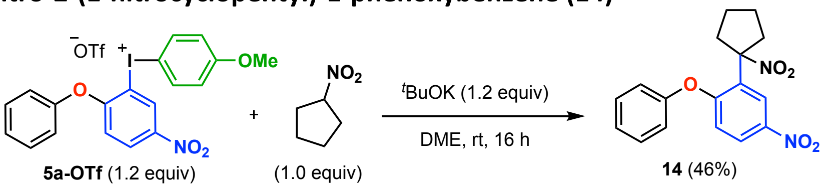

Product **14** was synthesized following a literature procedure from Olofsson and co-workers.[19] ^{t}BuOK (13 mg, 0.12 mmol, 1.2 equiv) was dissolved in anhydrous DME (0.75 mL) in an oven-dried microwave

vial. The vial was capped and placed in an ice bath before nitrocyclopentane (0.011 mL, 0.1 mmol, 1.0 equiv) was added with a micro-syringe. The reaction was stirred at room temperature for 15 min before diaryliodonium salt **5a-OTf** (60 mg, 0.1 mmol, 1.2 equiv) was added in one portion under open air. The vial was capped again and anhydrous DME (0.25 mL) was added. The reaction was stirred for 16 hours at room temperature. The reaction mixture was purified by column chromatography without prior work up by dry-loading on silica, eluted with P:Et_2O gradient 98:2 to 90:10, to give compound **14** as a yellow solid (15 mg, 0.046 mmol, 46%); R_f = 0.1 in P:Et_2O 98:2; ^{1}H NMR (400 MHz, $CDCl_3$): δ 8.44 (d, *J* = 2.7 Hz, 1H), 8.16 (dd, *J* = 9.1, 2.7 Hz, 1H), 7.42 (dd, *J* = 8.5, 7.4 Hz, 2H), 7.31 – 7.22 (m, 1H), 7.00 (d, *J* = 7.5 Hz, 2H), 6.81 (d, *J* = 9.1 Hz, 1H), 3.09 – 2.98 (m, 2H), 2.49 – 2.37 (m, 2H), 2.11 – 1.99 (m, 2H), 1.99 – 1.87 (m, 2H); ^{13}C NMR (101 MHz, $CDCl_3$): δ 161.5, 154.1, 130.6, 130.5, 126.2, 126.1, 123.7, 120.7, 116.3, 98.5, 37.9, 24.0; HRMS (ESI): Calcd for $C_{17}H_{16}N_2O_5$ $[M+Na]^+$: 351.0943; found: 351.0951.

Synthesis of 2-(2-iodo-4-nitrophenoxy)phenyl (*Z*)-*N*-benzylbenzimidothioate (15)

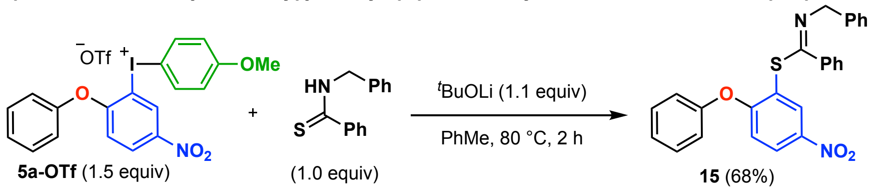

Product **15** was synthesized following a literature procedure from Olofsson and co-workers.[20] Diaryliodonium salt **5a-OTf** (90 mg, 0.15 mmol, 1.5 equiv), N-benzyl-thiobenzamide (23 mg, 0.1 mmol, 1.0 equiv) and ^{t}BuOLi (9 mg, 0.11 mmol, 1.1 equiv) were added to a microwave vial. The vial was capped and the atmosphere was exchanged to argon before anhydrous and degassed toluene (0.5 mL) was added. The reaction was stirred at 80 °C for 2 hours. The reaction mixture was purified by column chromatography without prior work up by dry-loading on silica, eluted with P:Et_2O 85:15, to give compound **15** as a colorless oil (30 mg, 0.068 mmol, 68%); R_f = 0.24 in P:Et_2O 85:15; ^{1}H NMR (400 MHz, $CDCl_3$): δ 8.33 (d, *J* = 2.8 Hz, 1H), 7.91 (dd, *J* = 9.1, 2.8 Hz, 1H), 7.64 (dd, *J* = 8.1, 1.7 Hz, 2H), 7.45 – 7.37 (m, 4H), 7.35 – 7.26 (m, 4H), 7.26 – 7.19 (m, 3H), 6.85 (d, *J* = 7.4 Hz, 2H), 6.58 (d, *J* = 9.1 Hz, 1H), 5.07 (s, 2H); ^{13}C NMR (101 MHz, $CDCl_3$): δ 161.9, 154.25, 142.3, 130.6, 130.5, 130.3, 130.2, 129.2, 128.8, 128.6, 128.5, 128.2, 128.1, 127.1, 125.9, 125.4, 120.5, 115.6, 59.0; HRMS (ESI): Calcd for $C_{26}H_{20}N_2O_3S$ $[M+H]^+$: 441.1267; found: 441.1263.

Synthesis of 2-((5-nitro-2-phenoxyphenyl)thio)benzo[*d*]thiazole (16)

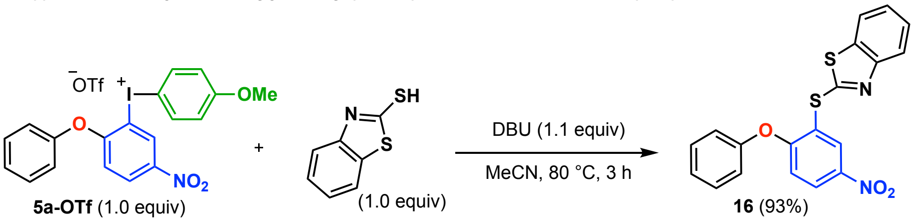

Product **16** was synthesized following a literature procedure from Kalek and co-workers.[21] Diaryliodonium salt **5a-OTf** (66 mg, 0.11 mmol, 1.1 equiv) and 2-mercaptobenzothiazole (17 mg, 0.1 mmol, 1.0 equiv) were added to an oven-dried microwave vial. The vial was capped and the atmosphere exchanged to argon before anhydrous MeCN (1.0 mL) was added. DBU (0.016 mL, 0.11 mmol, 1.1 equiv) was added with a micro-syringe and the reaction was stirred at 80 °C for 3 hours. The reaction mixture was purified by column chromatography without prior work up by dry-loading on silica, eluted with P:Et_2O 90:10, to give compound **16** as a colorless oil (36 mg, 0.093 mmol, 93%); R_f = 0.23 in P:Et_2O 90:10; ^{1}H NMR (400 MHz, $CDCl_3$): δ 8.69 (d, *J* = 2.8 Hz, 1H), 8.22 (dd, *J* = 9.2, 2.8 Hz, 1H), 7.91 (d, *J* = 8.1 Hz, 1H), 7.75 (dd, *J* = 8.1, 1.3 Hz, 1H), 7.44 (ddd, *J* = 8.3, 7.3, 1.3 Hz, 1H), 7.42 – 7.36 (m, 2H), 7.34 (ddd, *J* = 8.3, 7.3, 1.2 Hz, 1H), 7.26 – 7.21 (m, 1H), 7.05 (d, *J* = 7.5 Hz, 2H), 6.89 (d, *J* = 9.2 Hz, 1H); ^{13}C NMR (101 MHz, $CDCl_3$): δ 164.6, 163.4, 154.4, 153.7, 142.7, 135.9, 131.8, 130.5, 127.3, 126.6, 126.1, 125.1, 122.5, 121.4, 121.2, 120.8, 116.2; HRMS (ESI): Calcd for $C_{19}H_{12}N_2O_3S_2$ $[M+Na]^+$: 403.0187; found: 403.0182.

Synthesis of 2,4-dinitro-1-phenoxybenzene (17)

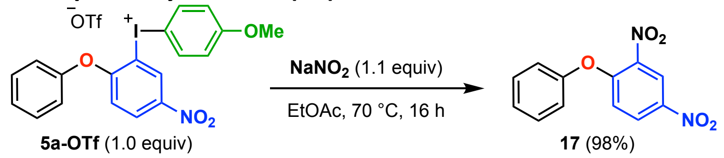

Product **17** was synthesized following a literature procedure from Olofsson and co-workers.[22] Diaryliodonium salt **5a-OTf** (60 mg, 0.1 mmol, 1.0 equiv) and $NaNO_2$ (8 mg, 0.11 mmol, 1.1 equiv) were added to a microwave vial. EtOAc (1 mL) was added before the vial was capped and the reaction was stirred at 70 °C for 16 hours. The reaction mixture was purified by column chromatography without prior work up by dry-loading on silica, eluted with P:Et_2O 98:2, to give compound **17** as a yellow solid (26 mg, 0.098 mmol, 98%); R_f = 0.20 in P:Et_2O 98:2; ^1H NMR (400 MHz, $CDCl_3$): δ 8.84 (d, *J* = 2.8 Hz, 1H), 8.31 (dd, *J* = 9.3, 2.8 Hz, 1H), 7.49 (t, *J* = 7.8 Hz, 2H), 7.34 (t, *J* = 7.4 Hz, 1H), 7.15 (d, *J* = 7.9 Hz, 2H), 7.03 (d, *J* = 9.3 Hz, 1H); ^{13}C NMR (101 MHz, $CDCl_3$): δ 156.4, 153.8, 141.5, 139.6, 130.9, 128.9, 126.8, 122.2, 120.7, 118.5; The spectral data is in agreement with literature.[23]

References:

[1] A. I. Vogel, B. S. Furniss, A. J. Hannaford, P. W. Smith, A. R. Tatchell, *Vogel's Textbook of Practical Organic Chemistry*, 5th ed., Prentice Hall, Harlow, **1996**.
[2] H. E. Gottlieb, V. Kotlyar, A. Nudelman, *J. Org. Chem.* **1997**, *62*, 7512-7515.
[3] T. Dohi, M. Ito, K. Morimoto, Y. Minamitsuji, N. Takenaga, Y. Kita, *Chem. Commun.* **2007**, 4152-4154.
[4] M. Damrath, L. D. Caspers, D. Duvinage, B. J. Nachtsheim, *Organic Letters* **2022**, *24*, 2562-2566.
[5] D. Zhu, Q. Liu, B. Luo, M. Chen, R. Pi, P. Huang, S. Wen, *Adv. Synth. Catal.* **2013**, *355*, 2172-2178.
[6] aB. Y. Karele, S. V. Kalnin, I. P. Grinberga, O. Y. Neiland, *Chemistry of Heterocyclic Compounds* **1973**, *9*, 226-229; bT. P. Tolstaya, L. D. Egorova, I. N. Lisichkina, *Chemistry of Heterocyclic Compounds* **1985**, *21*, 392-396.
[7] aM. Bielawski, M. Zhu, B. Olofsson, *Advanced Synthesis & Catalysis* **2007**, *349*, 2610-2618; bM. Bielawski, D. Aili, B. Olofsson, *The Journal of Organic Chemistry* **2008**, *73*, 4602-4607.
[8] E. Linde, D. Bulfield, G. Kervefors, N. Purkait, B. Olofsson, *Chem* **2022**, *8*, 850-865.
[9] N. Jalalian, T. B. Petersen, B. Olofsson, *Chemistry – A European Journal* **2012**, *18*, 14140-14149.
[10] N. Panda, I. Mattan, D. K. Nayak, *The Journal of Organic Chemistry* **2015**, *80*, 6590-6597.
[11] K. Mackey, D. J. Jones, L. M. Pardo, G. P. McGlacken, *Eur. J. Org. Chem.* **2021**, *2021*, 495-498.
[12] B. Luo, Q. Cui, H. Luo, Y. Hu, P. Huang, S. Wen, *Adv. Synth. Catal.* **2016**, *358*, 2733-2738.
[13] aŁ. Janczewski, A. Gajda, T. Gajda, *Eur. J. Org. Chem.* **2019**, *2019*, 2528-2532; bT.-T. Li, X.-H. Song, M.-S. Wang, N. Ma, *RSC Advances* **2014**, *4*, 40054-40060.
[14] B. Wu, N. Yoshikai, *Angew. Chem. Int. Ed.* **2015**, *54*, 8736-8739.
[15] aH. Xie, S. Yang, C. Zhang, M. Ding, M. Liu, J. Guo, F. Zhang, *J. Org. Chem.* **2017**, *82*, 5250-5262; bZ. Hu, Y. Tang, B. Yu, *J. Am. Chem. Soc.* **2019**, *141*, 4806-4810.
[16] M. Wang, Q. Fan, X. Jiang, *Org. Lett.* **2018**, *20*, 216-219.
[17] T. B. Petersen, R. Khan, B. Olofsson, *Organic Letters* **2011**, *13*, 3462-3465.
[18] N. Jalalian, E. E. Ishikawa, L. F. Silva, B. Olofsson, *Organic Letters* **2011**, *13*, 1552-1555.
[19] C. Dey, E. Lindstedt, B. Olofsson, *Org. Lett.* **2015**, *17*, 4554-4557.
[20] P. Villo, G. Kervefors, B. Olofsson, *Chemical Communications* **2018**, *54*, 8810-8813.
[21] S. Sarkar, N. Wojciechowska, A. A. Rajkiewicz, M. Kalek, *Eur. J. Org. Chem.* **2022**, e202101408.
[22] M. Reitti, P. Villo, B. Olofsson, *Angewandte Chemie International Edition* **2016**, *55*, 8928-8932.
[23] Bandna, N. R. Guha, A. K. Shil, D. Sharma, P. Das, *Tetrahedron Lett.* **2012**, *53*, 5318-5322.

NMR spectra

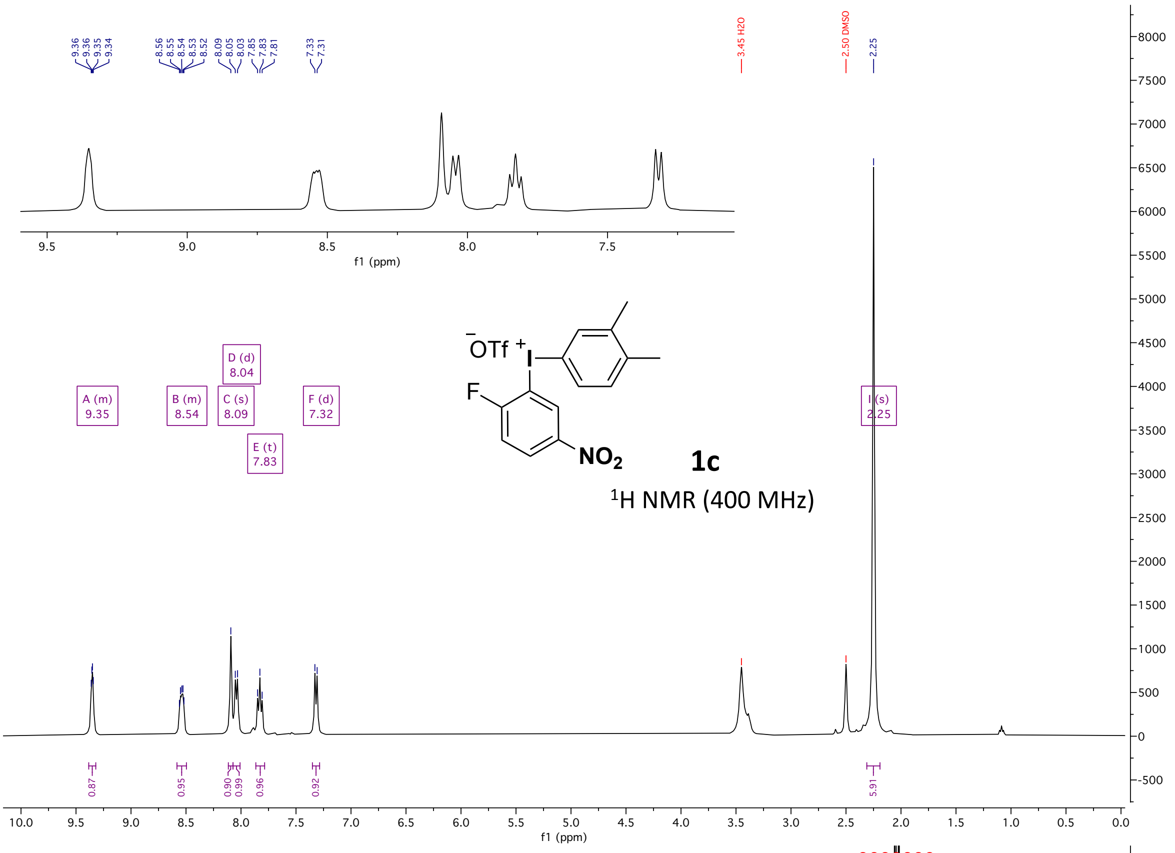
OTf
I
F
NO2
1c
1H NMR (400 MHz)
A (m) 9.35
B (m) 8.54
C (s) 8.09
D (d) 8.04
E (t) 7.83
F (d) 7.32
I (s) 2.25
3.45 H2O
2.50 DMSO
f1 (ppm)

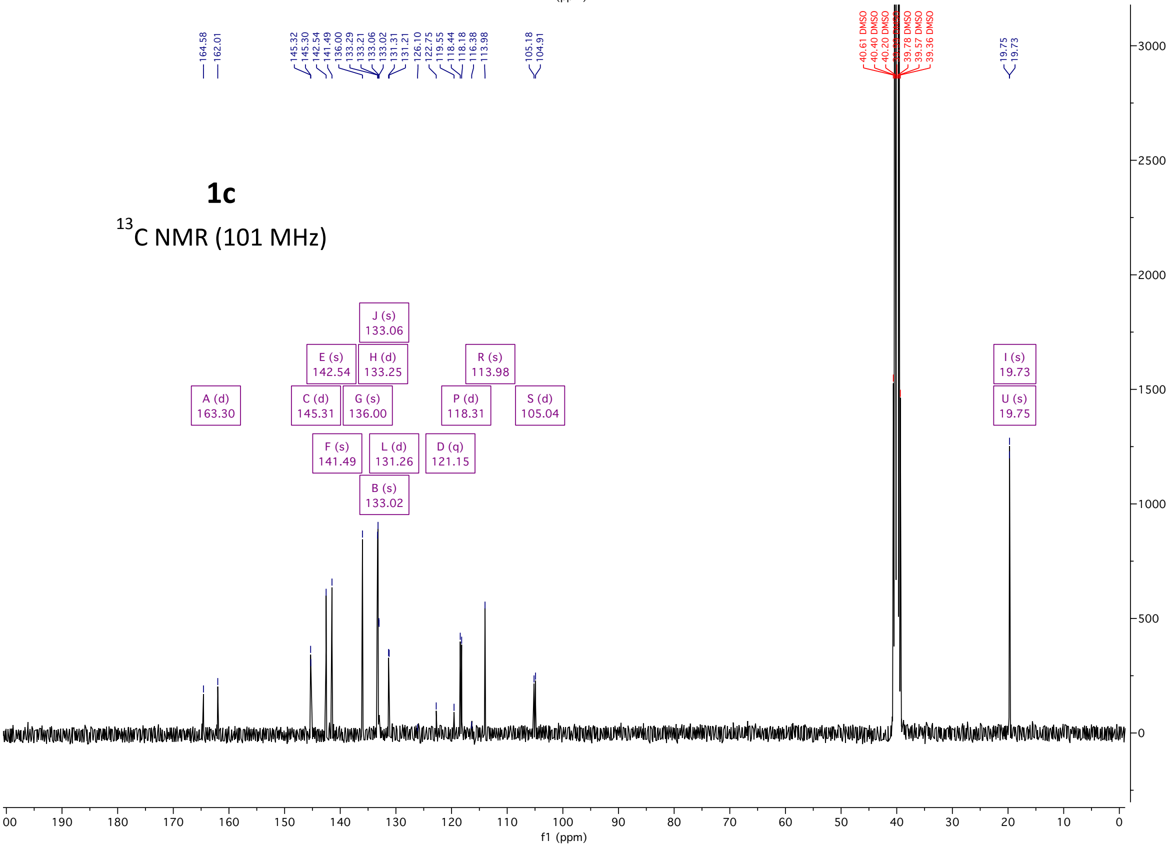
1c
13C NMR (101 MHz)
A (d) 163.30
C (d) 145.31
E (s) 142.54
F (s) 141.49
G (s) 136.00
H (d) 133.25
J (s) 133.06
B (s) 133.02
L (d) 131.26
D (q) 121.15
P (d) 118.31
R (s) 113.98
S (d) 105.04
I (s) 19.73
U (s) 19.75
f1 (ppm)

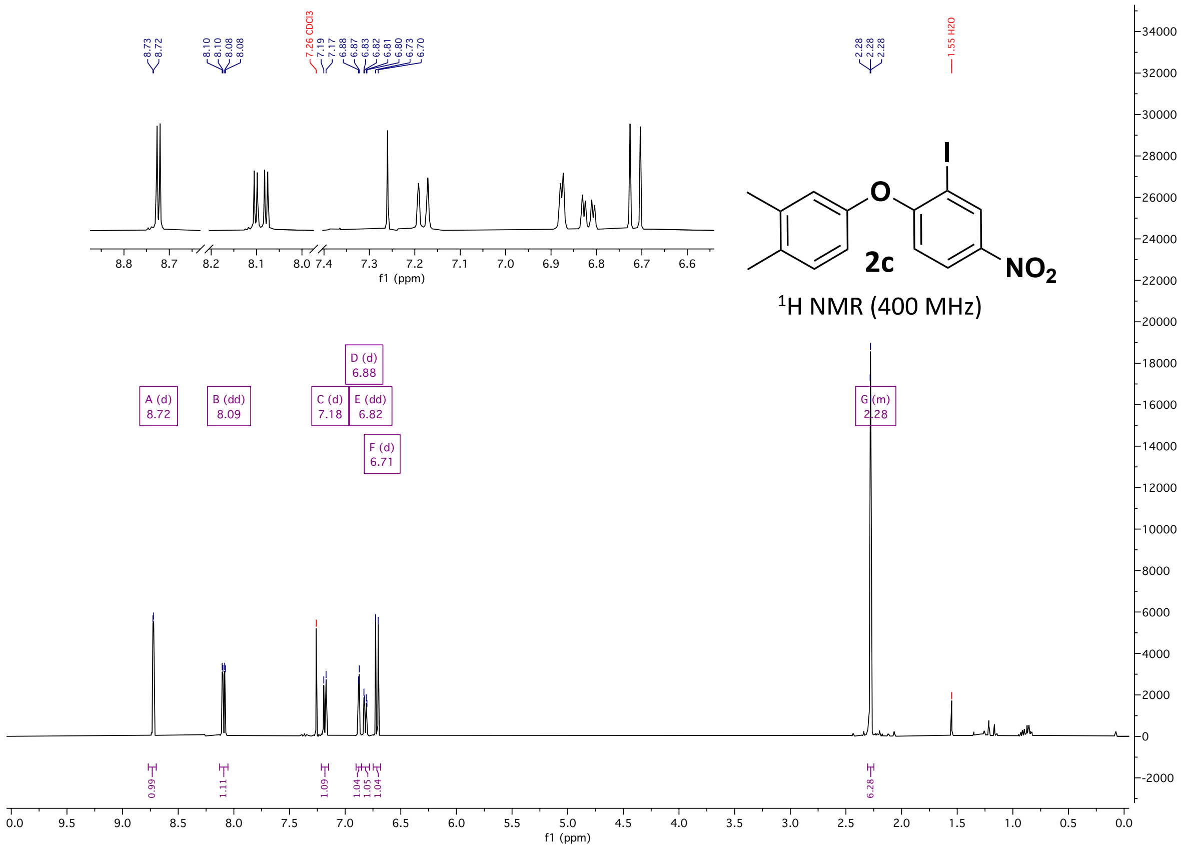

2c
^{1}H NMR (400 MHz)
A (d) 8.72
B (dd) 8.09
C (d) 7.18
D (d) 6.88
E (dd) 6.82
F (d) 6.71
G (m) 2.28
7.26 CDCl3
1.55 H2O
f1 (ppm)

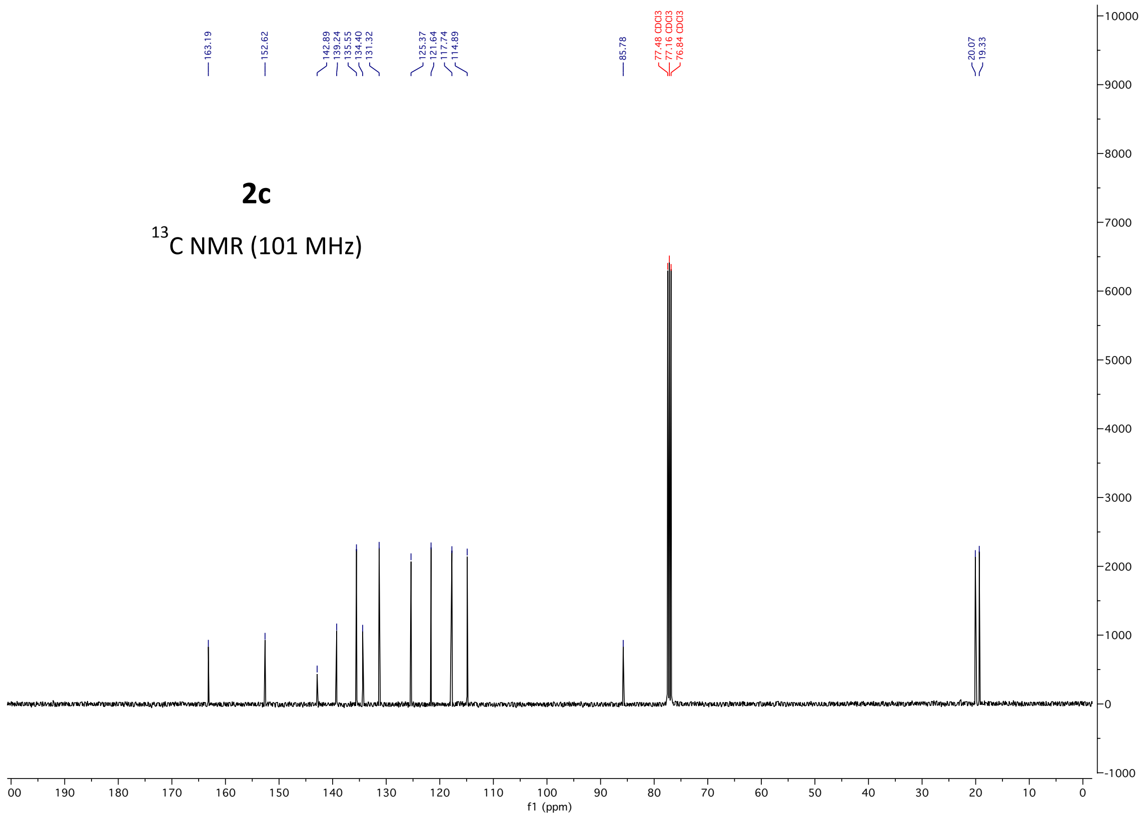

2c
^{13}C NMR (101 MHz)
163.19
152.62
142.89
139.24
135.55
134.40
131.32
125.37
121.64
117.74
114.89
85.78
77.48 CDCl3
77.16 CDCl3
76.84 CDCl3
20.07
19.33
f1 (ppm)

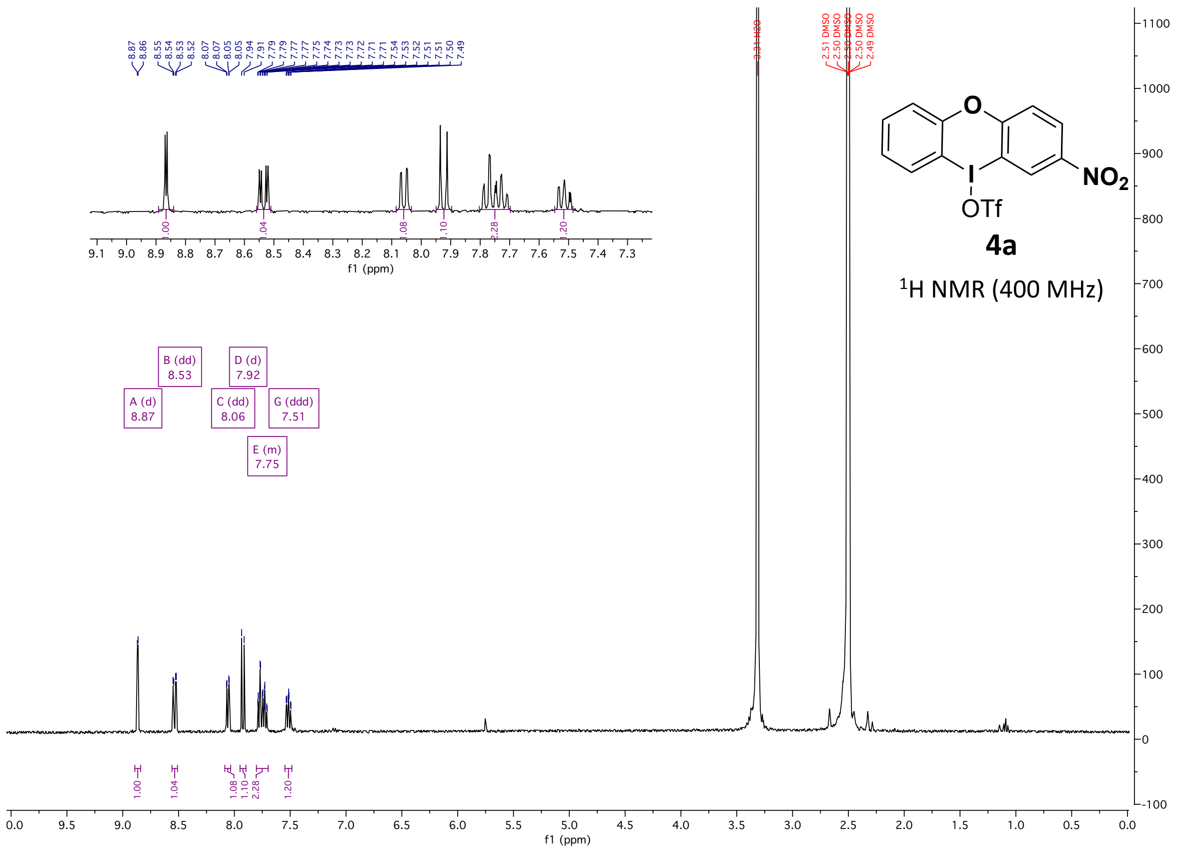

8.87
8.86
8.55
8.54
8.53
8.52
8.07
8.07
8.05
8.05
7.94
7.91
7.79
7.79
7.77
7.77
7.75
7.74
7.73
7.73
7.73
7.72
7.71
7.71
7.54
7.53
7.53
7.52
7.51
7.51
7.50
7.49
3.31 H2O
2.51 DMSO
2.50 DMSO
2.50 DMSO
2.50 DMSO
2.49 DMSO
A (d) 8.87
B (dd) 8.53
C (dd) 8.06
D (d) 7.92
E (m) 7.75
G (ddd) 7.51
1.00
1.04
1.08
1.10
2.28
1.20
f1 (ppm)
O
I
OTf
NO2
4a
1H NMR (400 MHz)

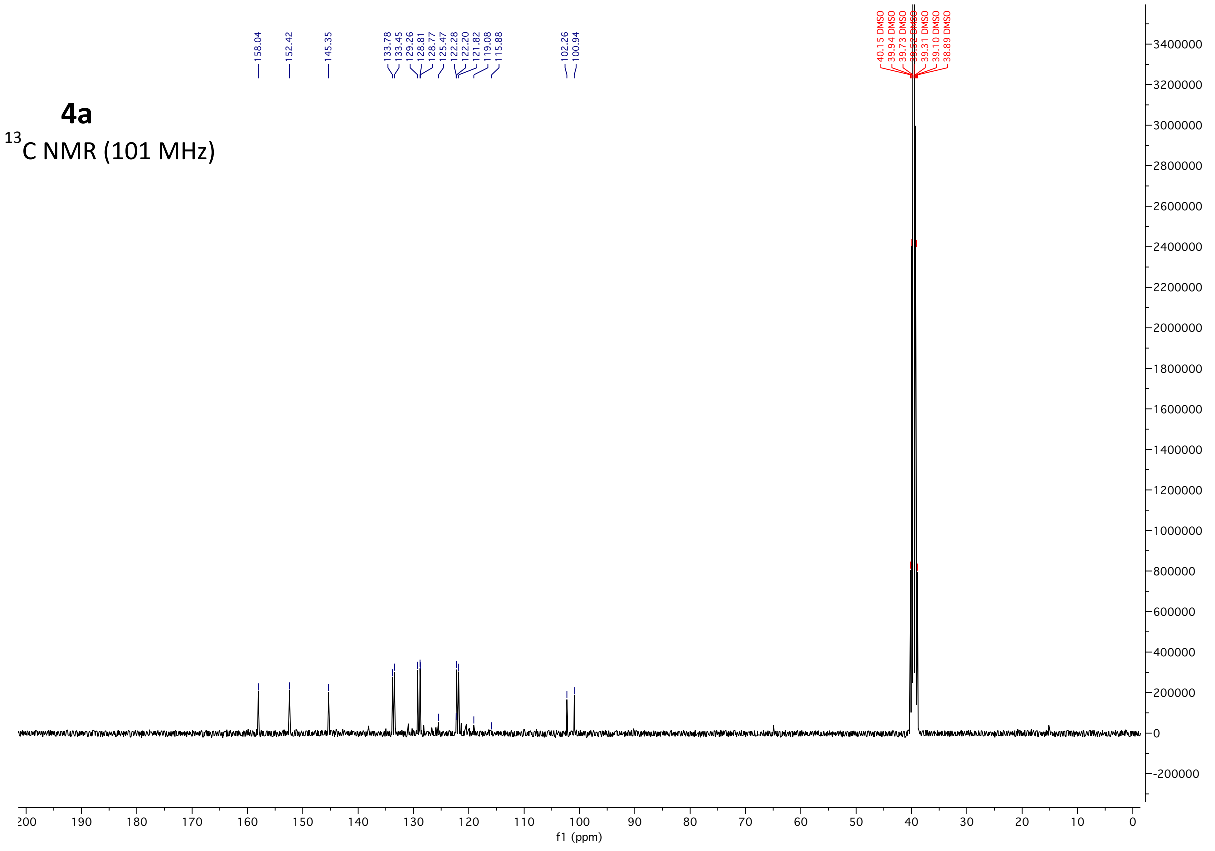

158.04
152.42
145.35
133.78
133.45
129.26
128.81
128.77
125.47
122.28
122.20
121.82
119.08
115.88
102.26
100.94
40.15 DMSO
39.94 DMSO
39.73 DMSO
39.31 DMSO
39.10 DMSO
38.89 DMSO
4a
13C NMR (101 MHz)
f1 (ppm)

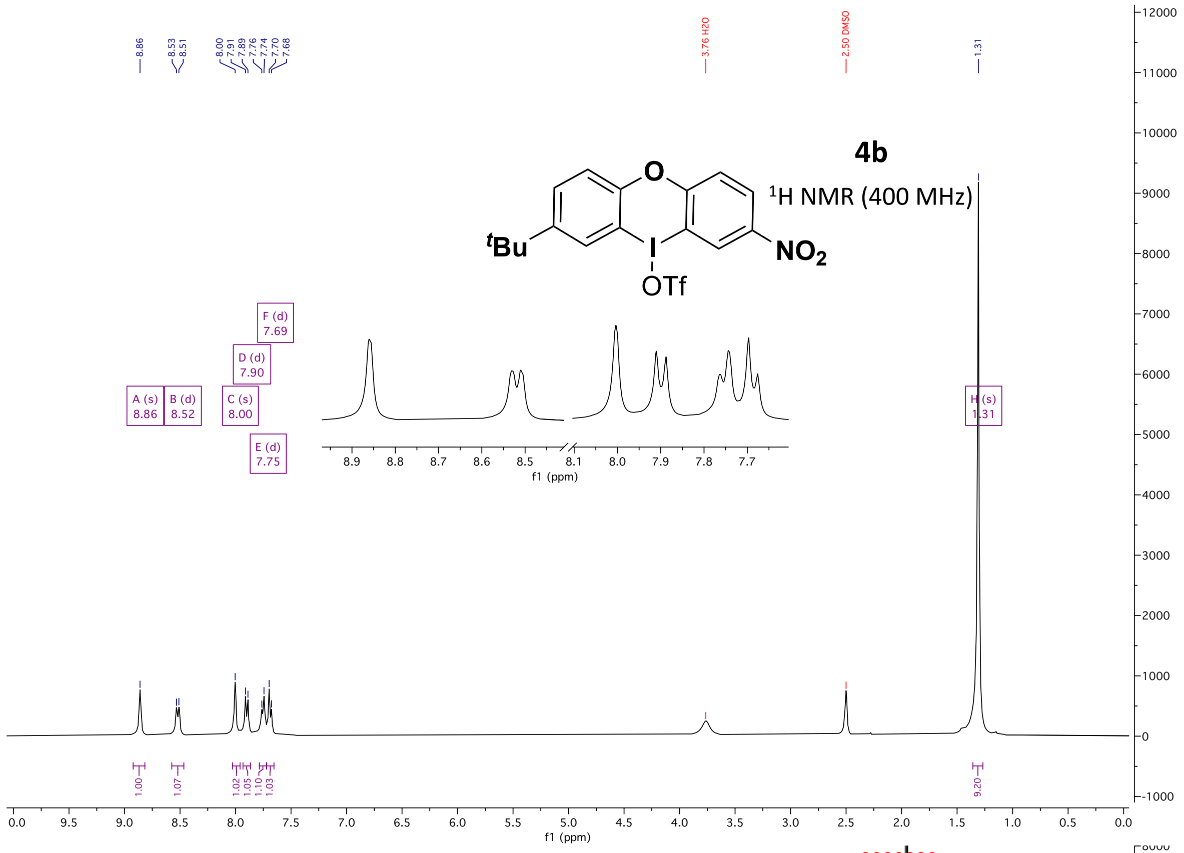
4b
^{1}H NMR (400 MHz)
tBu
NO2
OTf
f1 (ppm)

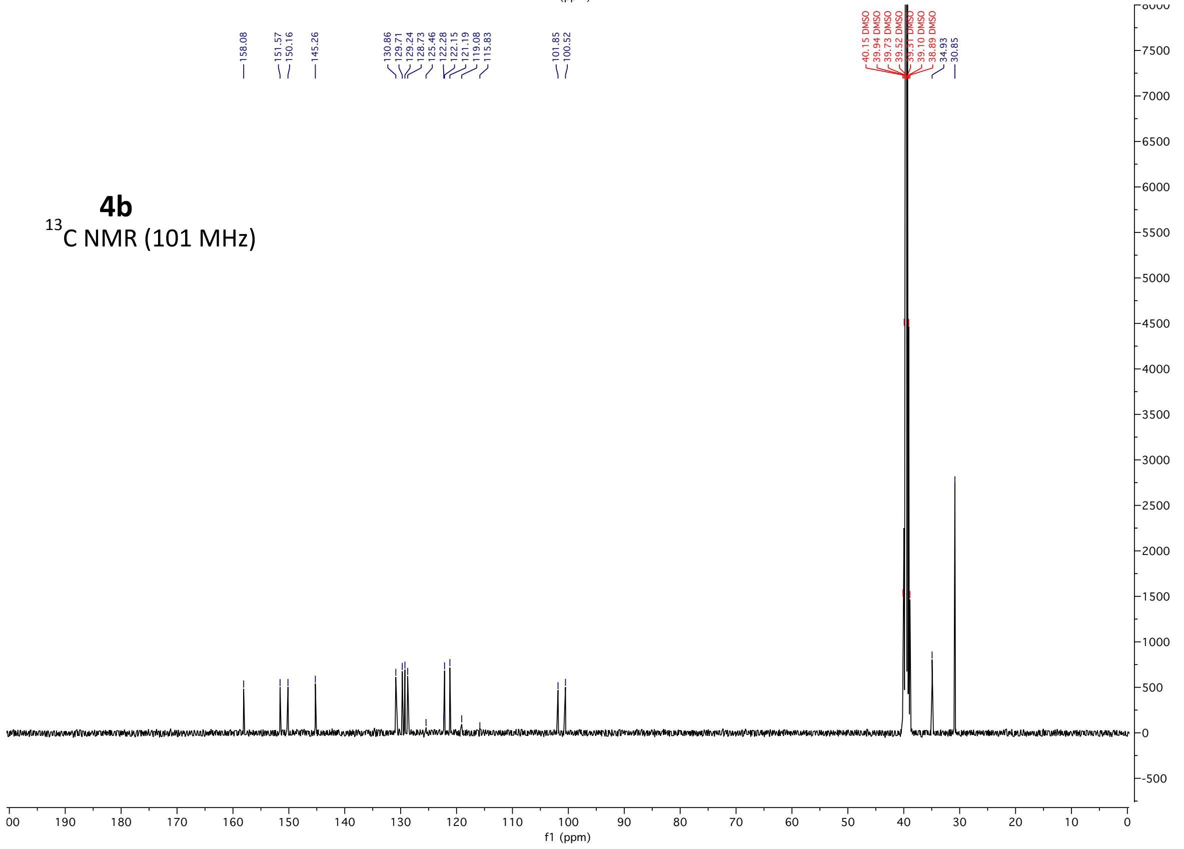
4b
^{13}C NMR (101 MHz)
f1 (ppm)

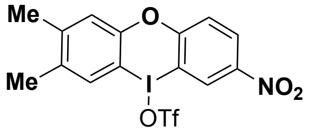

4c

^{1}H NMR (400 MHz)

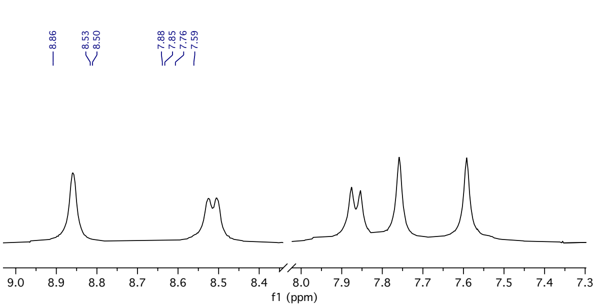

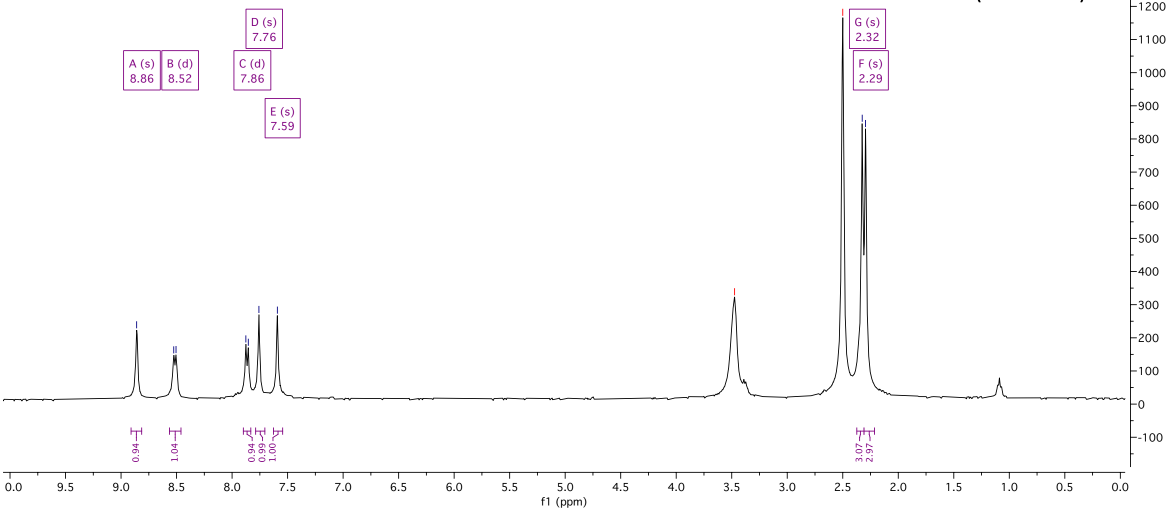

4c

^{13}C NMR (101 MHz)

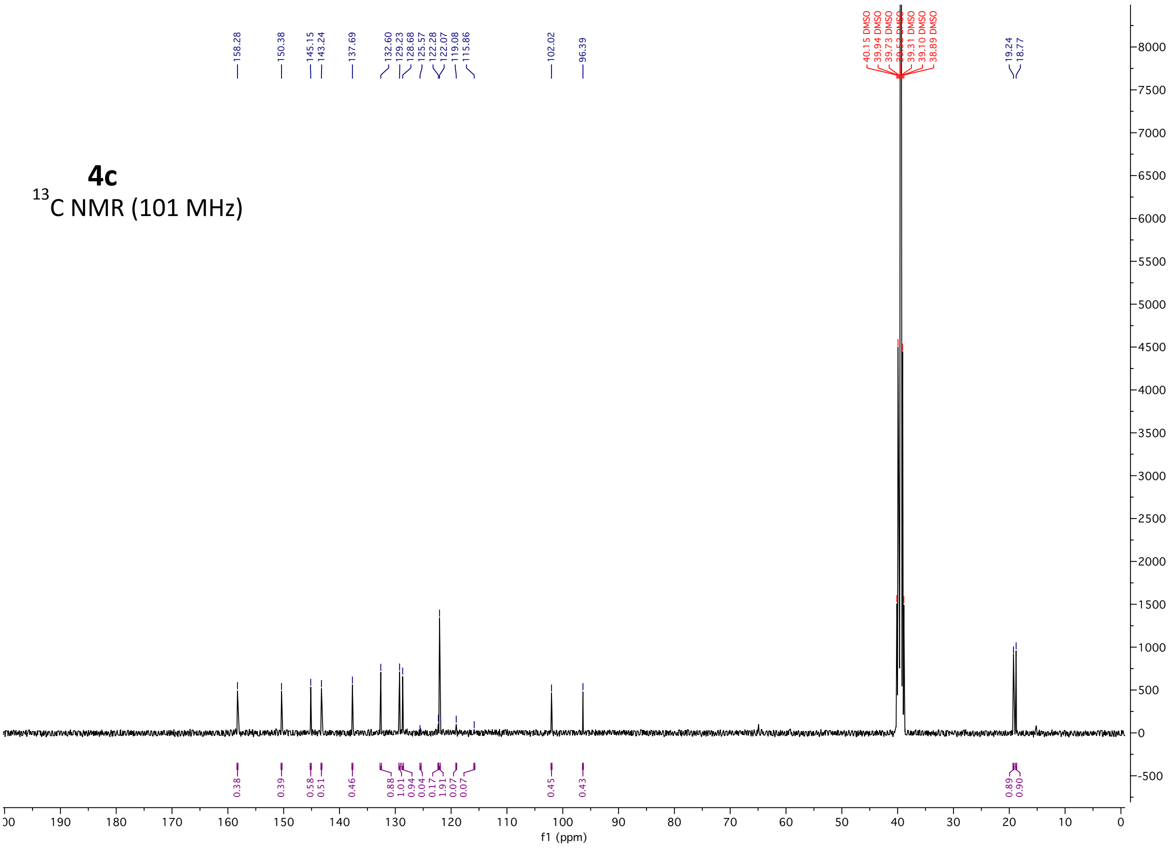

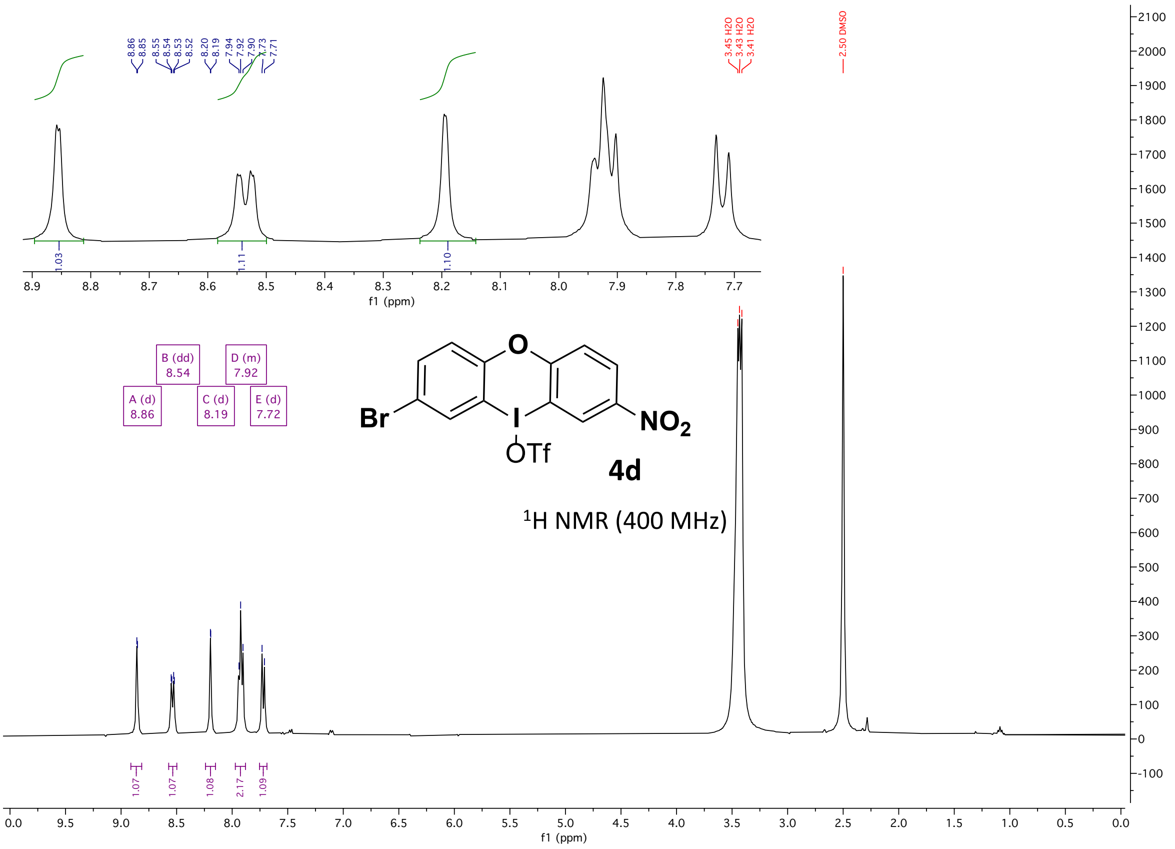
4d
¹H NMR (400 MHz)
A (d) 8.86
B (dd) 8.54
C (d) 8.19
D (m) 7.92
E (d) 7.72
f1 (ppm)

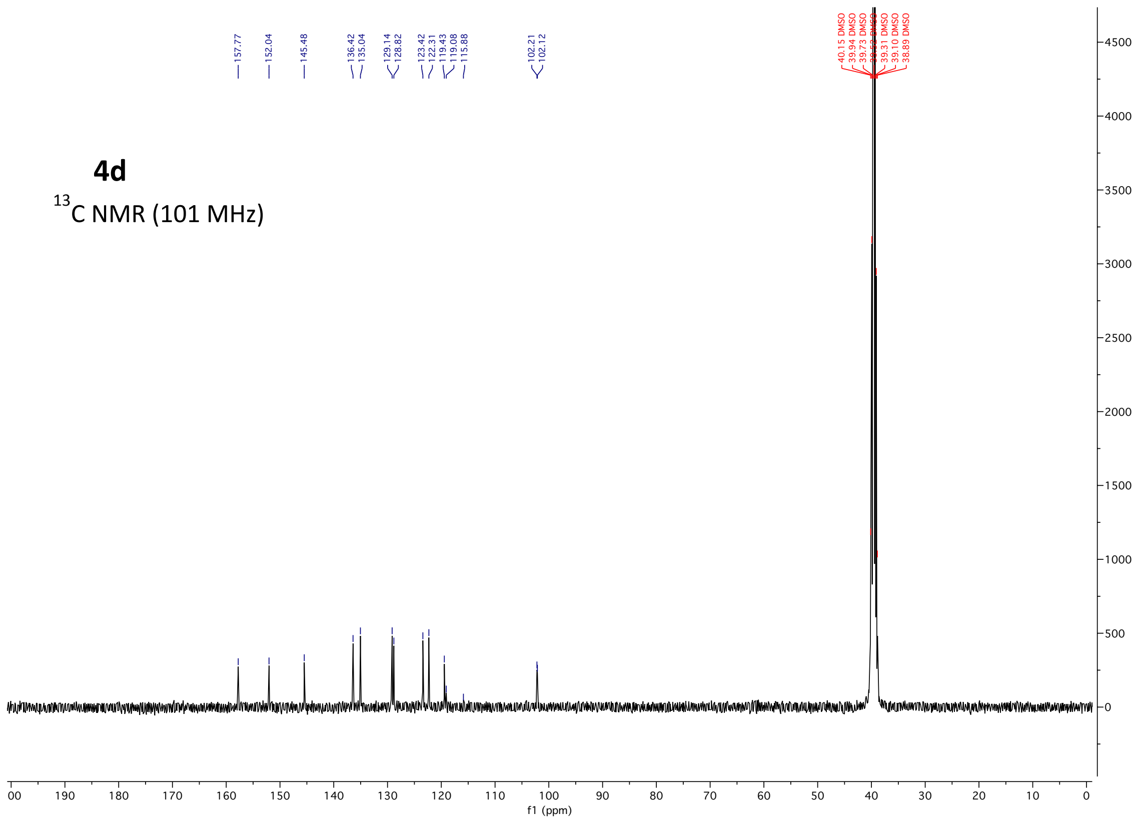
4d
¹³C NMR (101 MHz)
f1 (ppm)

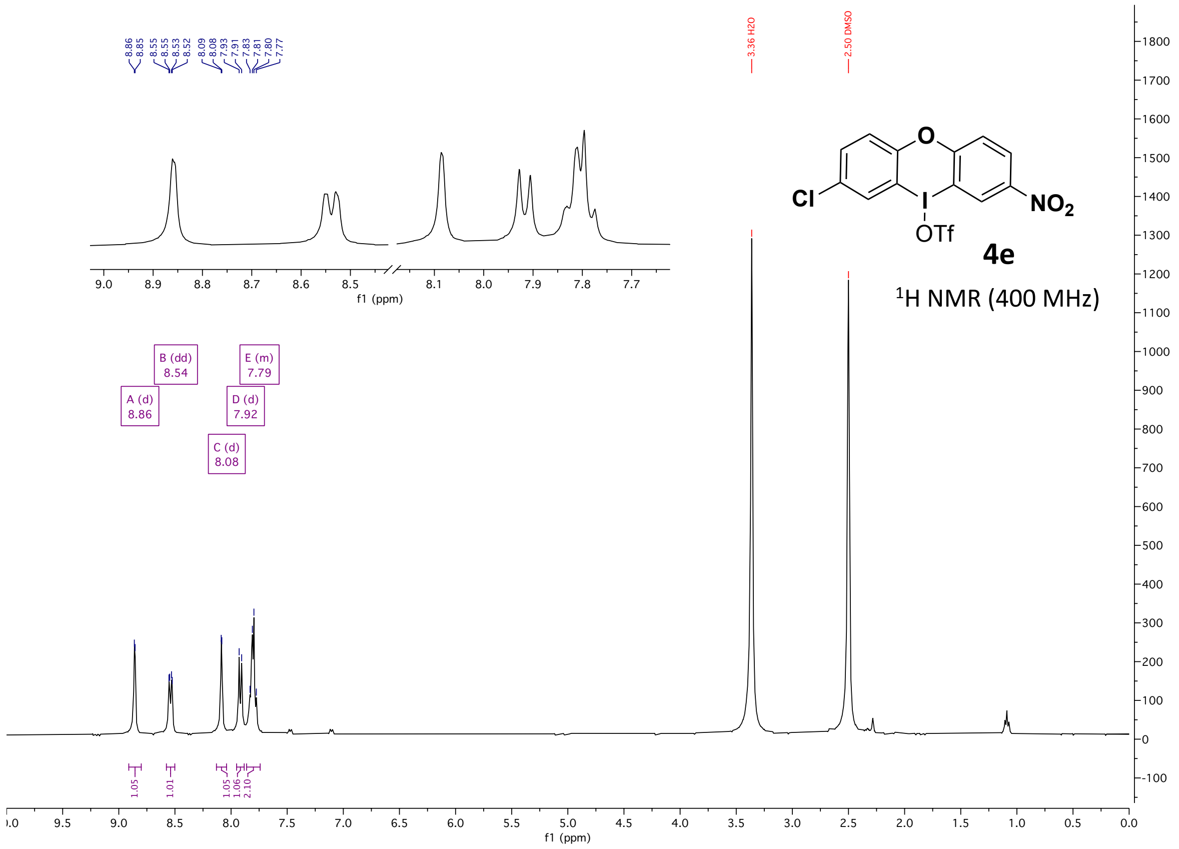

4e
1H NMR (400 MHz)
f1 (ppm)

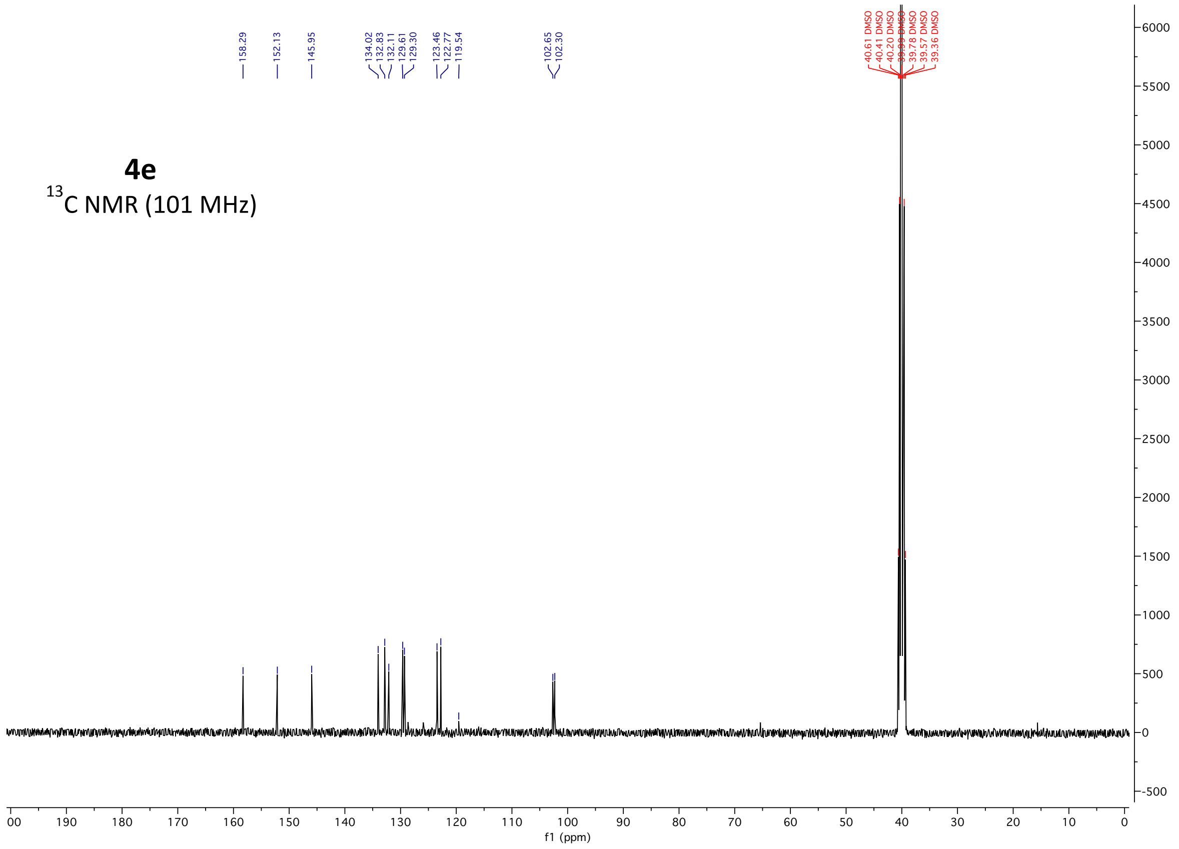

4e
13C NMR (101 MHz)
f1 (ppm)

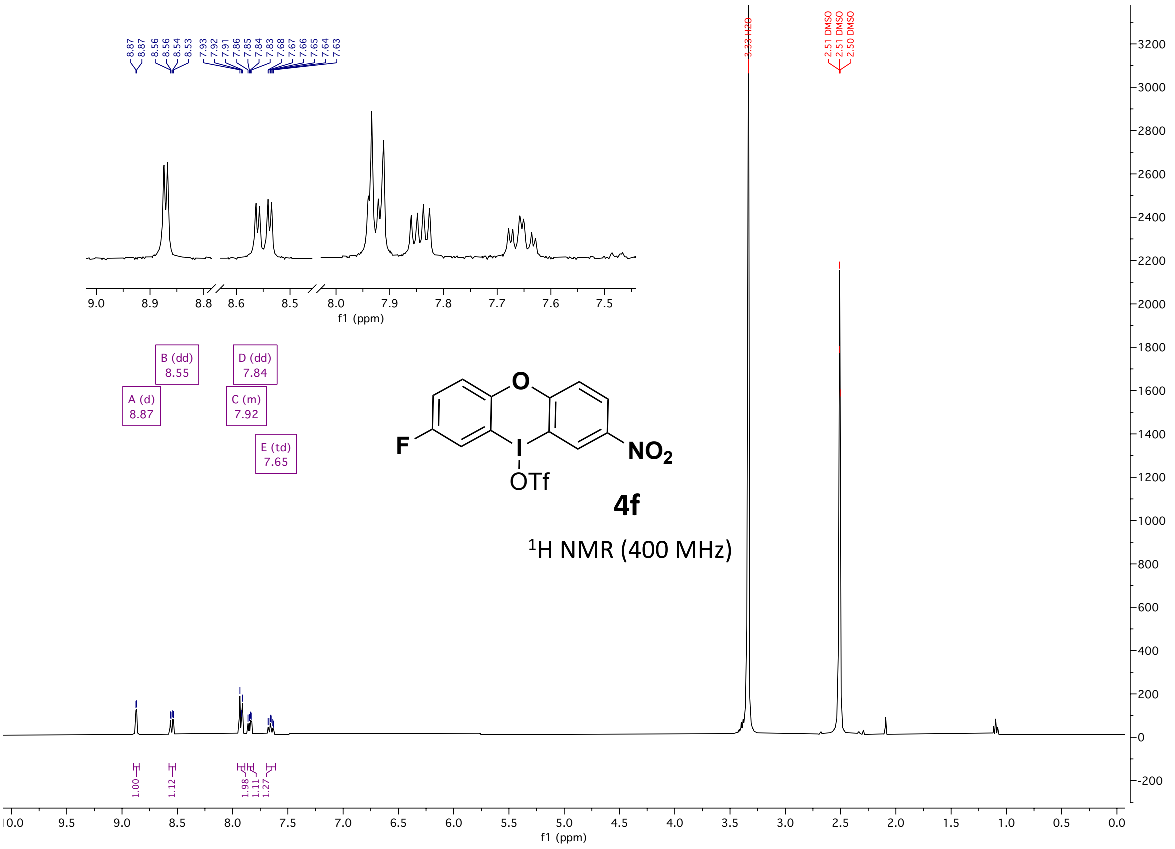
4f
^{1}H NMR (400 MHz)
A (d) 8.87
B (dd) 8.55
C (m) 7.92
D (dd) 7.84
E (td) 7.65
f1 (ppm)

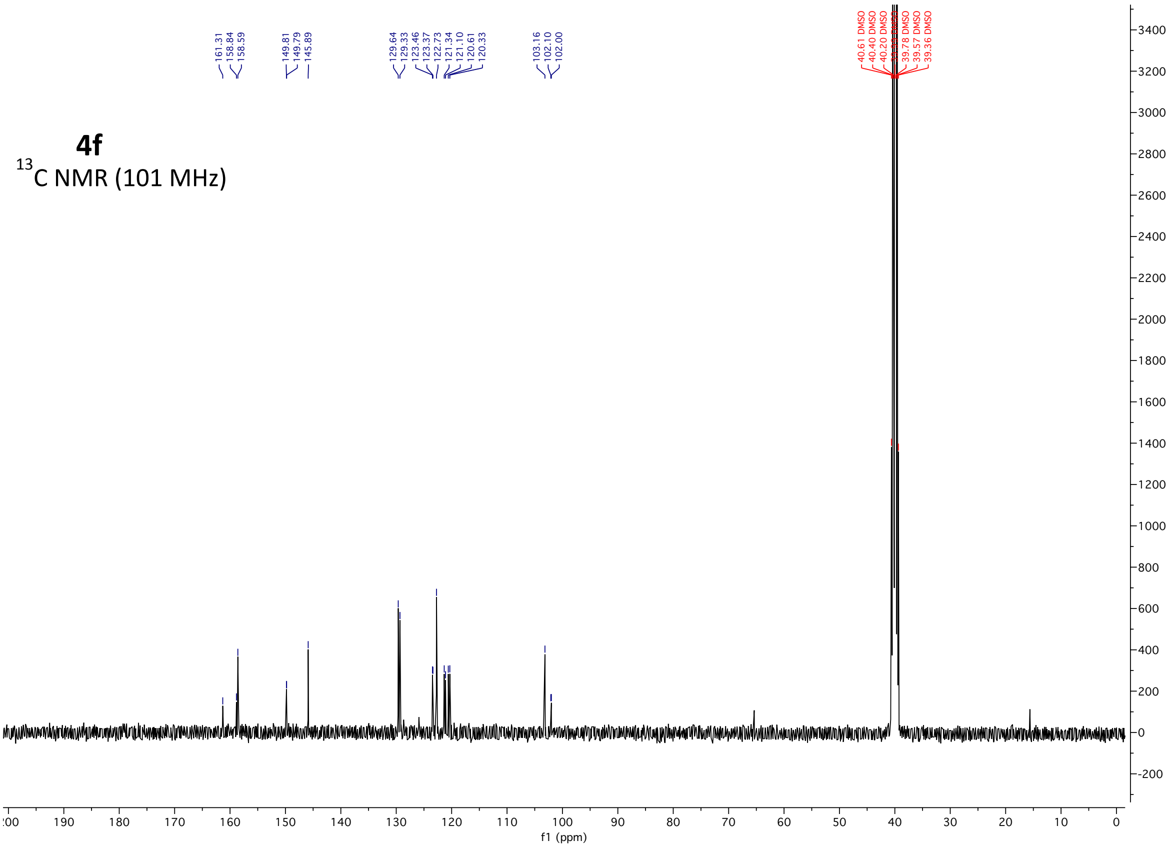
4f
^{13}C NMR (101 MHz)
f1 (ppm)

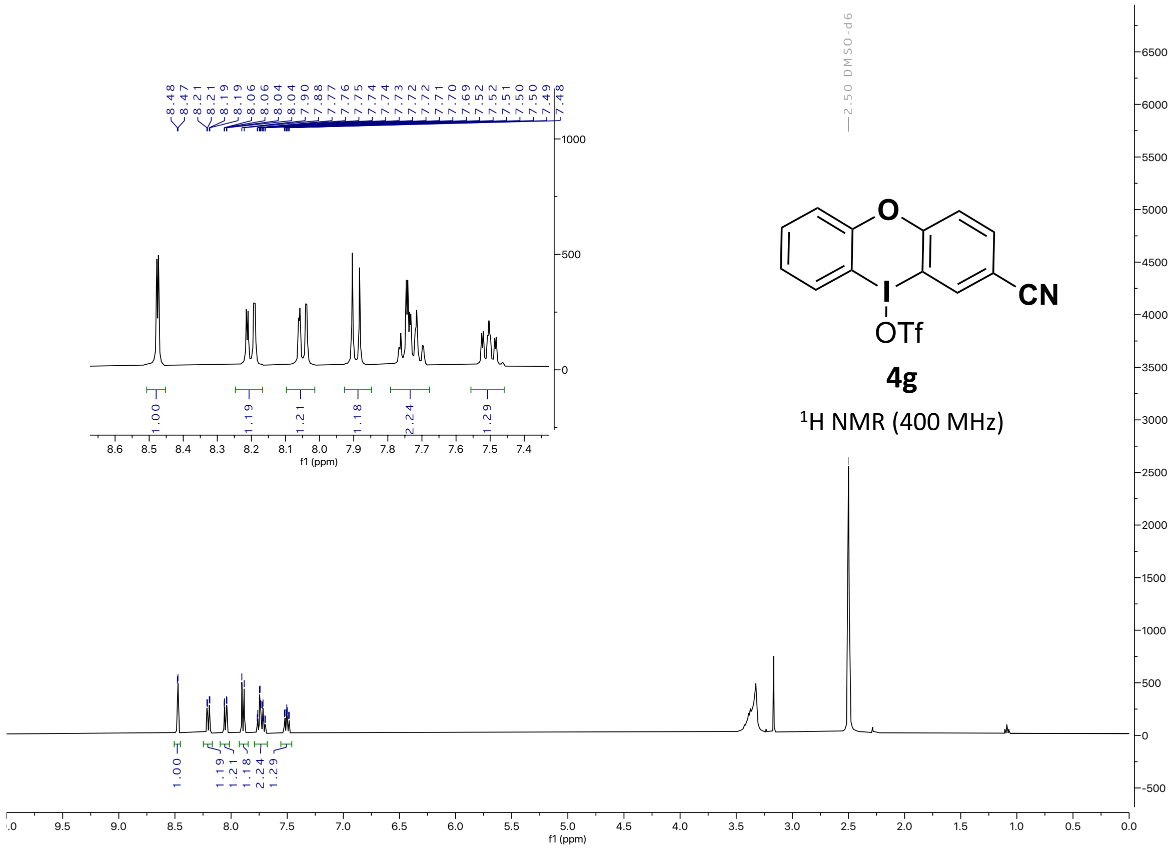

O
I
OTf
CN
4g
^{1}H NMR (400 MHz)
2.50 DMSO-d6
f1 (ppm)

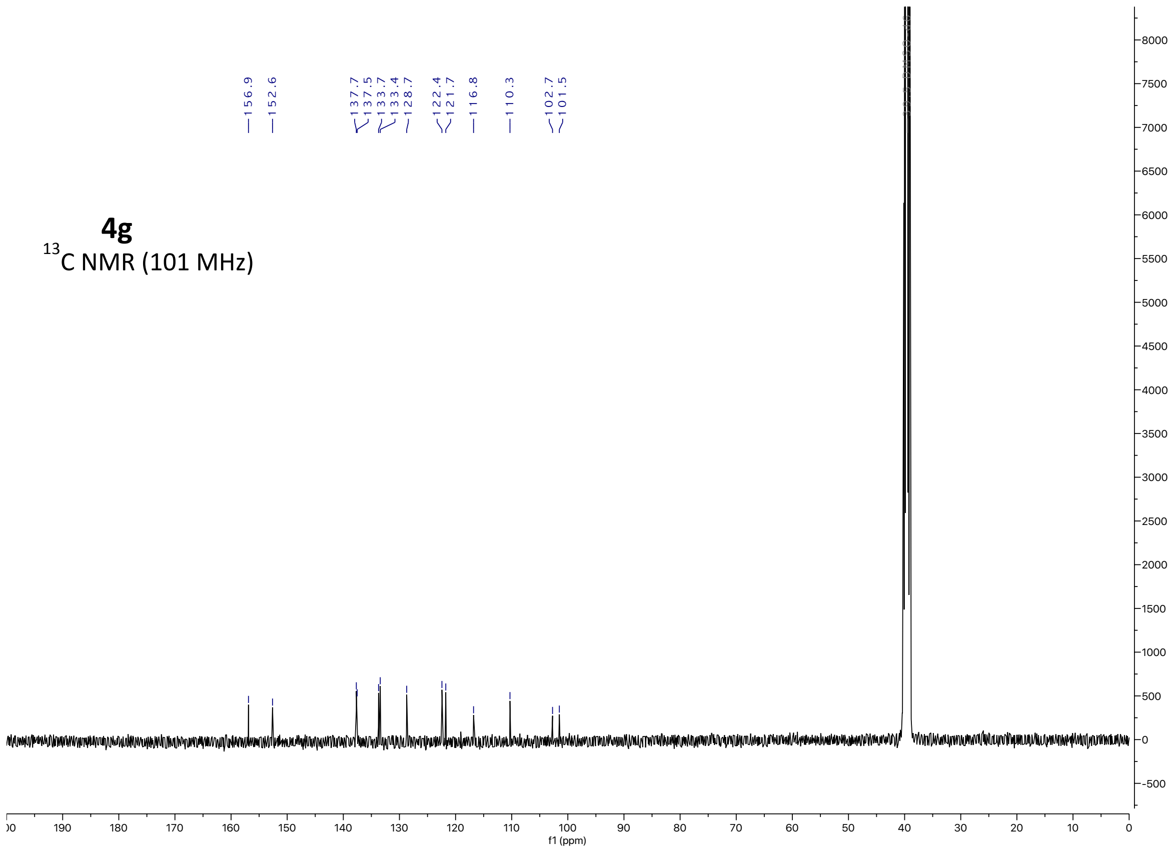

4g
^{13}C NMR (101 MHz)
156.9
152.6
137.7
137.5
133.7
133.4
128.7
122.4
121.7
116.8
110.3
102.7
101.5
f1 (ppm)

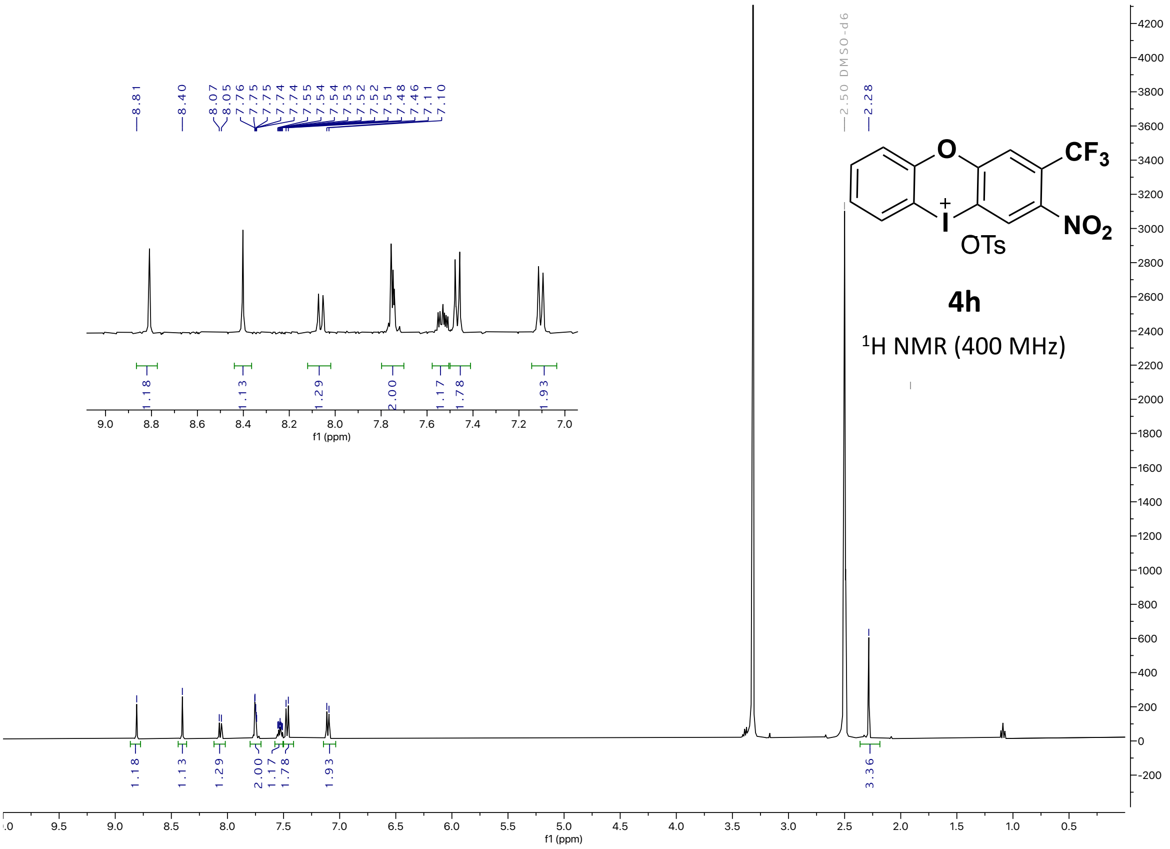

4h
^{1}H NMR (400 MHz)
OTs
CF3
NO2
2.50 DMSO-d6
f1 (ppm)

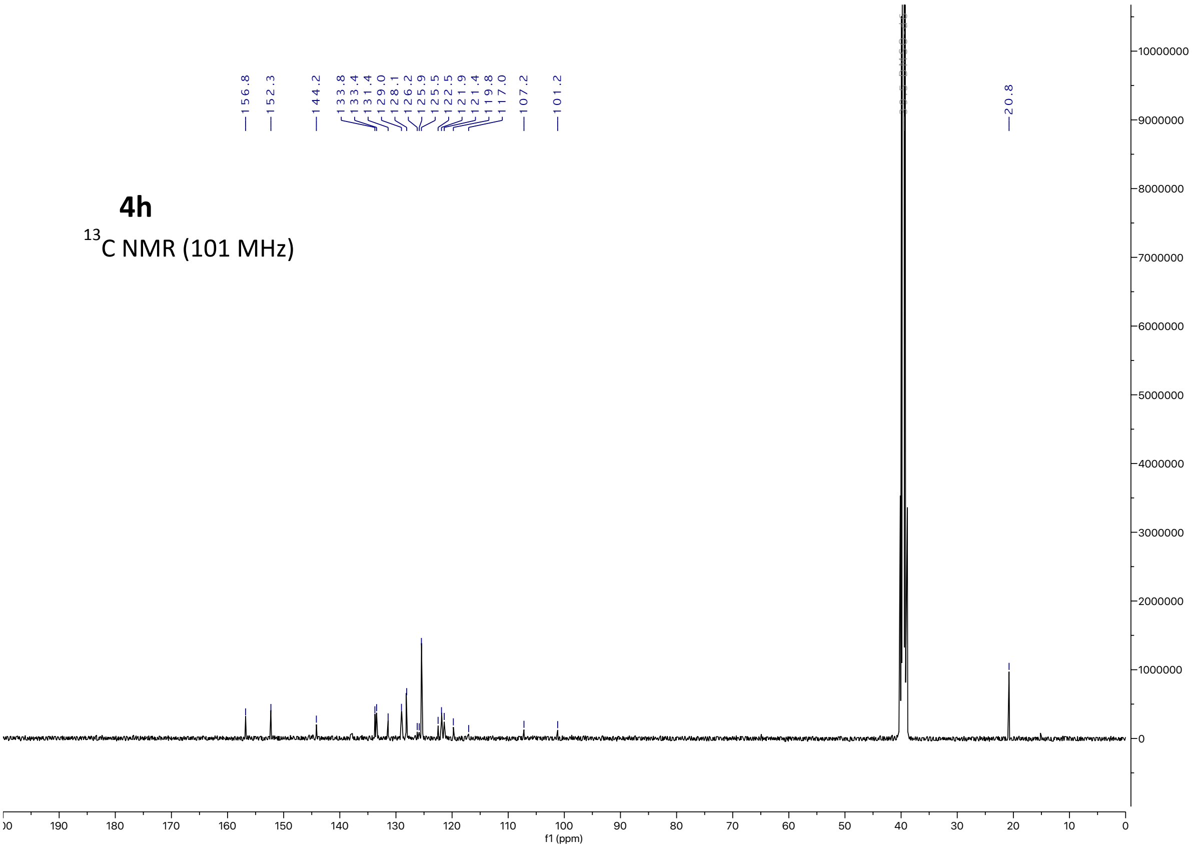

4h
^{13}C NMR (101 MHz)
f1 (ppm)

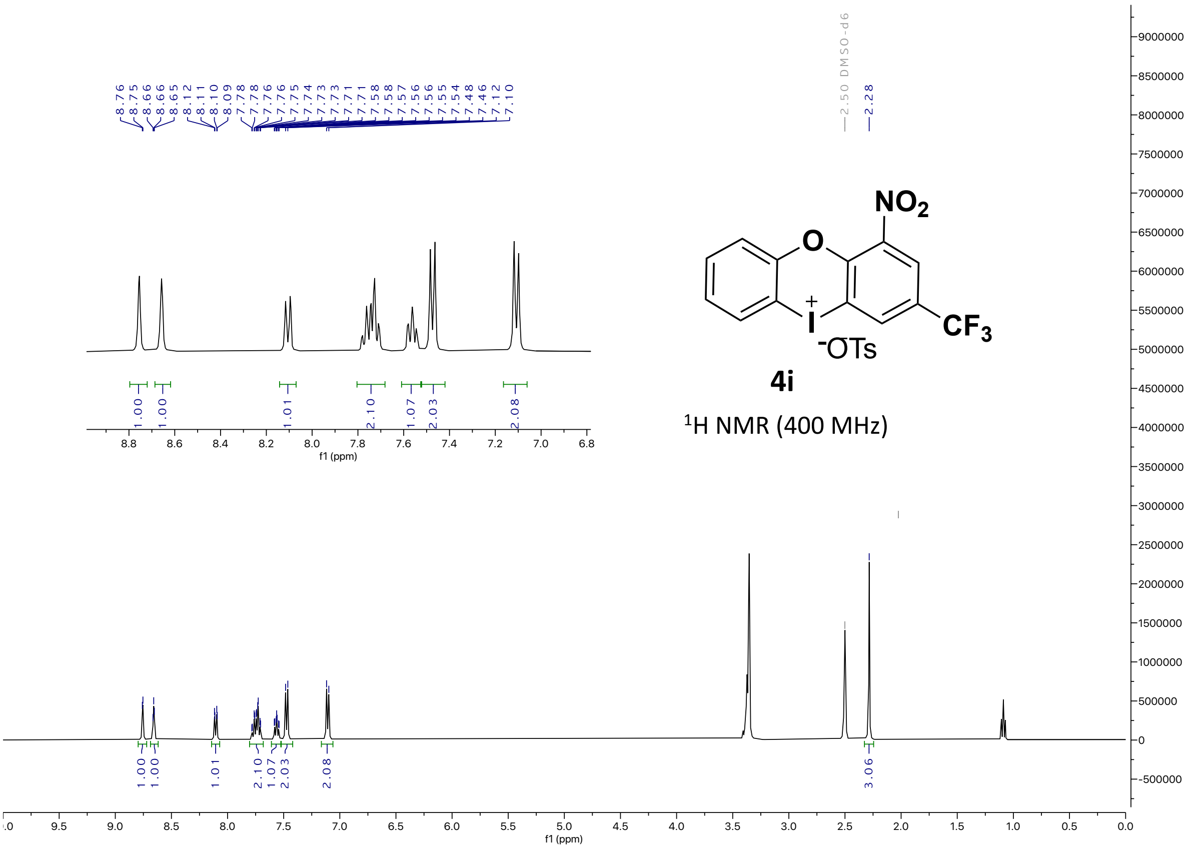
NO2
O
I
CF3
-OTs
4i
1H NMR (400 MHz)
2.50 DMSO-d6
2.28
f1 (ppm)

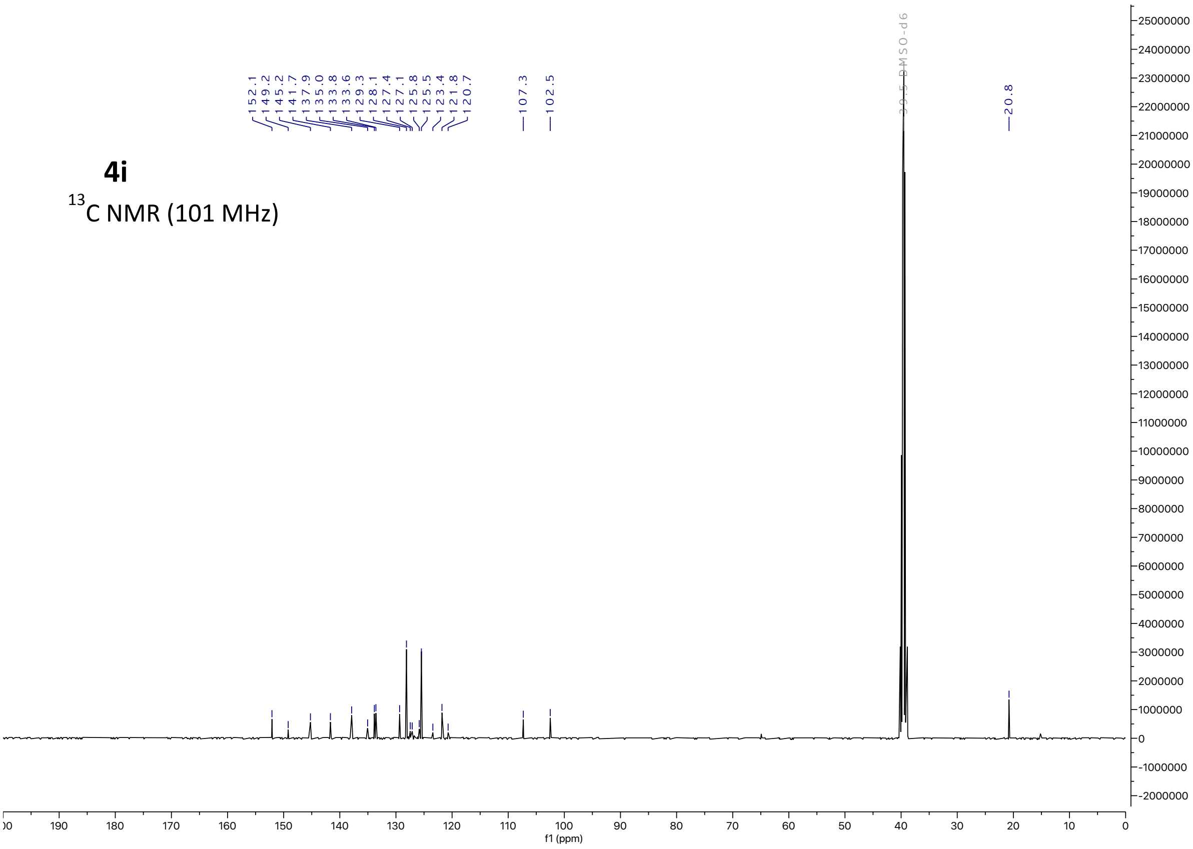
4i
13C NMR (101 MHz)
152.1
149.2
145.2
141.7
137.9
135.0
133.8
133.6
129.3
128.1
127.4
127.1
125.8
125.5
123.4
121.8
120.7
107.3
102.5
39.5 DMSO-d6
20.8
f1 (ppm)

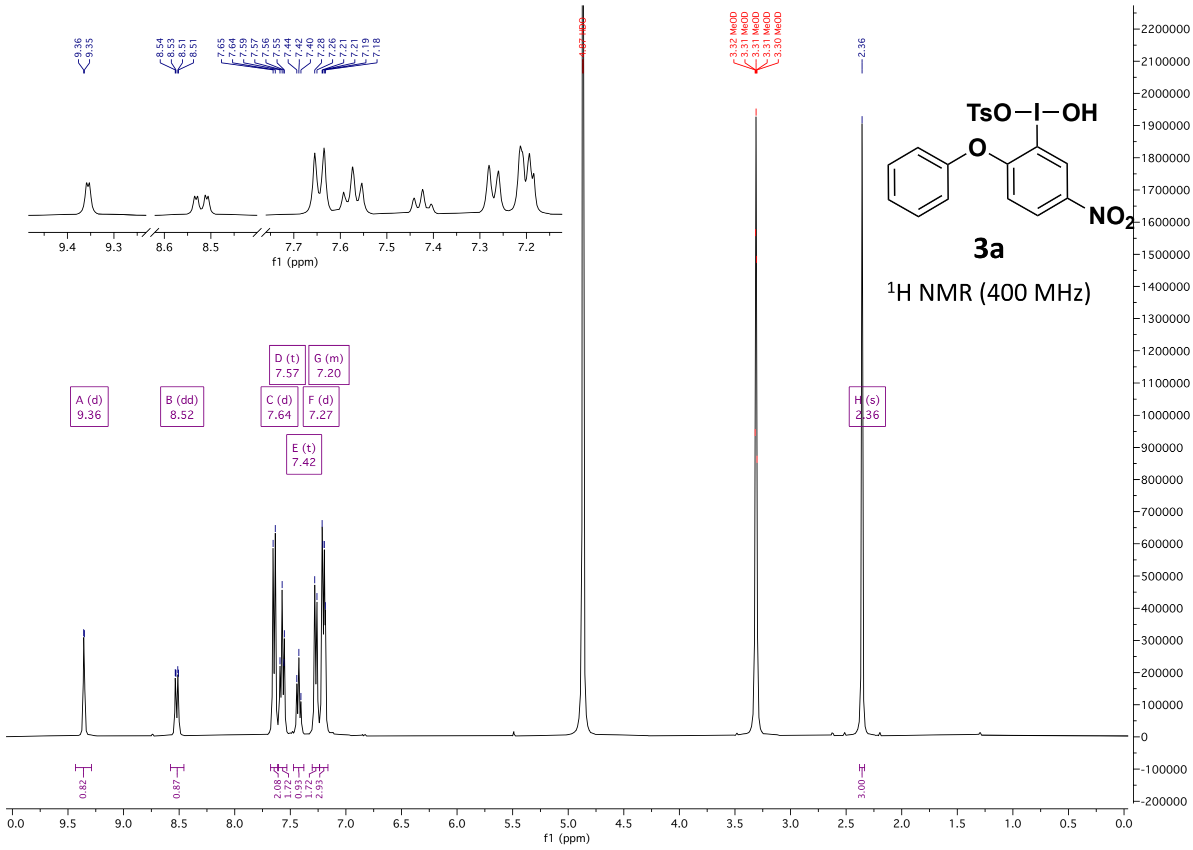
TsO—I—OH
O
NO2
3a
1H NMR (400 MHz)
A (d) 9.36
B (dd) 8.52
C (d) 7.64
D (t) 7.57
E (t) 7.42
F (d) 7.27
G (m) 7.20
H (s) 2.36
f1 (ppm)

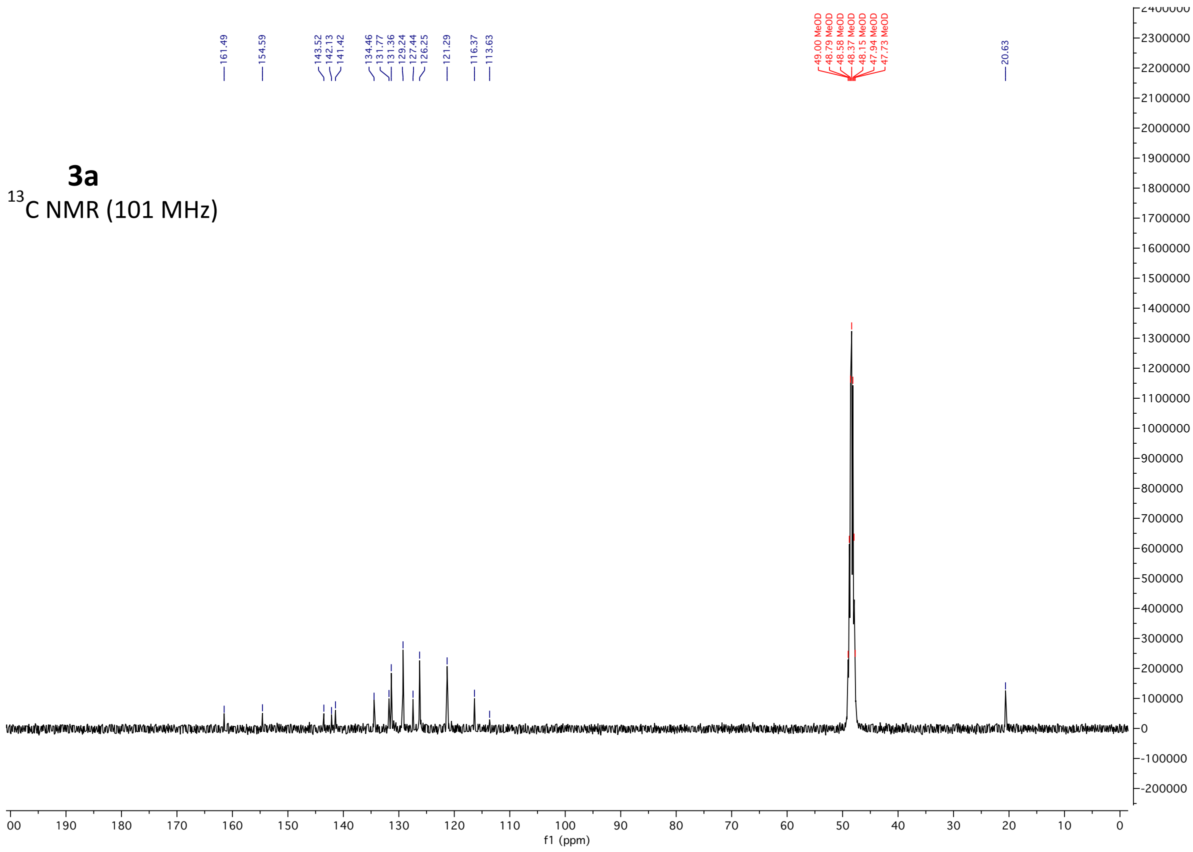
3a
13C NMR (101 MHz)
161.49
154.59
143.52
142.13
141.42
134.46
131.77
131.36
129.24
127.44
126.25
121.29
116.37
113.63
20.63
f1 (ppm)

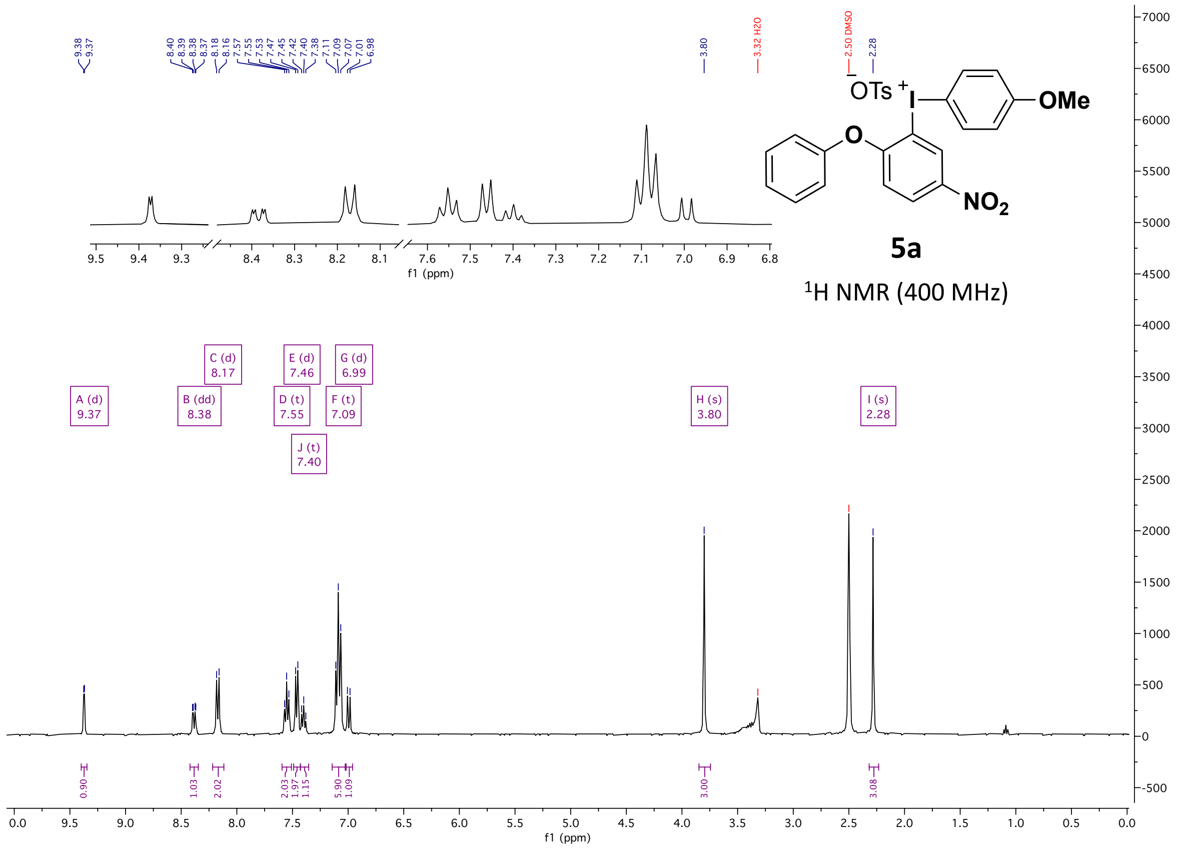
OTs
I
OMe
O
NO2
5a
1H NMR (400 MHz)
f1 (ppm)

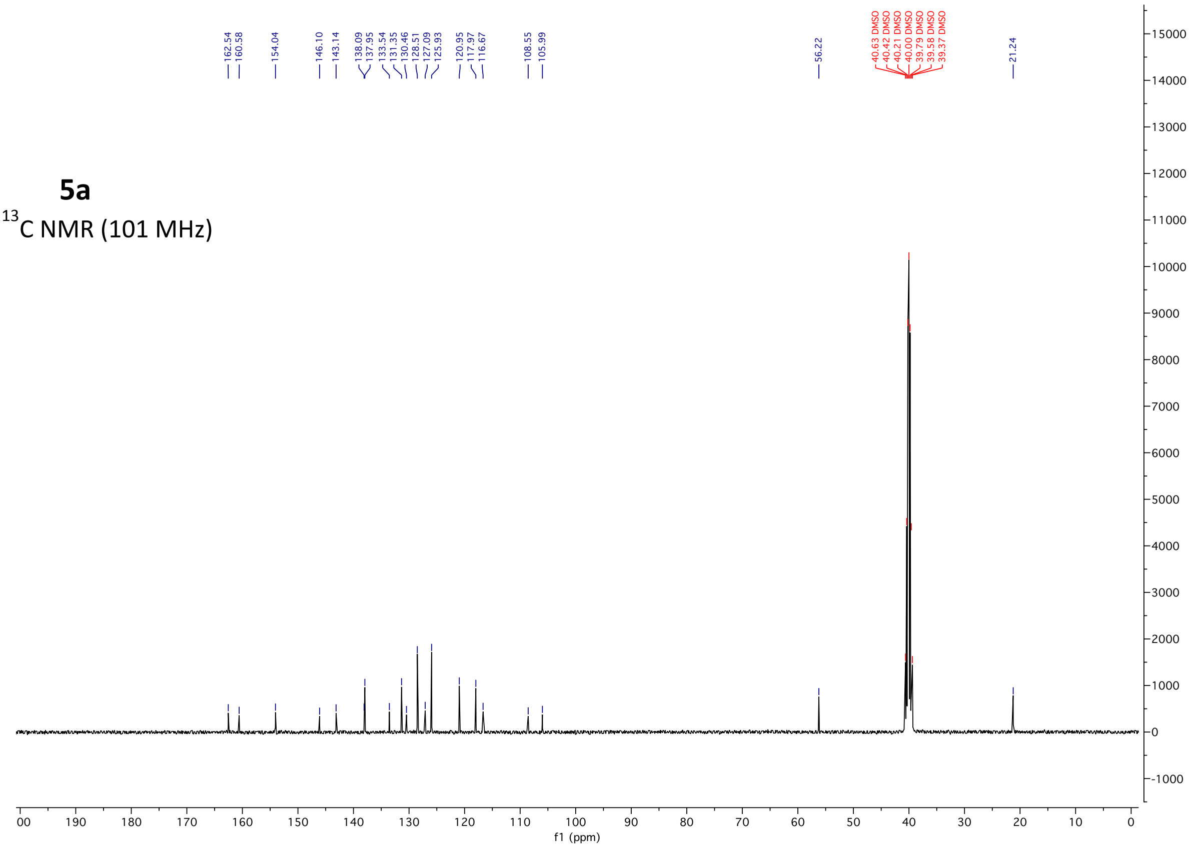
5a
13C NMR (101 MHz)
f1 (ppm)

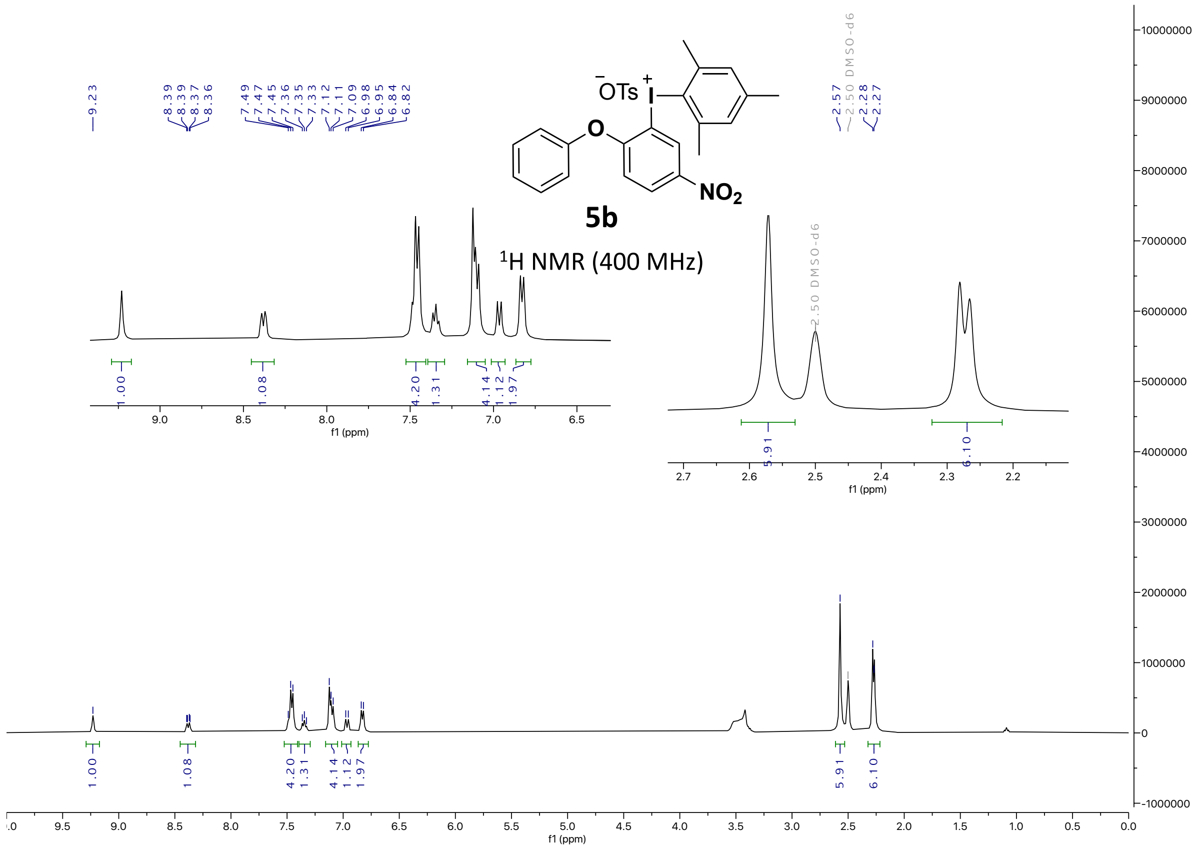

OTs
I
O
NO2
5b
1H NMR (400 MHz)
9.23
8.39
8.39
8.37
8.36
7.49
7.47
7.45
7.36
7.35
7.33
7.12
7.11
7.09
6.98
6.95
6.84
6.82
2.57
2.50 DMSO-d6
2.28
2.27
1.00
1.08
4.20
1.31
4.14
1.12
1.97
5.91
6.10
f1 (ppm)

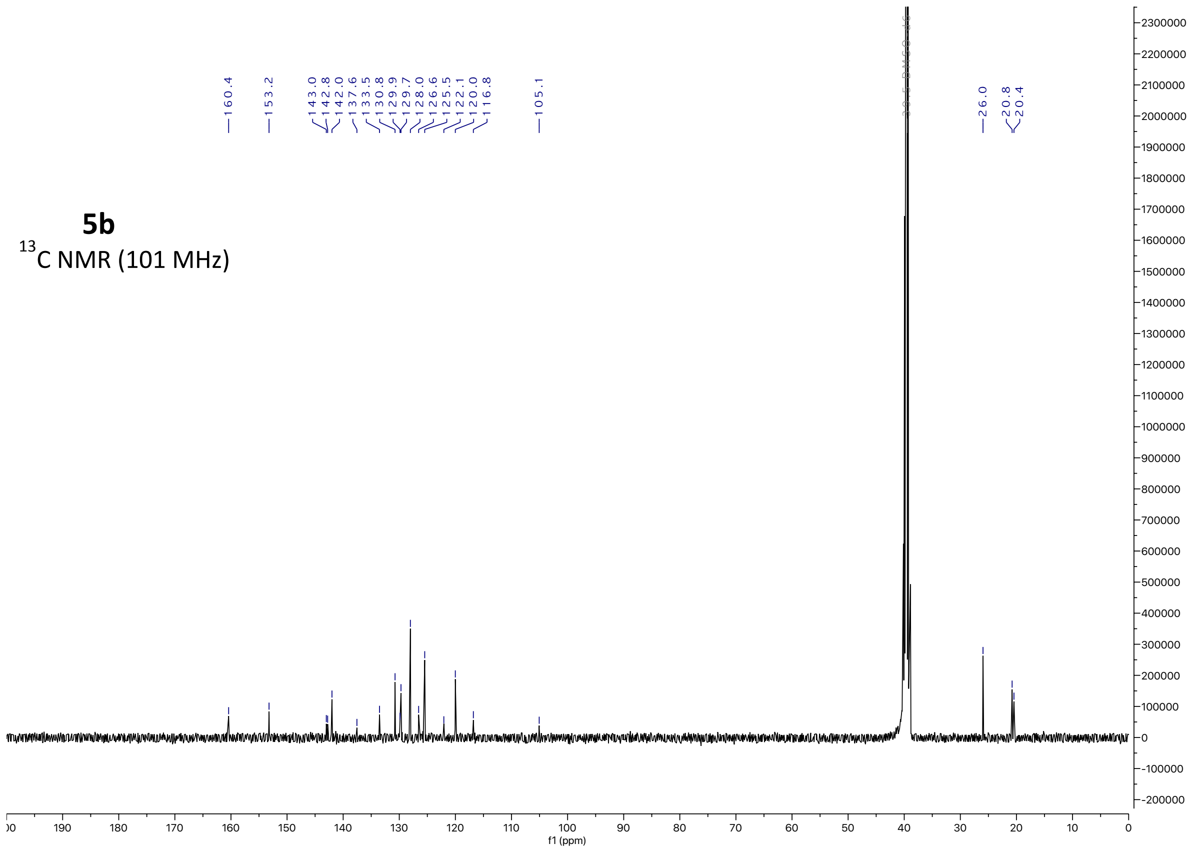

5b
13C NMR (101 MHz)
160.4
153.2
143.0
142.8
142.0
137.6
133.5
130.8
129.9
129.7
128.0
126.6
125.5
122.1
120.0
116.8
105.1
26.0
20.8
20.4
f1 (ppm)

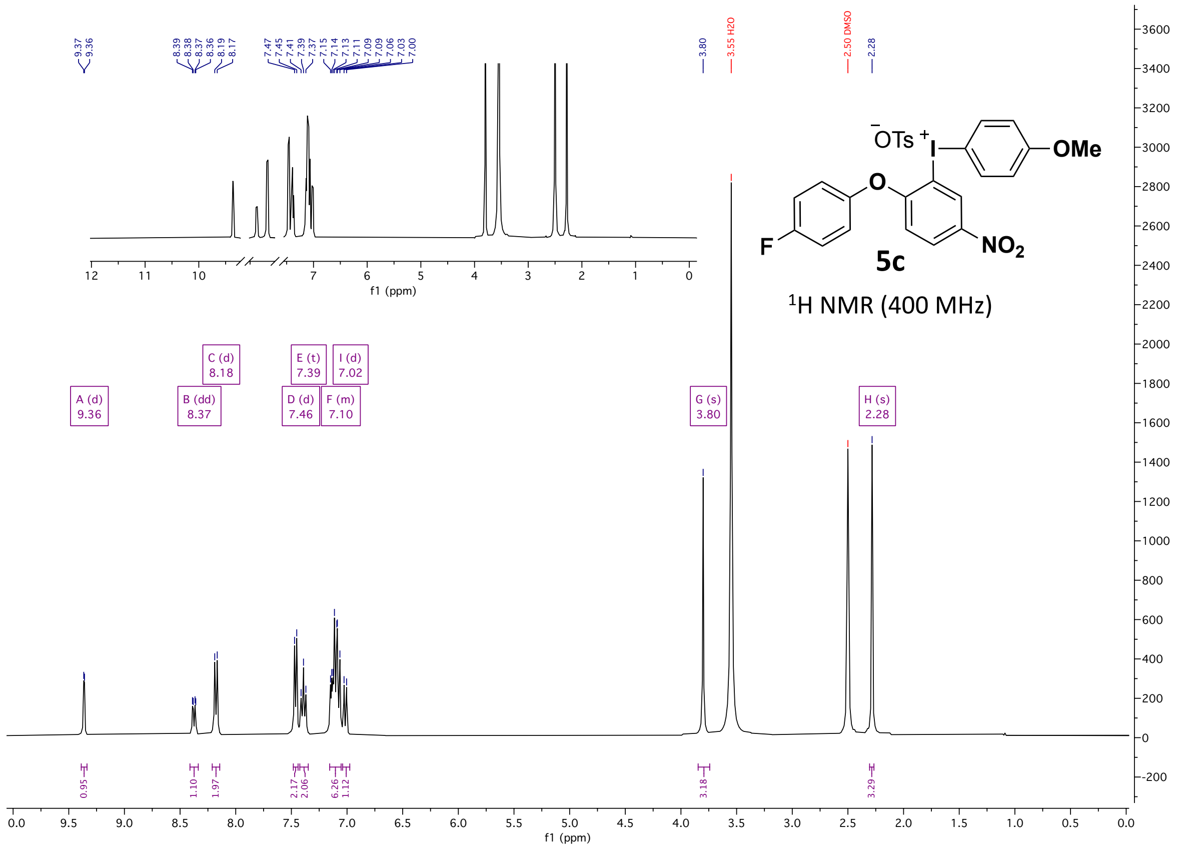
OTs
I
OMe
O
F
NO2
5c
1H NMR (400 MHz)
A (d) 9.36
B (dd) 8.37
C (d) 8.18
D (d) 7.46
E (t) 7.39
F (m) 7.10
I (d) 7.02
G (s) 3.80
H (s) 2.28
3.55 H2O
2.50 DMSO
f1 (ppm)

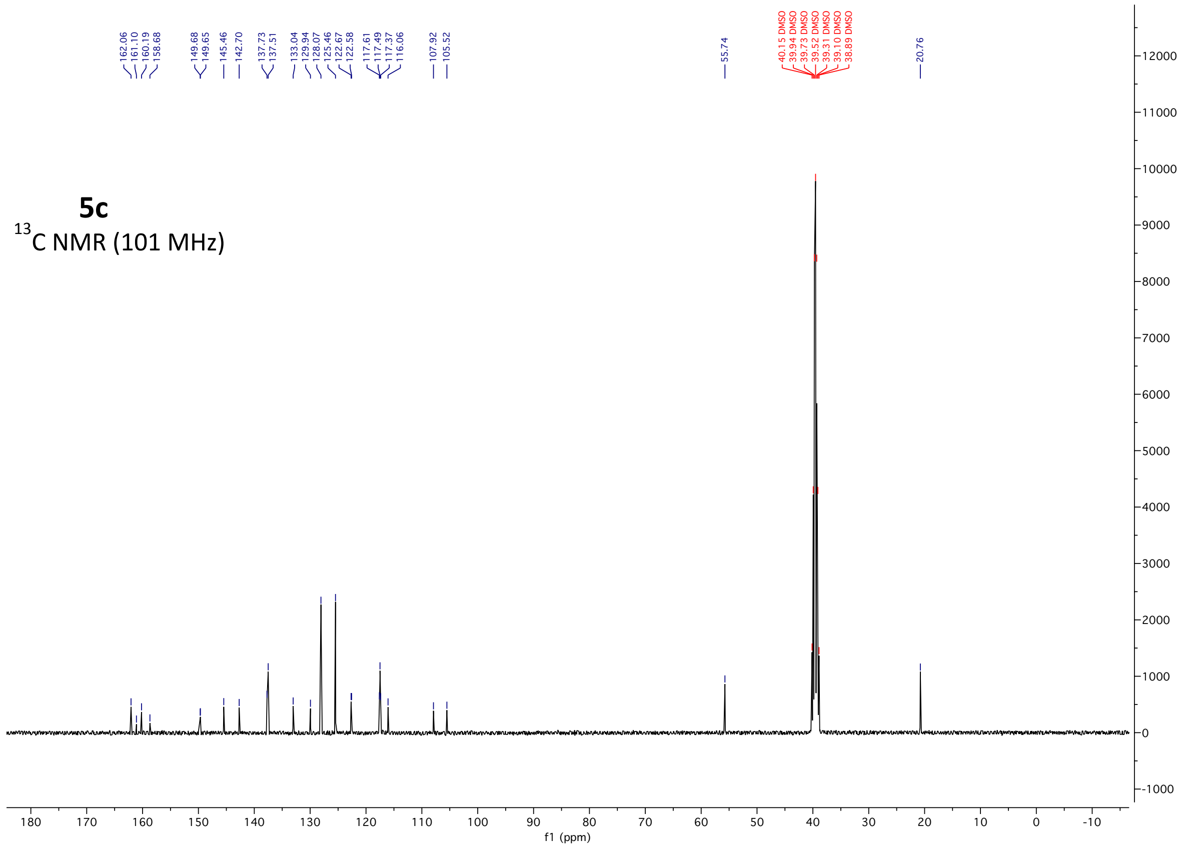
5c
13C NMR (101 MHz)
162.06
161.10
160.19
158.68
149.68
149.65
145.46
142.70
137.73
137.51
133.04
129.94
128.07
125.46
122.67
122.58
117.61
117.49
117.37
116.06
107.92
105.52
55.74
40.15 DMSO
39.94 DMSO
39.73 DMSO
39.52 DMSO
39.31 DMSO
39.10 DMSO
38.89 DMSO
20.76
f1 (ppm)

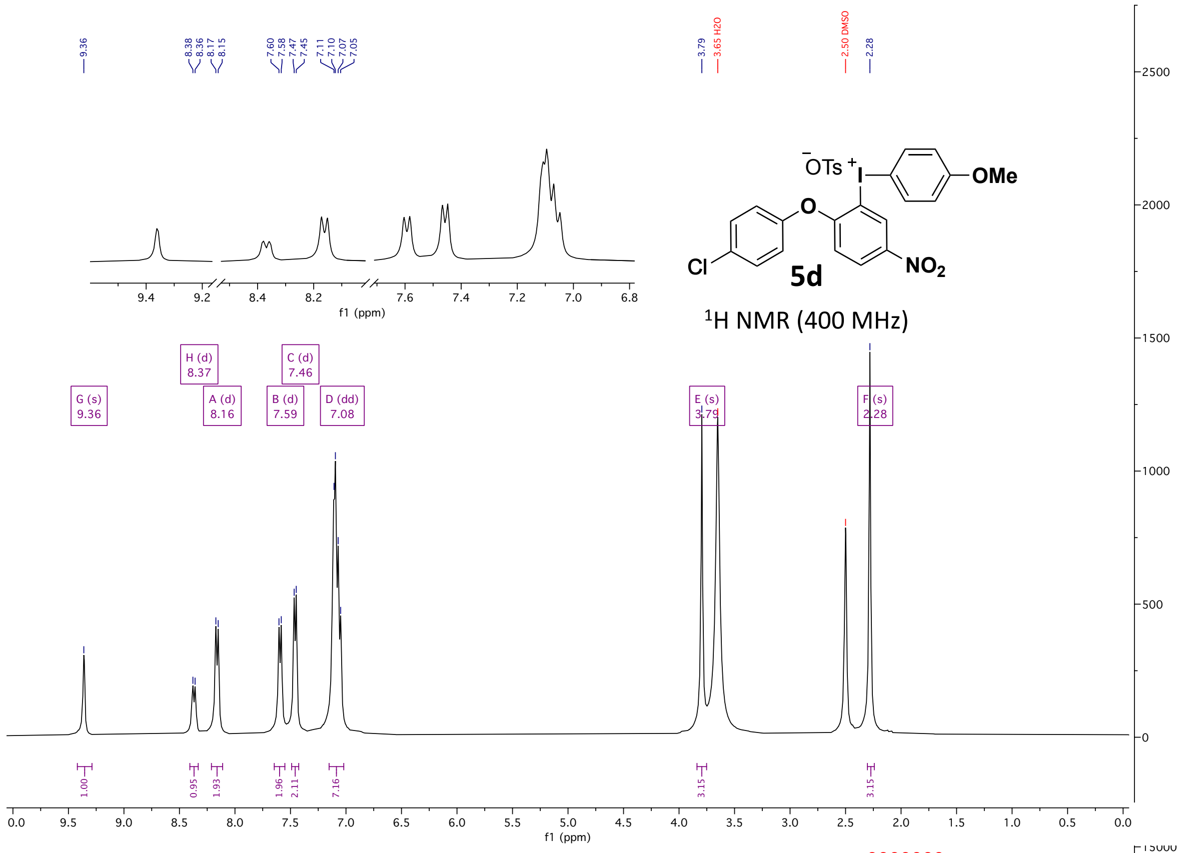

5d

^{13}C NMR (101 MHz)

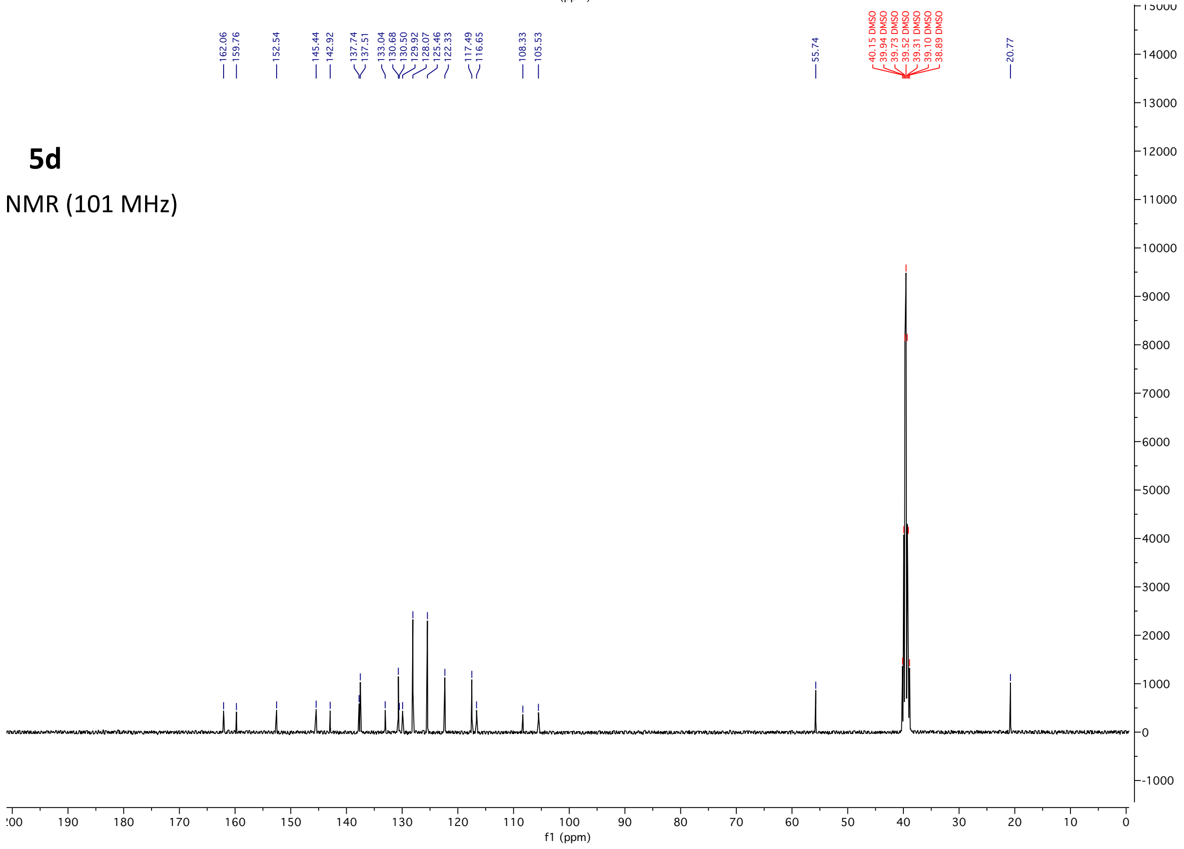

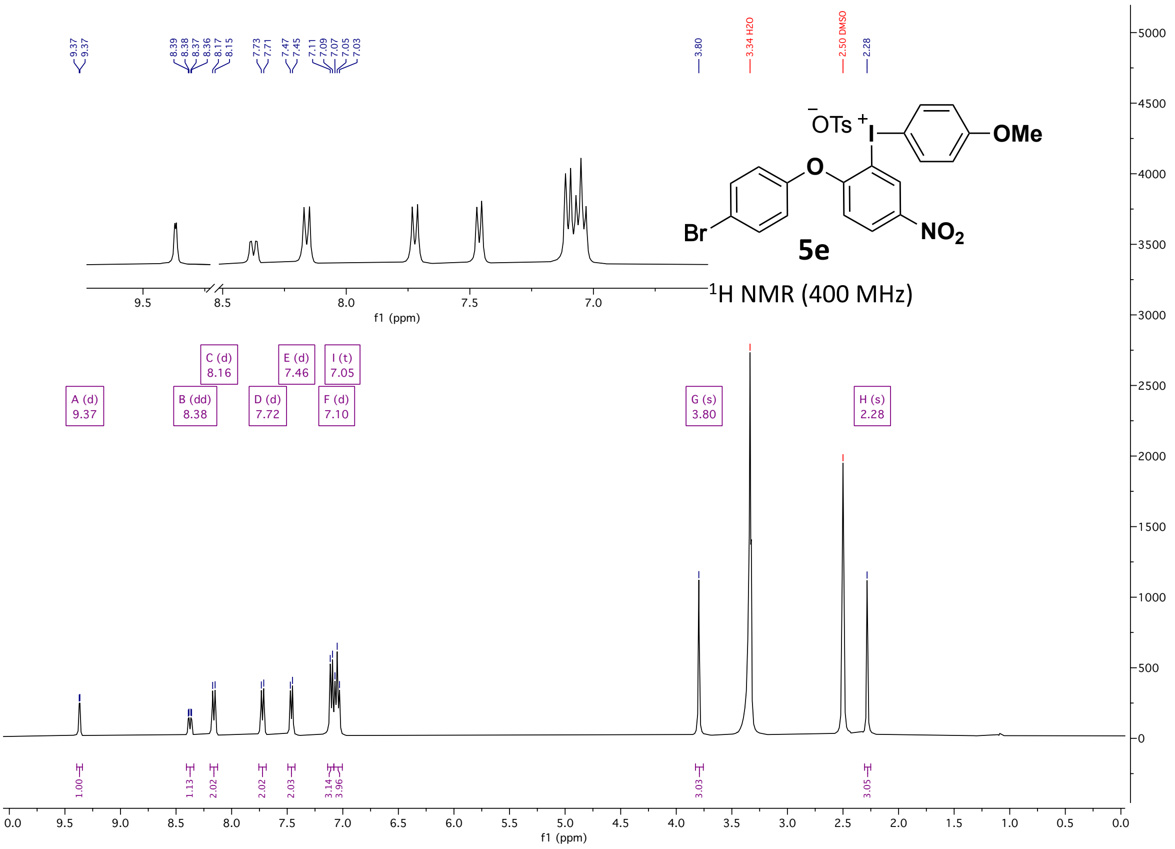
9.37
9.37
8.39
8.38
8.37
8.36
8.17
8.15
7.73
7.71
7.47
7.45
7.11
7.09
7.07
7.05
7.03
3.80
3.34 H2O
2.50 DMSO
2.28
OTs
I
OMe
O
Br
NO2
5e
1H NMR (400 MHz)
A (d) 9.37
B (dd) 8.38
C (d) 8.16
D (d) 7.72
E (d) 7.46
F (d) 7.10
I (t) 7.05
G (s) 3.80
H (s) 2.28
f1 (ppm)

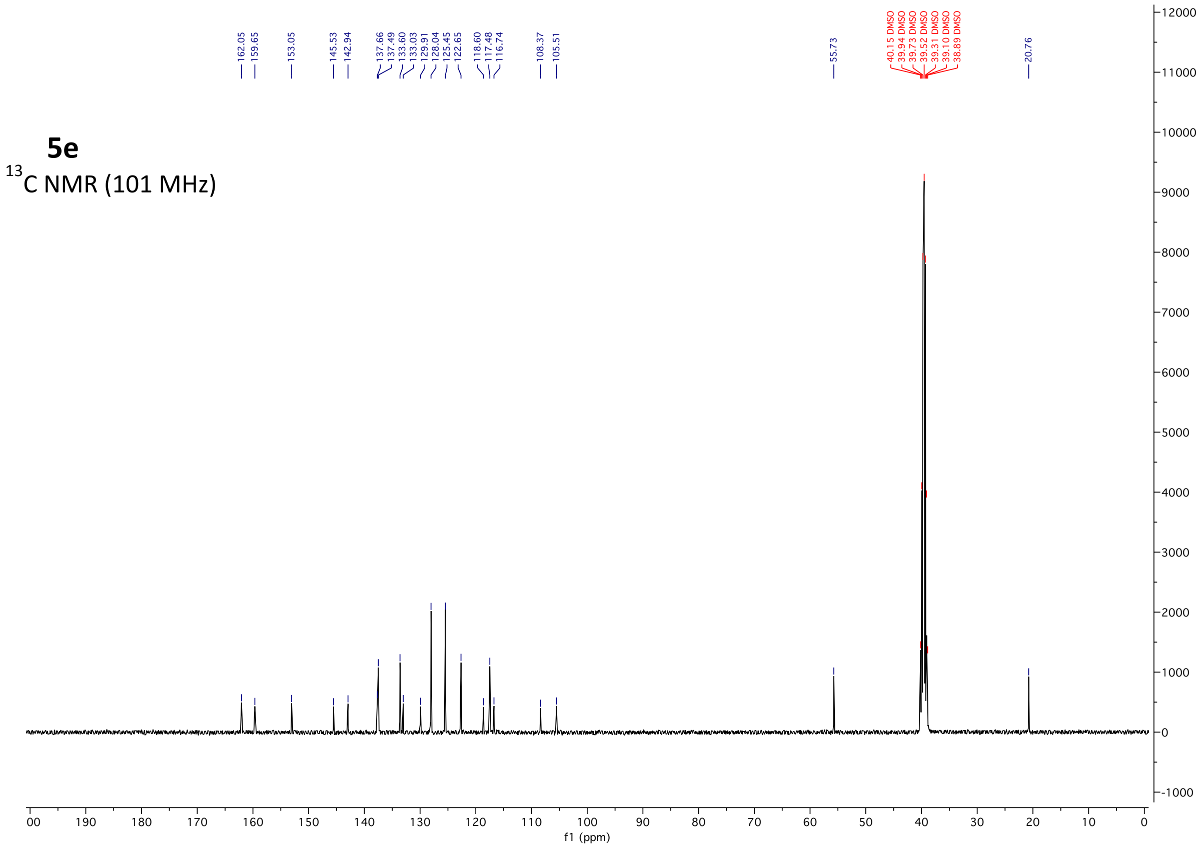
162.05
159.65
153.05
145.53
142.94
137.66
137.49
133.60
133.03
129.91
128.04
125.45
122.65
118.60
117.48
116.74
108.37
105.51
55.73
40.15 DMSO
39.94 DMSO
39.73 DMSO
39.52 DMSO
39.31 DMSO
39.10 DMSO
38.89 DMSO
20.76
5e
13C NMR (101 MHz)
f1 (ppm)

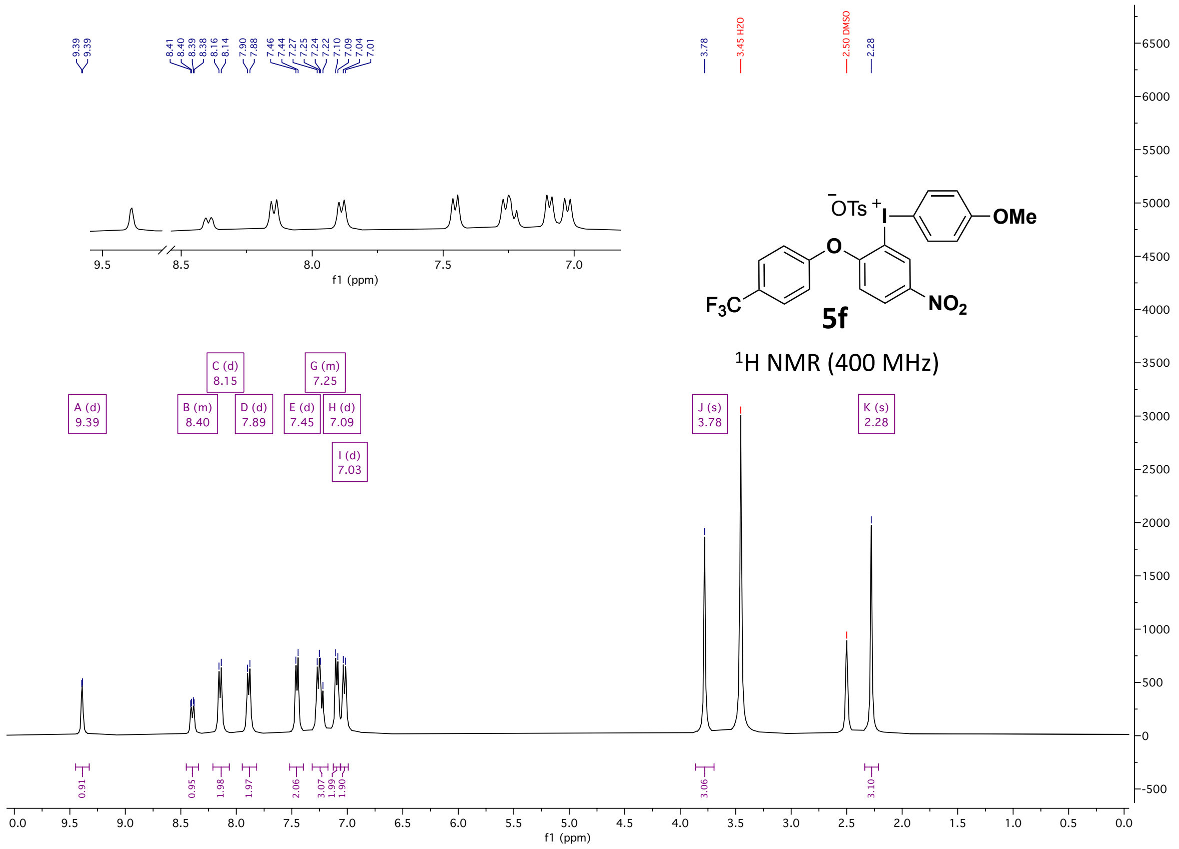
5f
^{1}H NMR (400 MHz)
f1 (ppm)

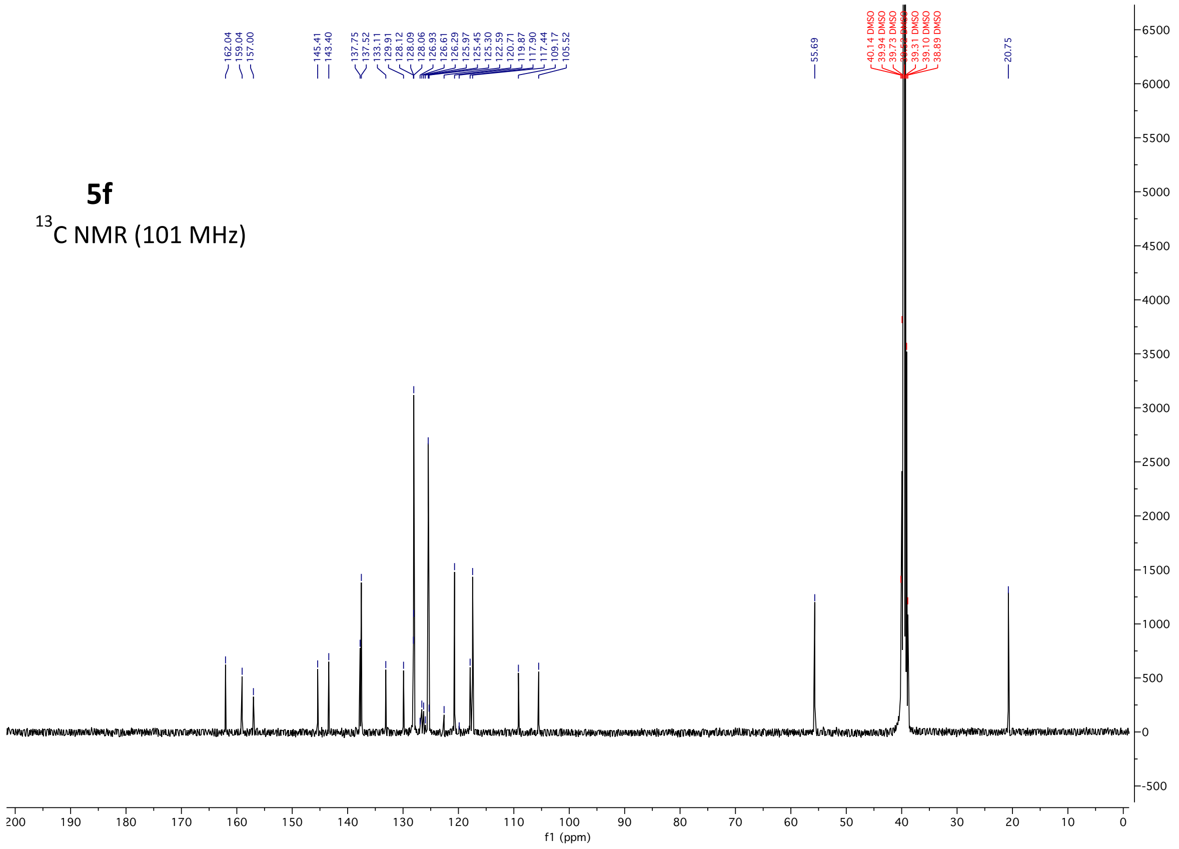
5f
^{13}C NMR (101 MHz)
f1 (ppm)

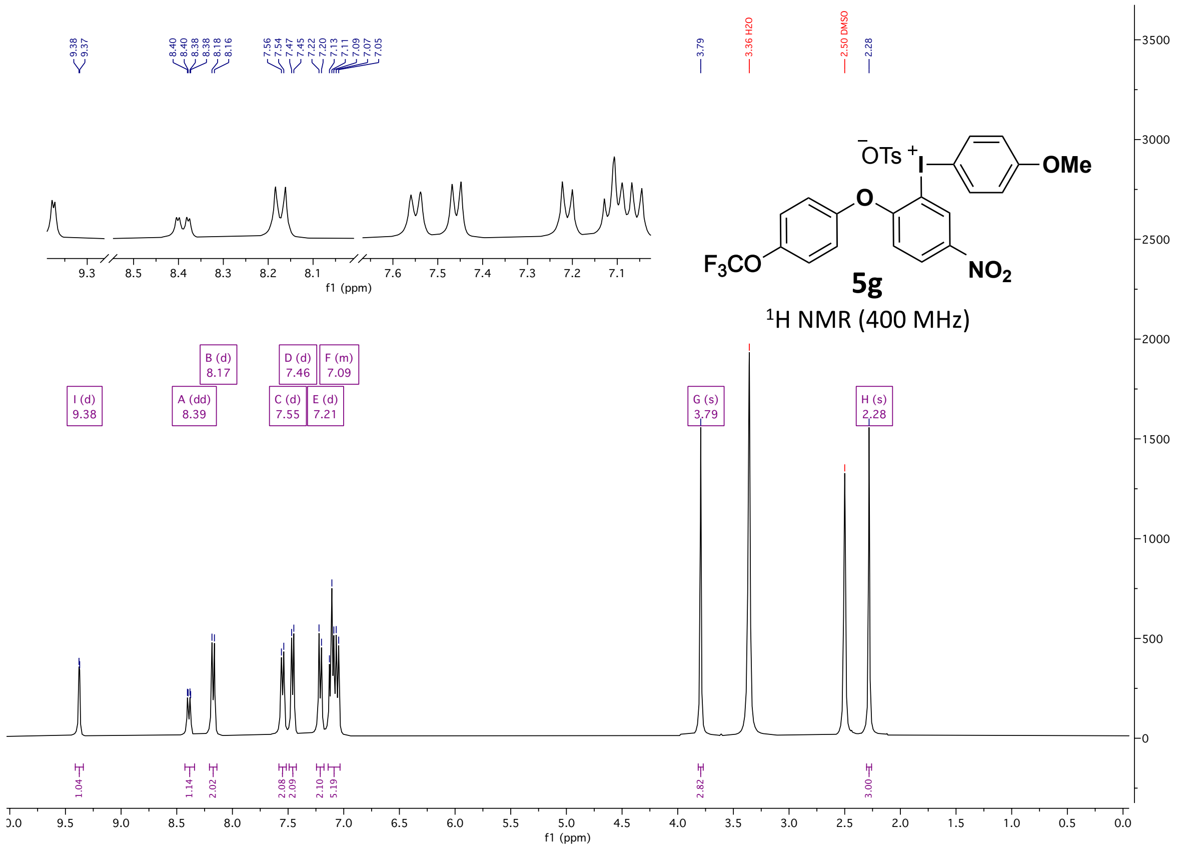

5g
^{1}H NMR (400 MHz)
I (d) 9.38
A (dd) 8.39
B (d) 8.17
C (d) 7.55
D (d) 7.46
E (d) 7.21
F (m) 7.09
G (s) 3.79
H (s) 2.28
3.36 H2O
2.50 DMSO
f1 (ppm)

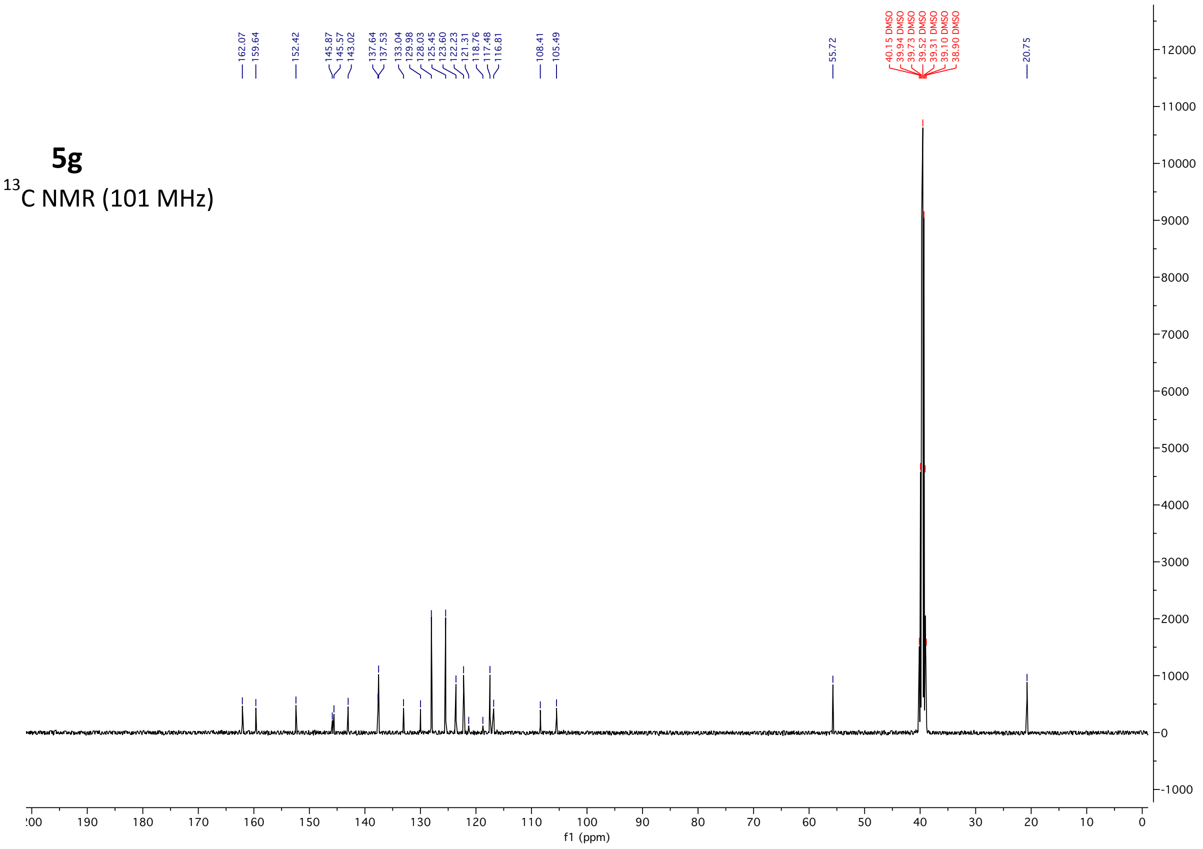

5g
^{13}C NMR (101 MHz)
162.07
159.64
152.42
145.87
145.57
143.02
137.64
137.53
133.04
129.98
128.03
125.45
123.60
122.23
121.31
118.76
117.48
116.81
108.41
105.49
55.72
40.15 DMSO
39.94 DMSO
39.73 DMSO
39.52 DMSO
39.31 DMSO
39.10 DMSO
38.90 DMSO
20.75
f1 (ppm)

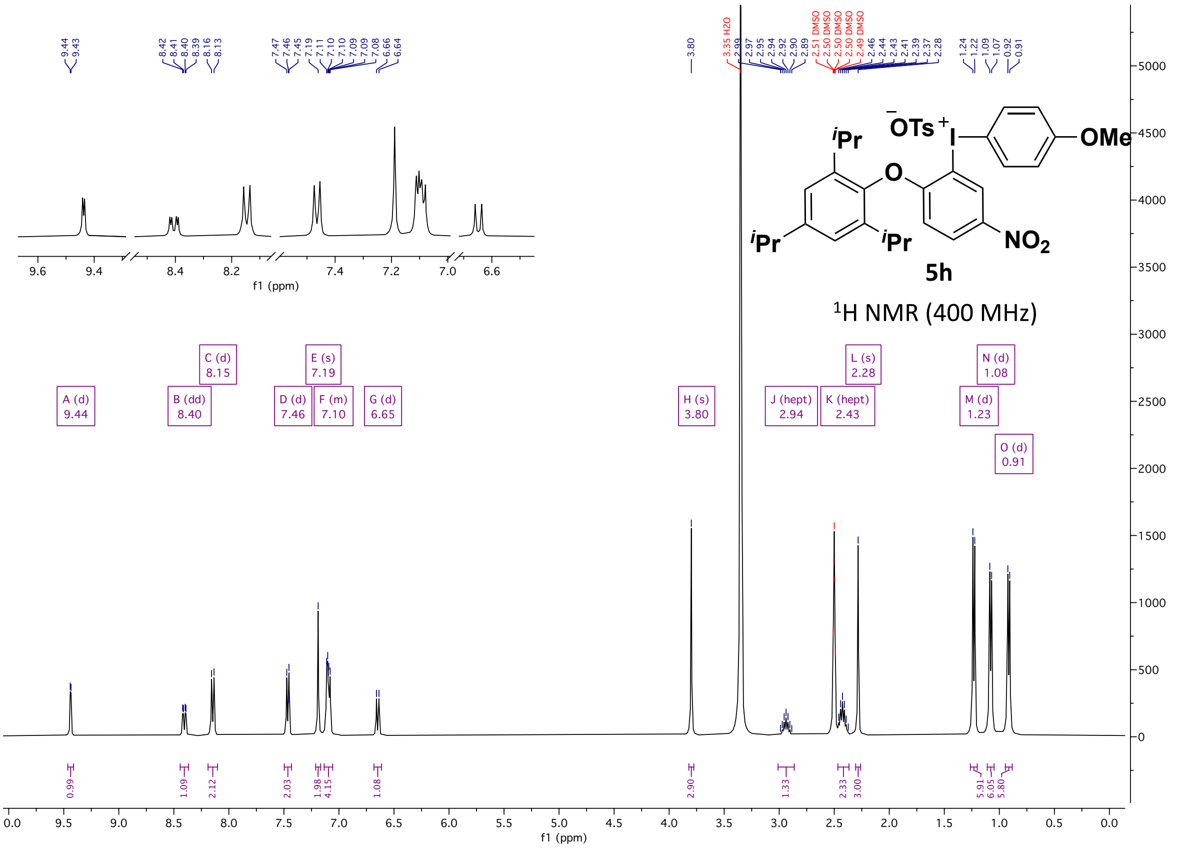
OTs
I
OMe
iPr
O
iPr
iPr
NO2
5h
1H NMR (400 MHz)
A (d) 9.44
B (dd) 8.40
C (d) 8.15
D (d) 7.46
E (s) 7.19
F (m) 7.10
G (d) 6.65
H (s) 3.80
J (hept) 2.94
K (hept) 2.43
L (s) 2.28
M (d) 1.23
N (d) 1.08
O (d) 0.91
3.35 H2O
2.51 DMSO
2.50 DMSO
2.50 DMSO
2.50 DMSO
2.49 DMSO
f1 (ppm)

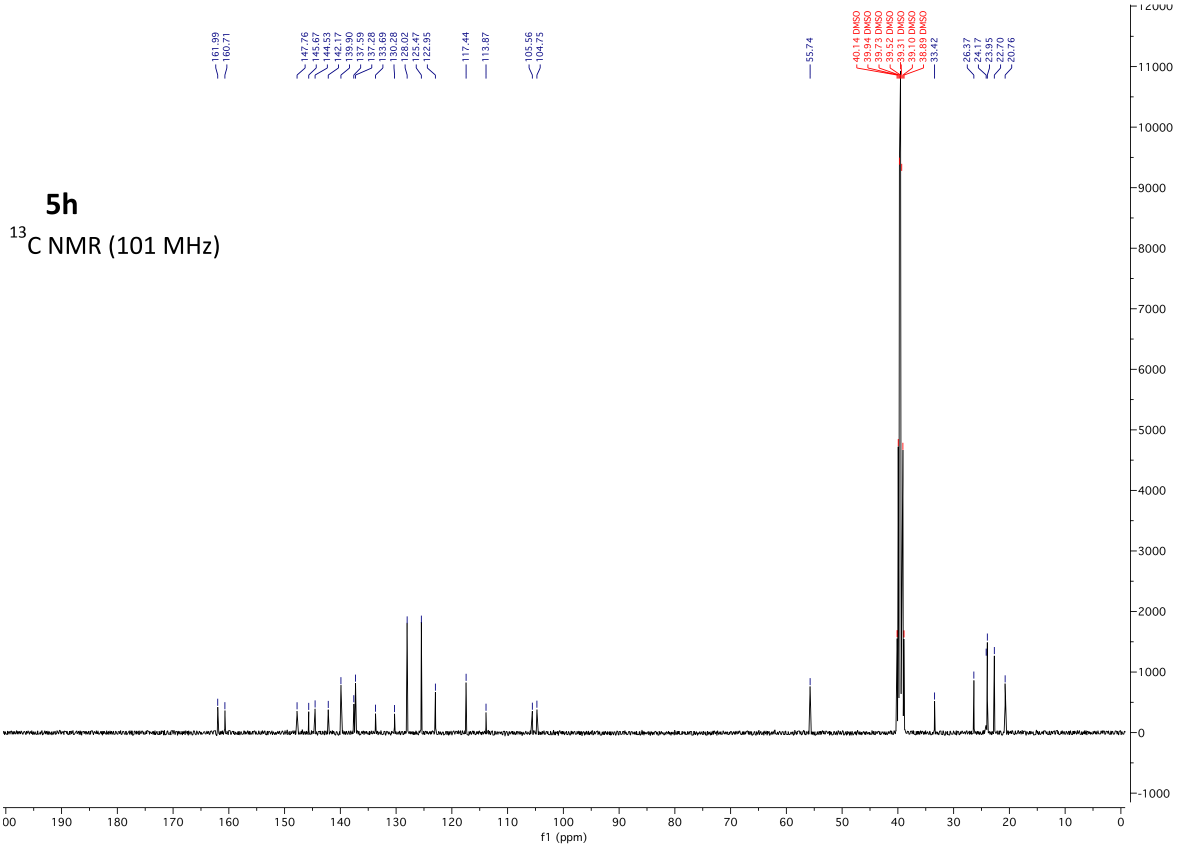
5h
13C NMR (101 MHz)
161.99
160.71
147.76
145.67
144.53
142.17
139.90
137.59
137.28
133.69
130.28
128.02
125.47
122.95
117.44
113.87
105.56
104.75
55.74
40.14 DMSO
39.94 DMSO
39.73 DMSO
39.52 DMSO
39.31 DMSO
39.10 DMSO
38.89 DMSO
33.42
26.37
24.17
23.95
22.70
20.76
f1 (ppm)

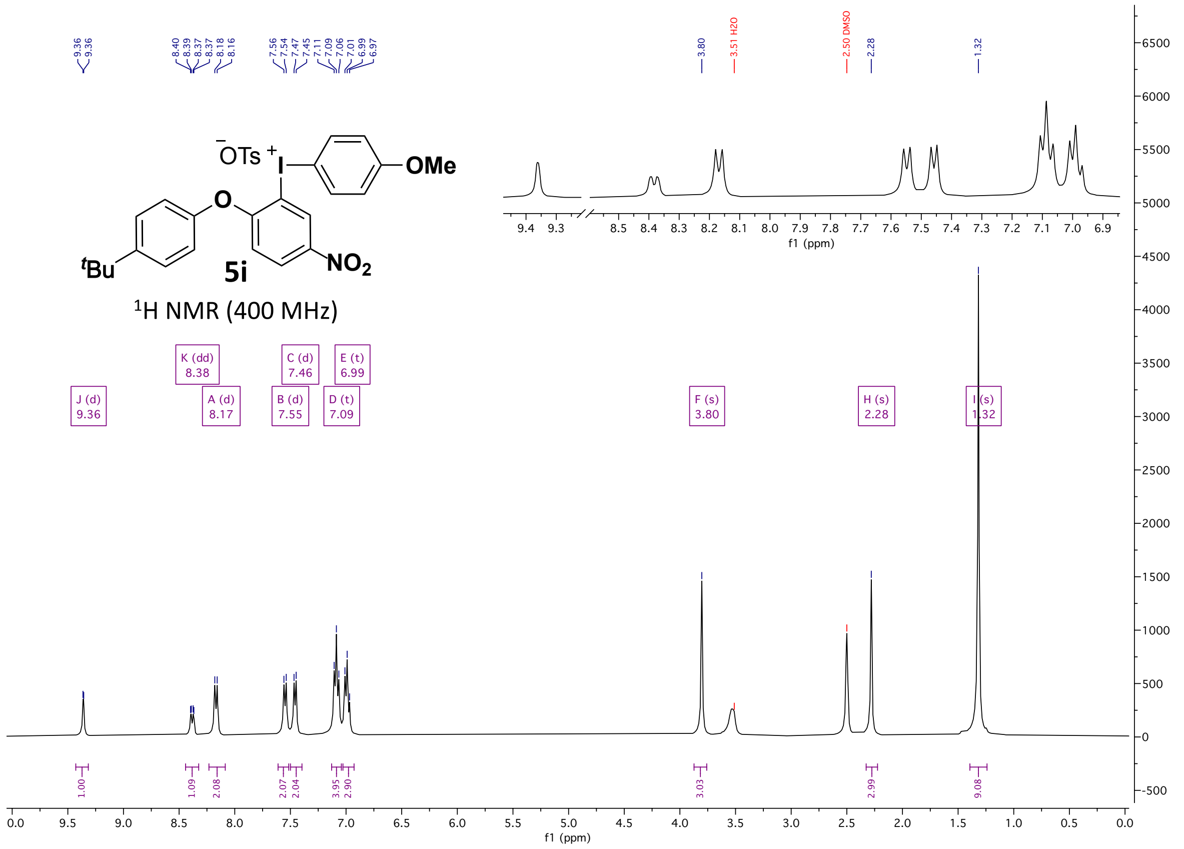
5i
1H NMR (400 MHz)
f1 (ppm)

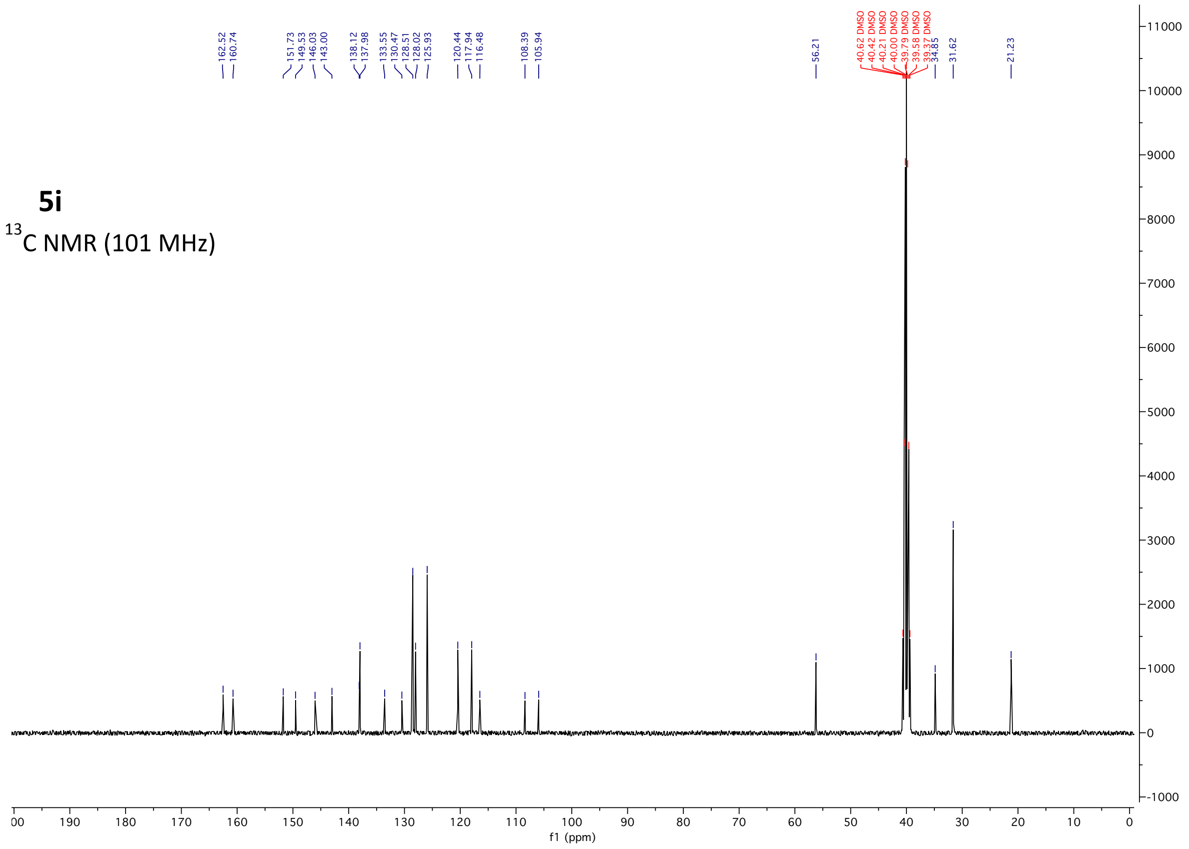
5i
13C NMR (101 MHz)
f1 (ppm)

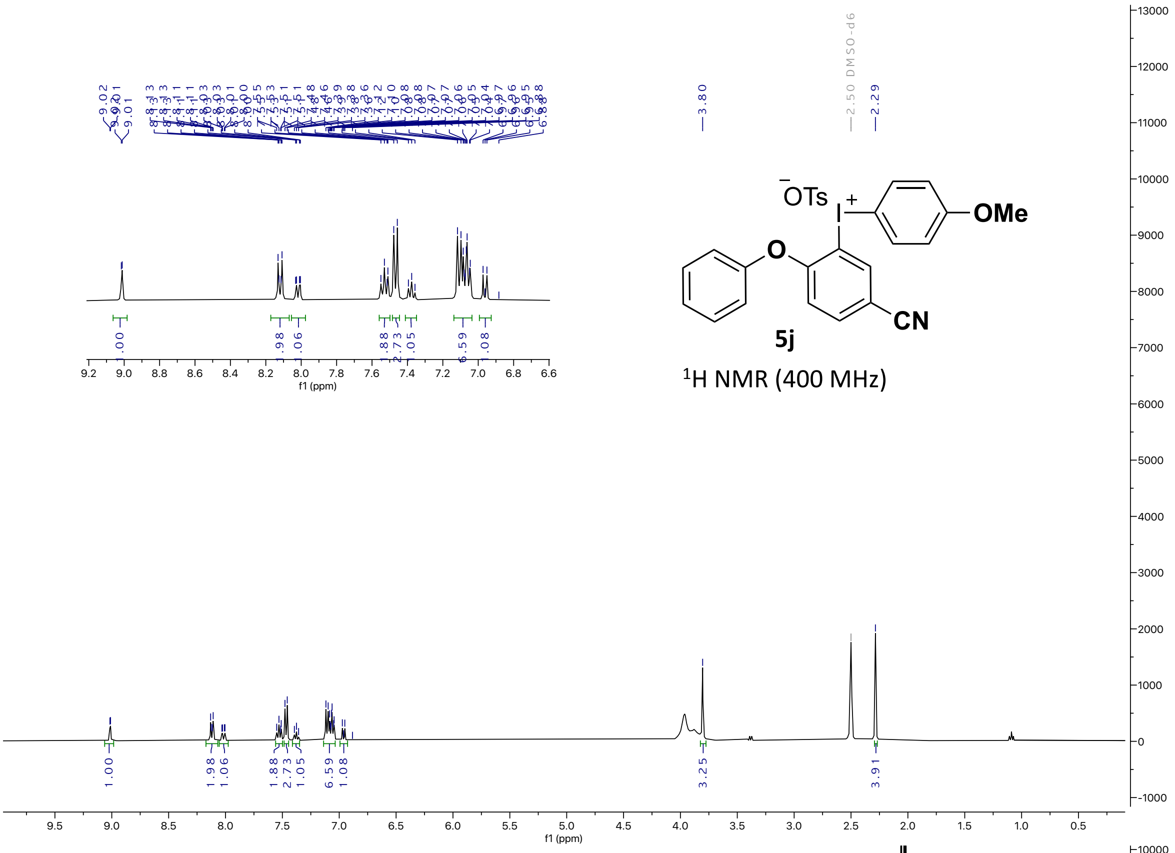
OTs
OMe
O
I
CN
5j
¹H NMR (400 MHz)
3.80
2.50 DMSO-d6
2.29
f1 (ppm)

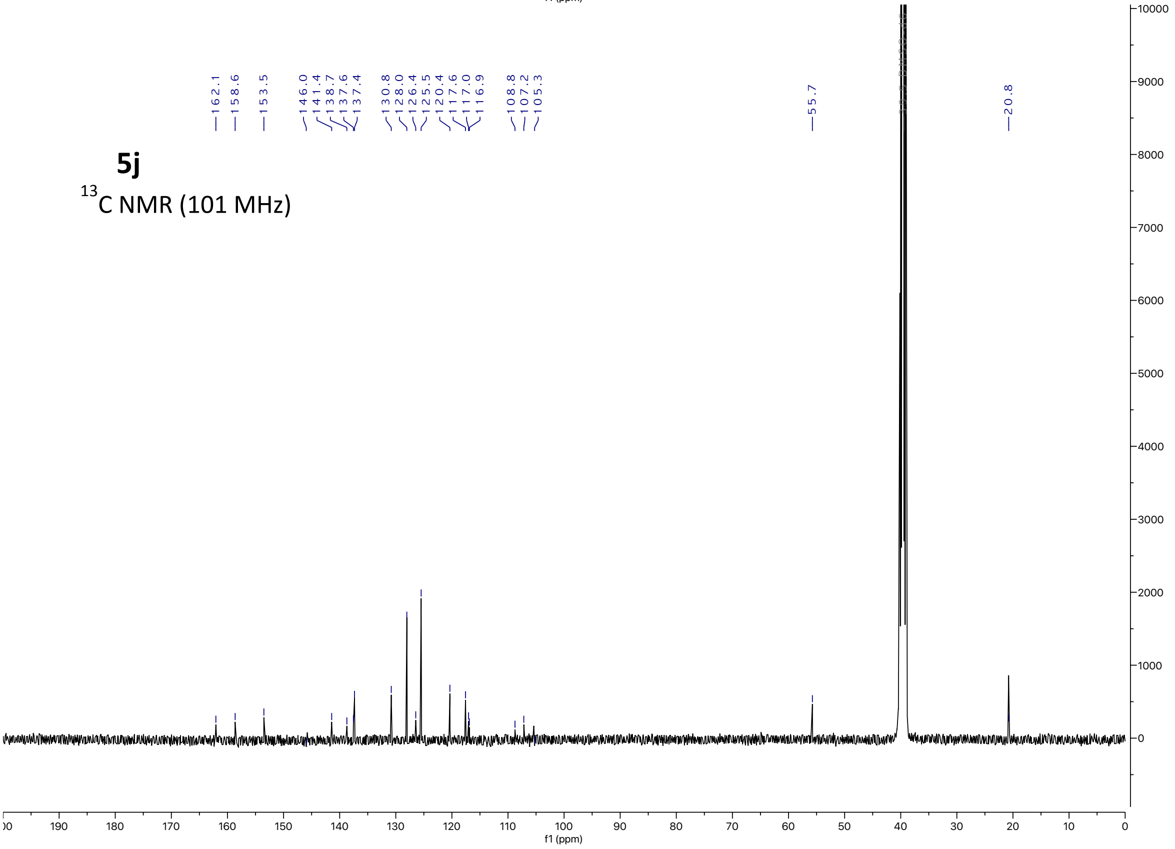
5j
¹³C NMR (101 MHz)
162.1
158.6
153.5
146.0
141.4
138.7
137.6
137.4
130.8
128.0
126.4
125.5
120.4
117.6
117.0
116.9
108.8
107.2
105.3
55.7
20.8
f1 (ppm)

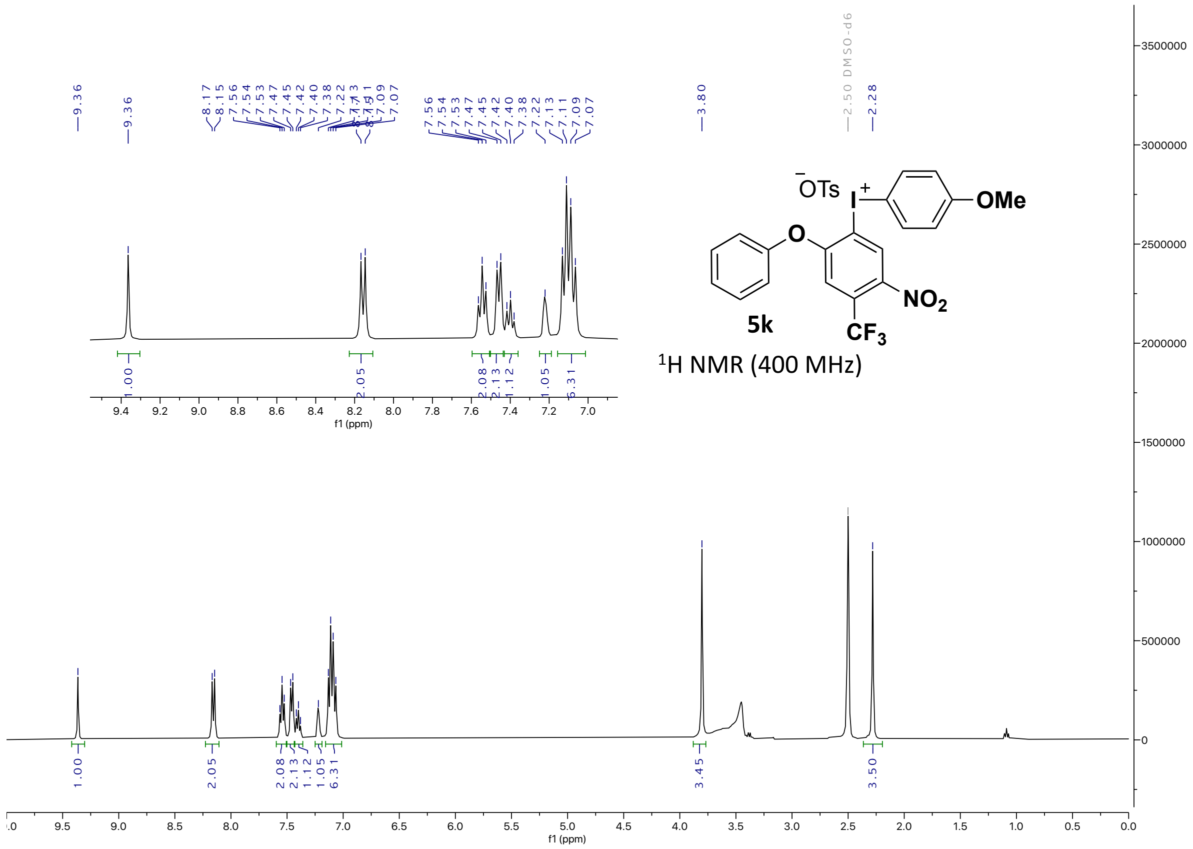

OTs
I
OMe
O
NO2
CF3
5k
1H NMR (400 MHz)

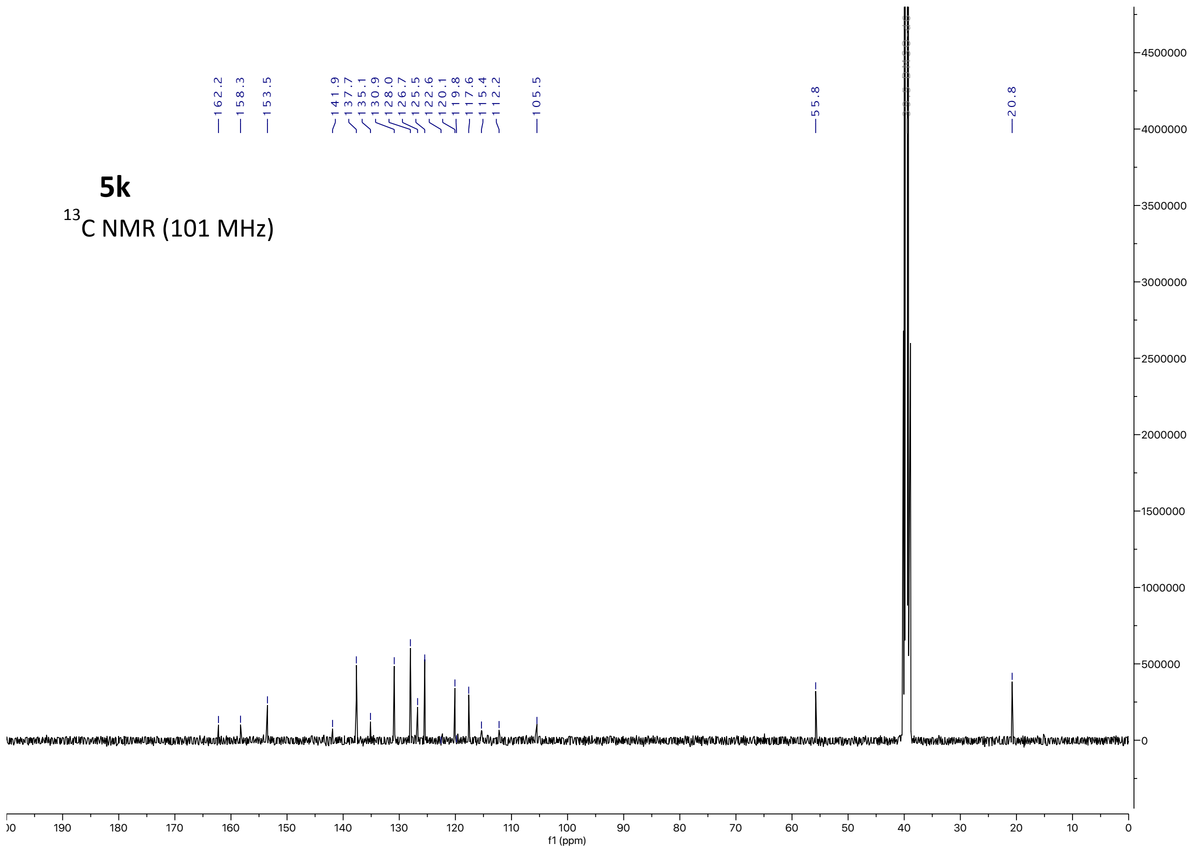

5k
13C NMR (101 MHz)

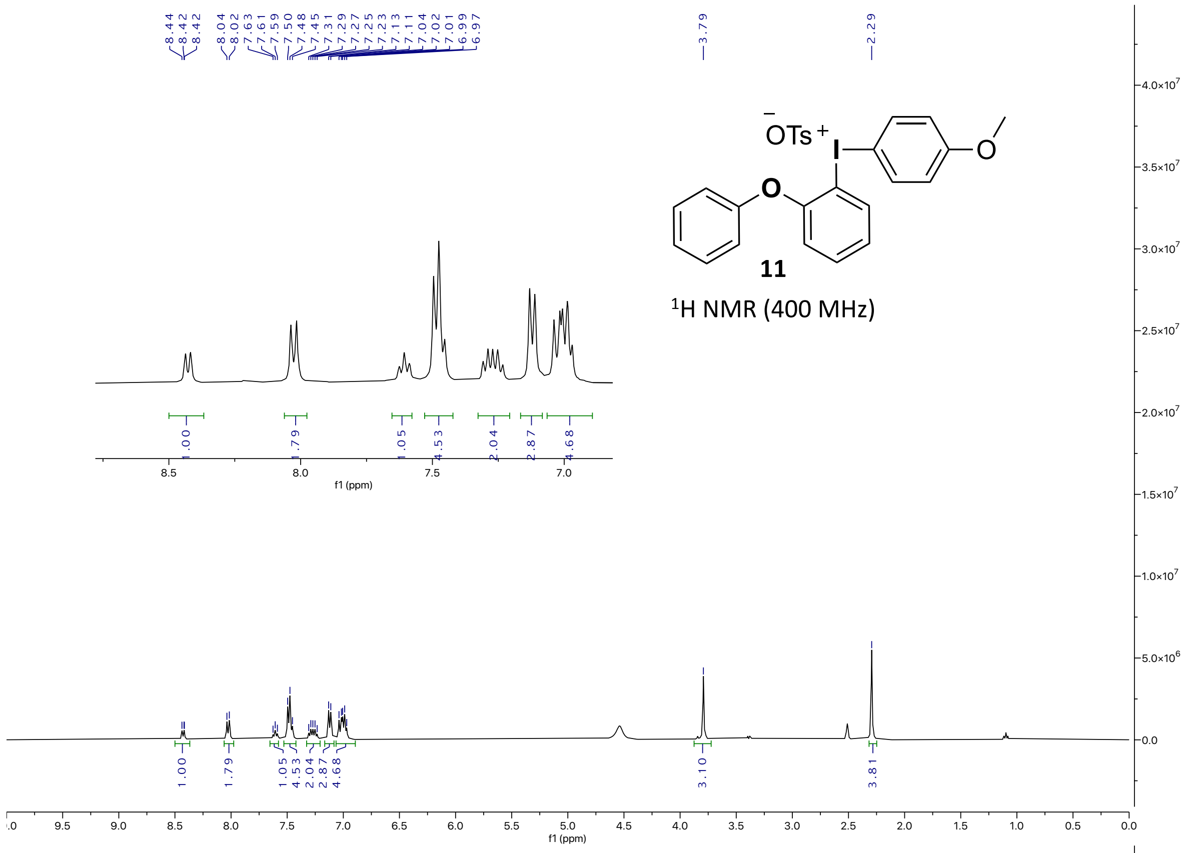

11

^{1}H NMR (400 MHz)

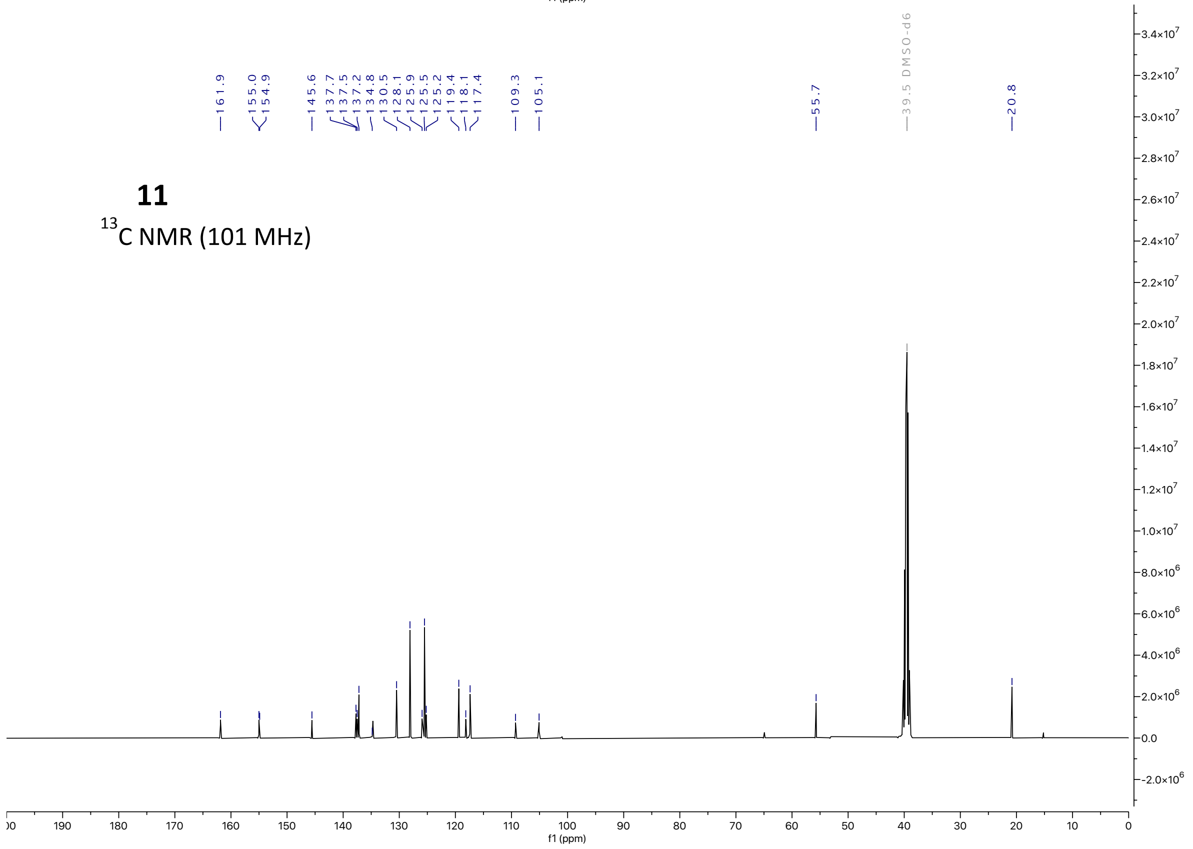

11

^{13}C NMR (101 MHz)

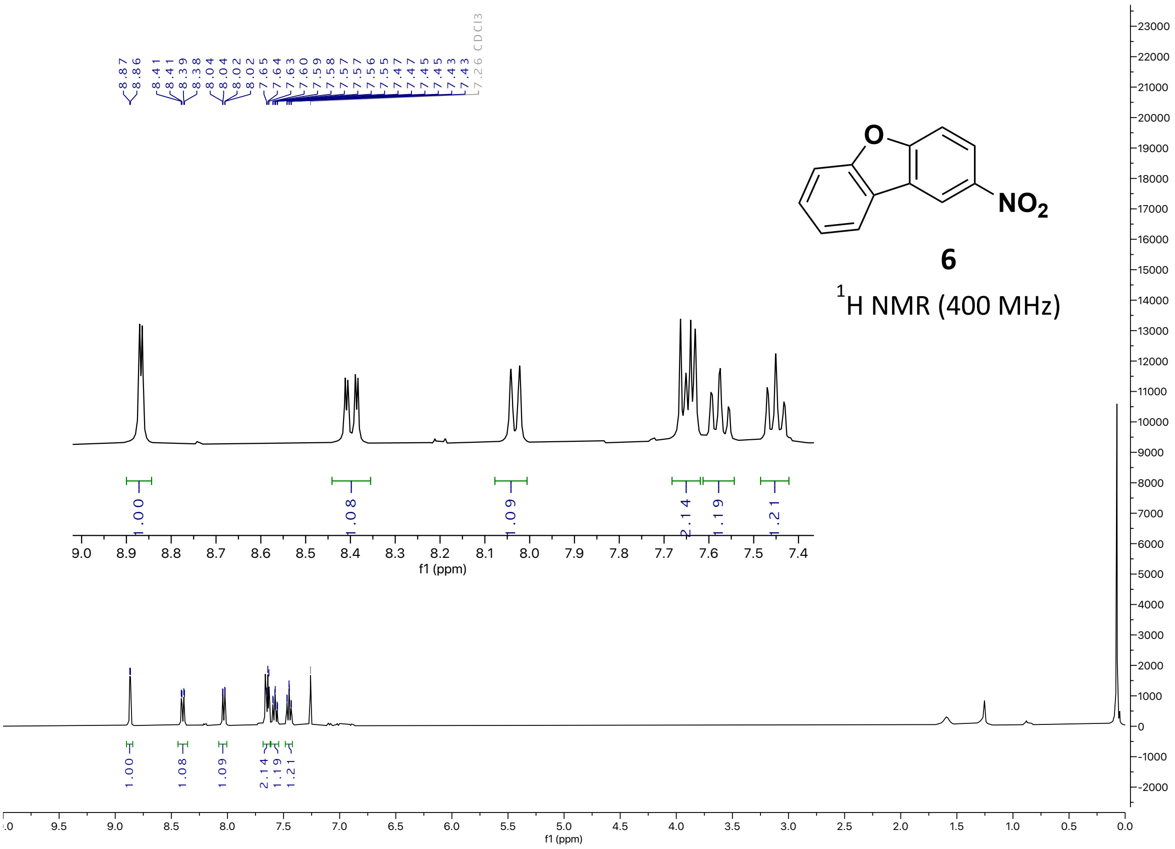
8.87
8.86
8.41
8.41
8.39
8.38
8.04
8.04
8.02
8.02
7.65
7.64
7.63
7.60
7.59
7.58
7.57
7.57
7.56
7.55
7.47
7.47
7.45
7.43
7.43
7.26 CDCl3
O
NO2
6
1H NMR (400 MHz)
1.00
1.08
1.09
2.14
1.19
1.21
f1 (ppm)

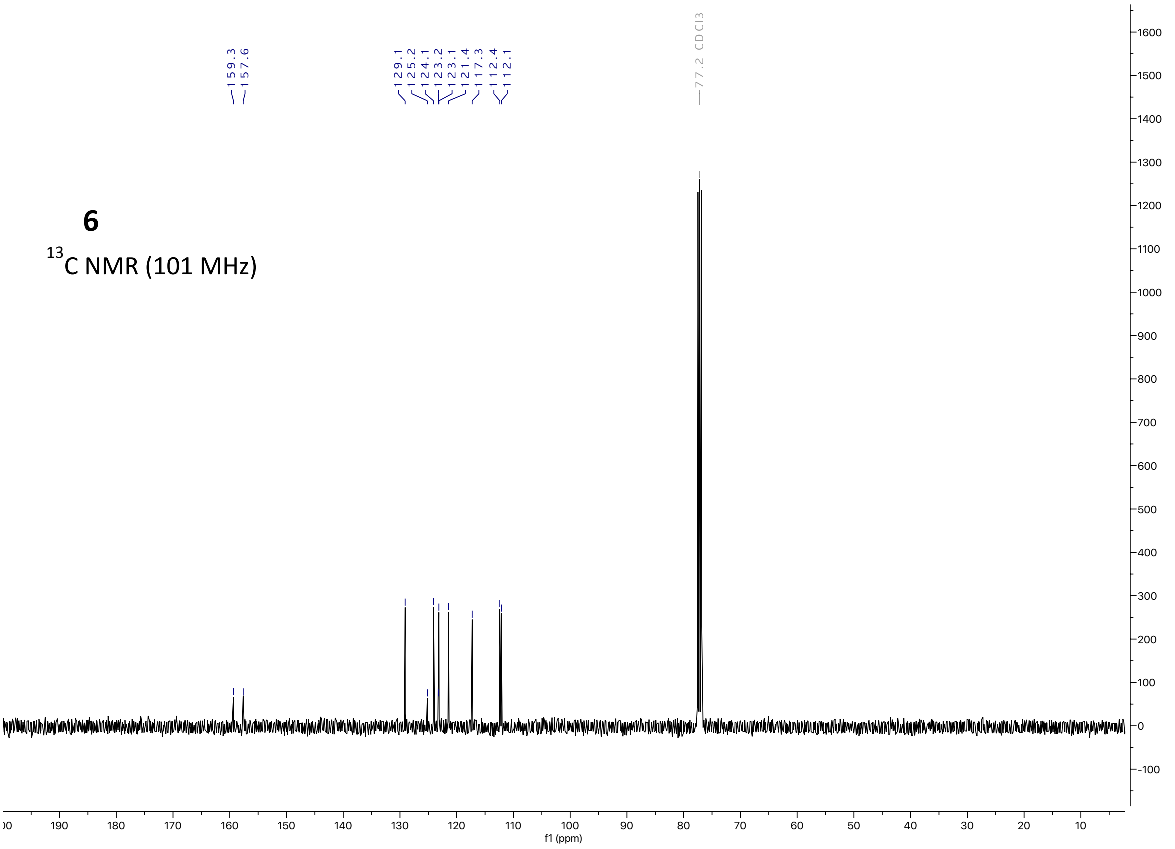
159.3
157.6
129.1
125.2
124.1
123.2
123.1
121.4
117.3
112.4
112.1
77.2 CDCl3
6
13C NMR (101 MHz)
f1 (ppm)

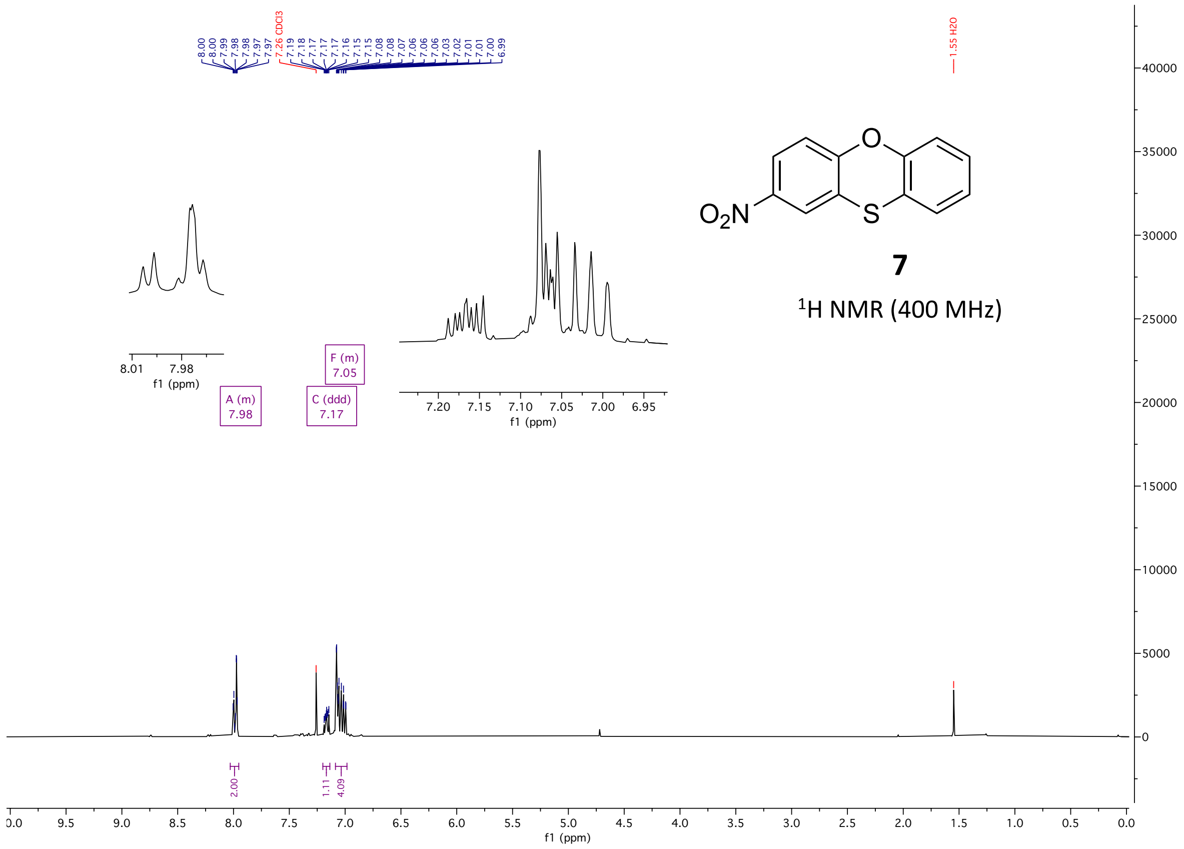
7
^{1}H NMR (400 MHz)
A (m) 7.98
C (ddd) 7.17
F (m) 7.05
7.26 CDCl3
1.55 H2O
2.00
1.11
4.09
f1 (ppm)

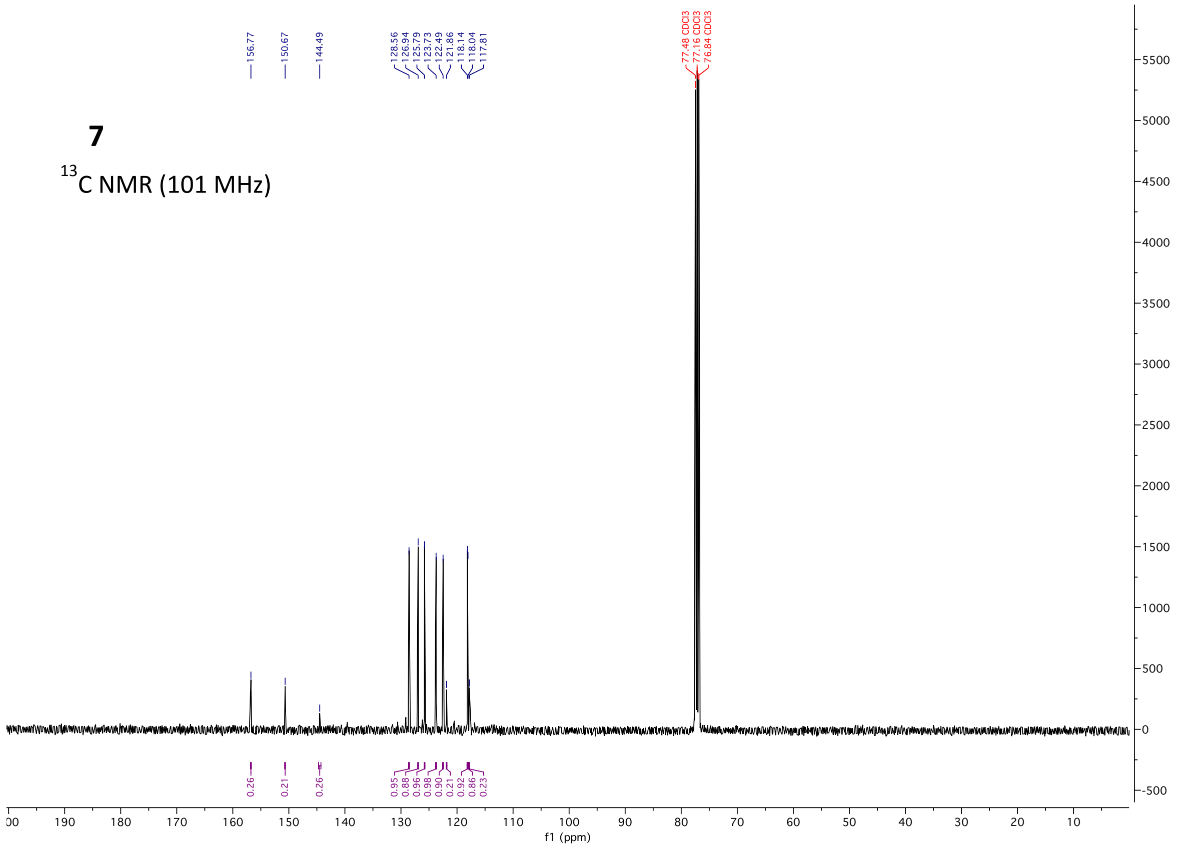
7
^{13}C NMR (101 MHz)
156.77
150.67
144.49
128.56
126.94
125.79
123.73
122.49
121.86
118.14
118.04
117.81
77.48 CDCl3
77.16 CDCl3
76.84 CDCl3
f1 (ppm)

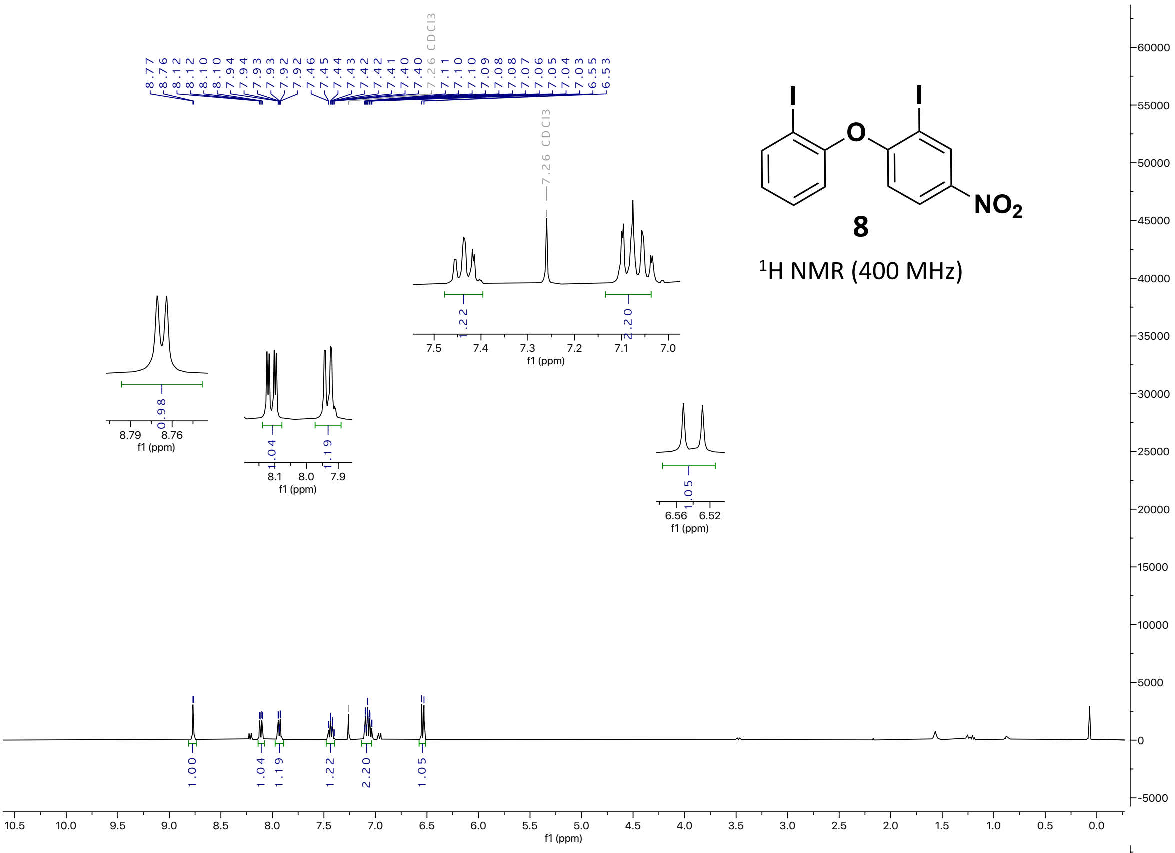
8
1H NMR (400 MHz)

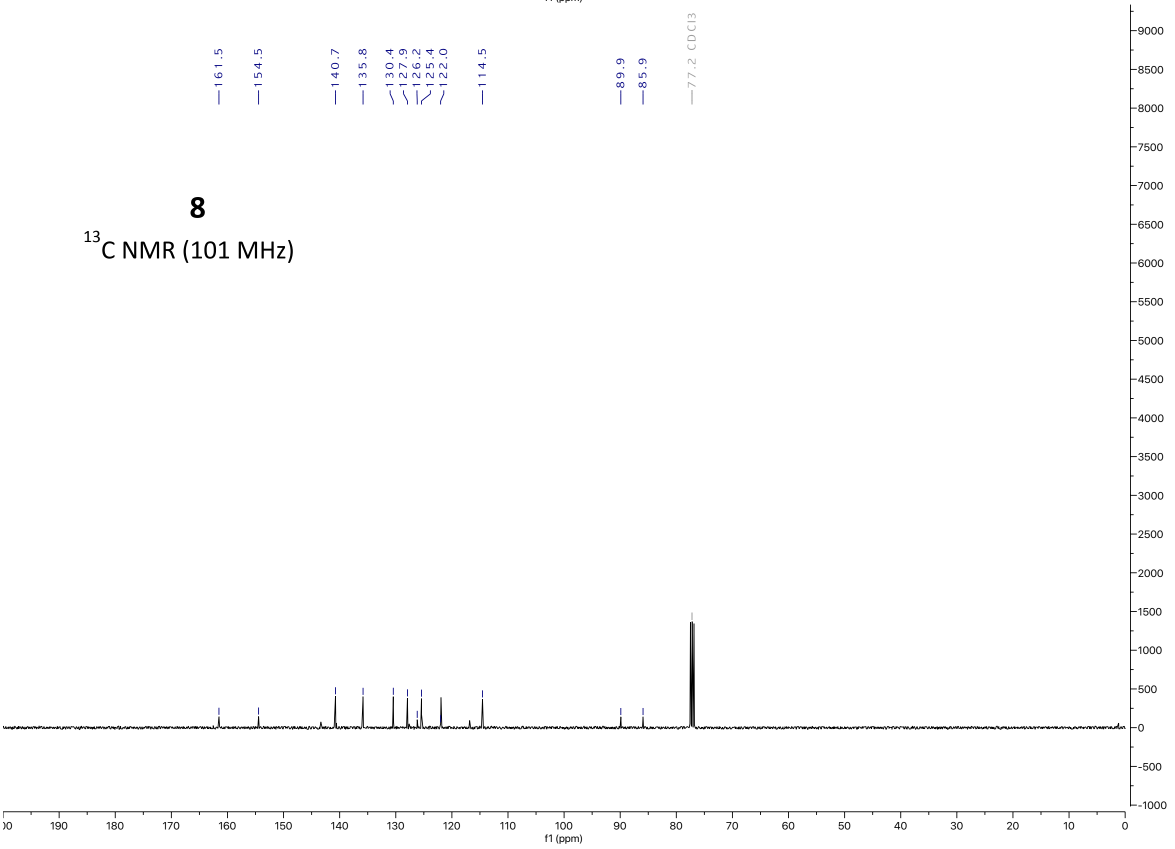
8
13C NMR (101 MHz)

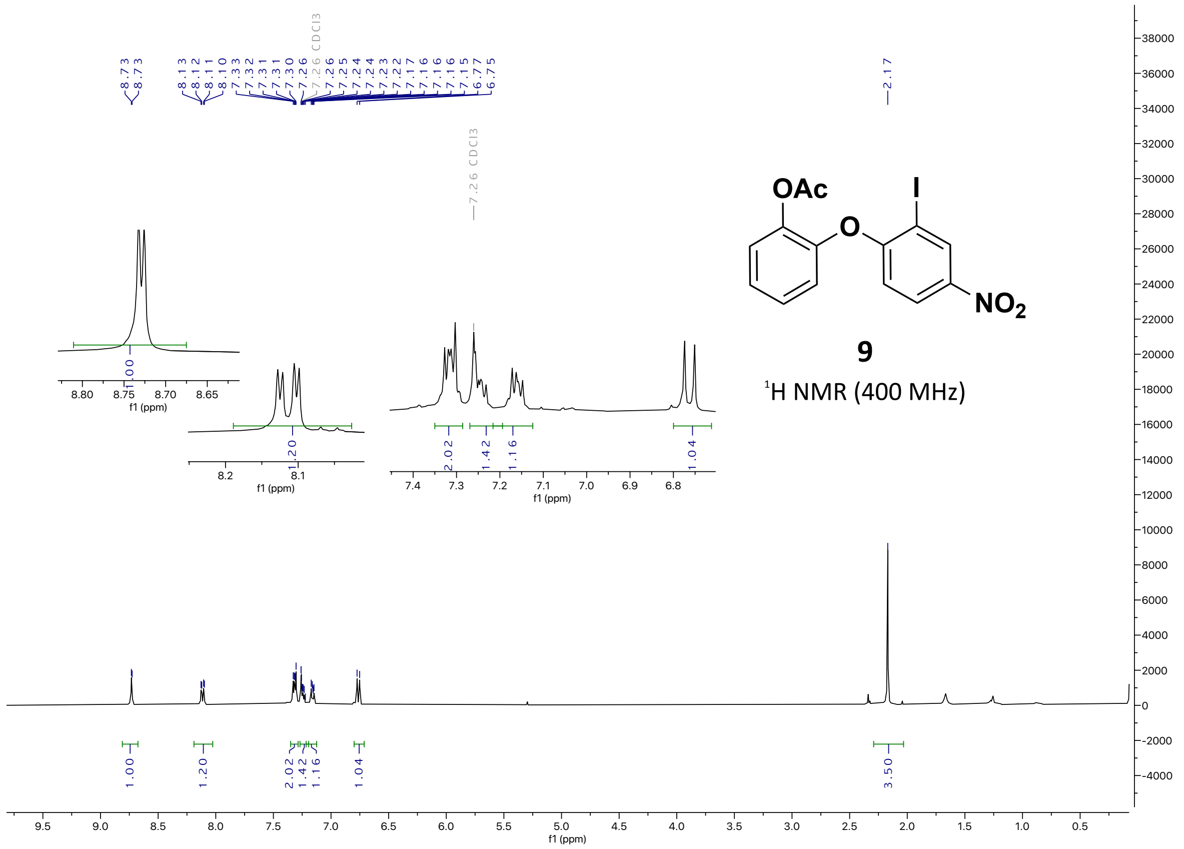
OAc
I
O
NO2
9
1H NMR (400 MHz)
8.73
8.73
8.13
8.12
8.11
8.10
7.33
7.32
7.31
7.31
7.30
7.26
7.26 CDCl3
7.26
7.25
7.24
7.24
7.23
7.22
7.17
7.16
7.16
7.16
7.15
6.77
6.75
2.17
1.00
1.20
2.02
1.42
1.16
1.04
3.50
f1 (ppm)

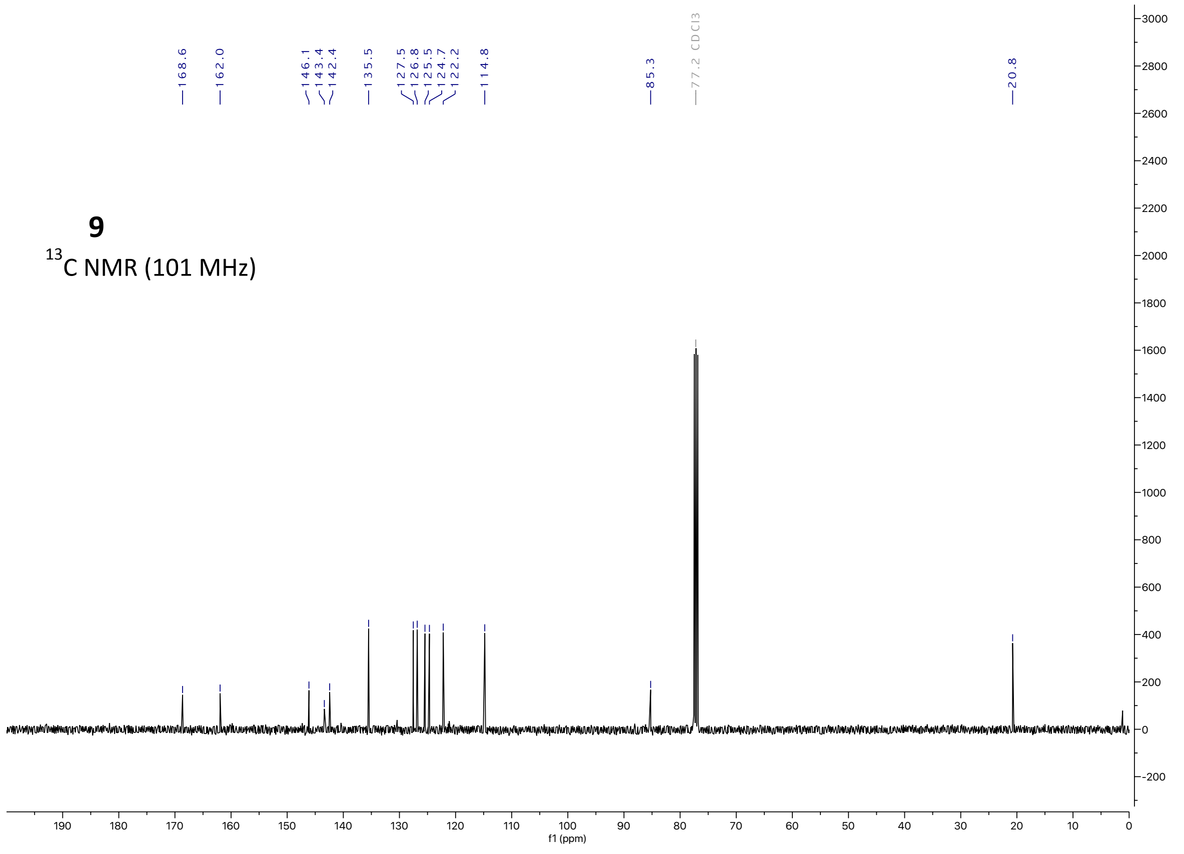
9
13C NMR (101 MHz)
168.6
162.0
146.1
143.4
142.4
135.5
127.5
126.8
125.5
124.7
122.2
114.8
85.3
77.2 CDCl3
20.8
f1 (ppm)

9

HMBC 2D NMR

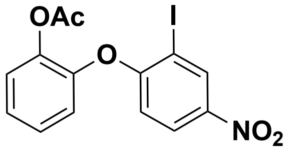

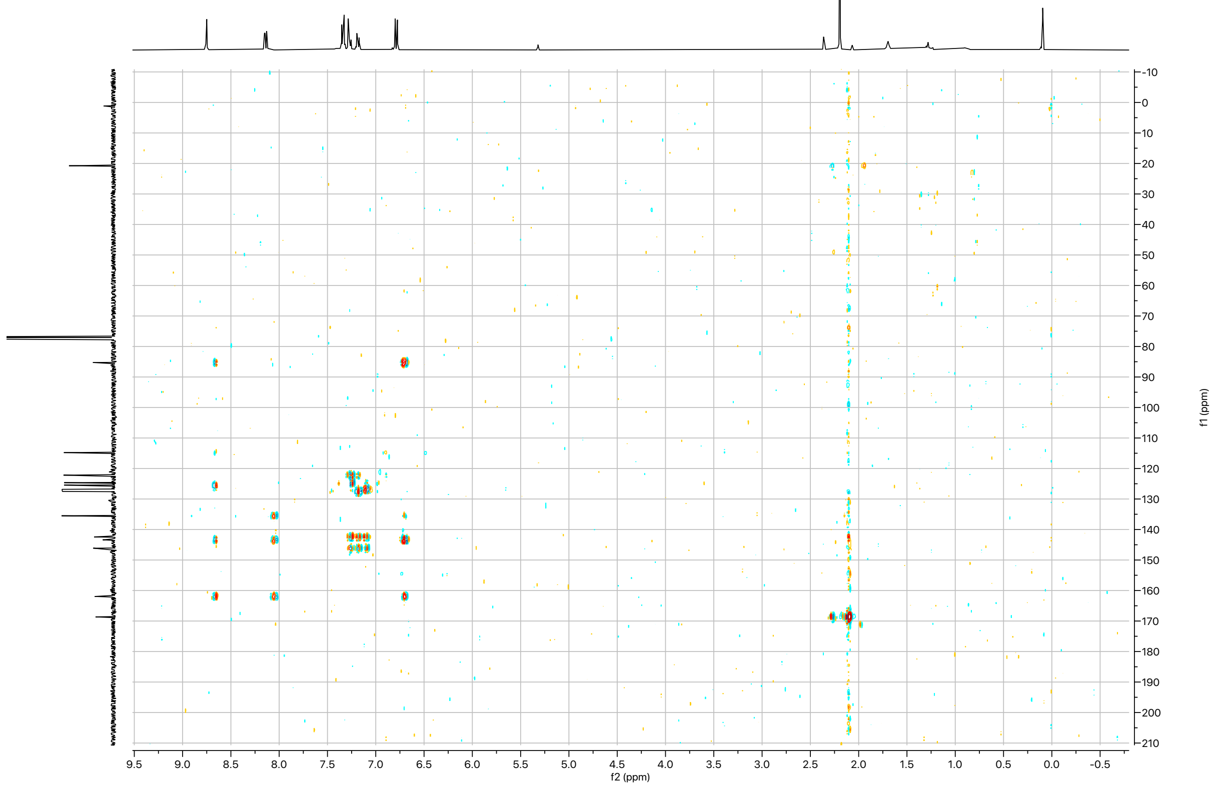

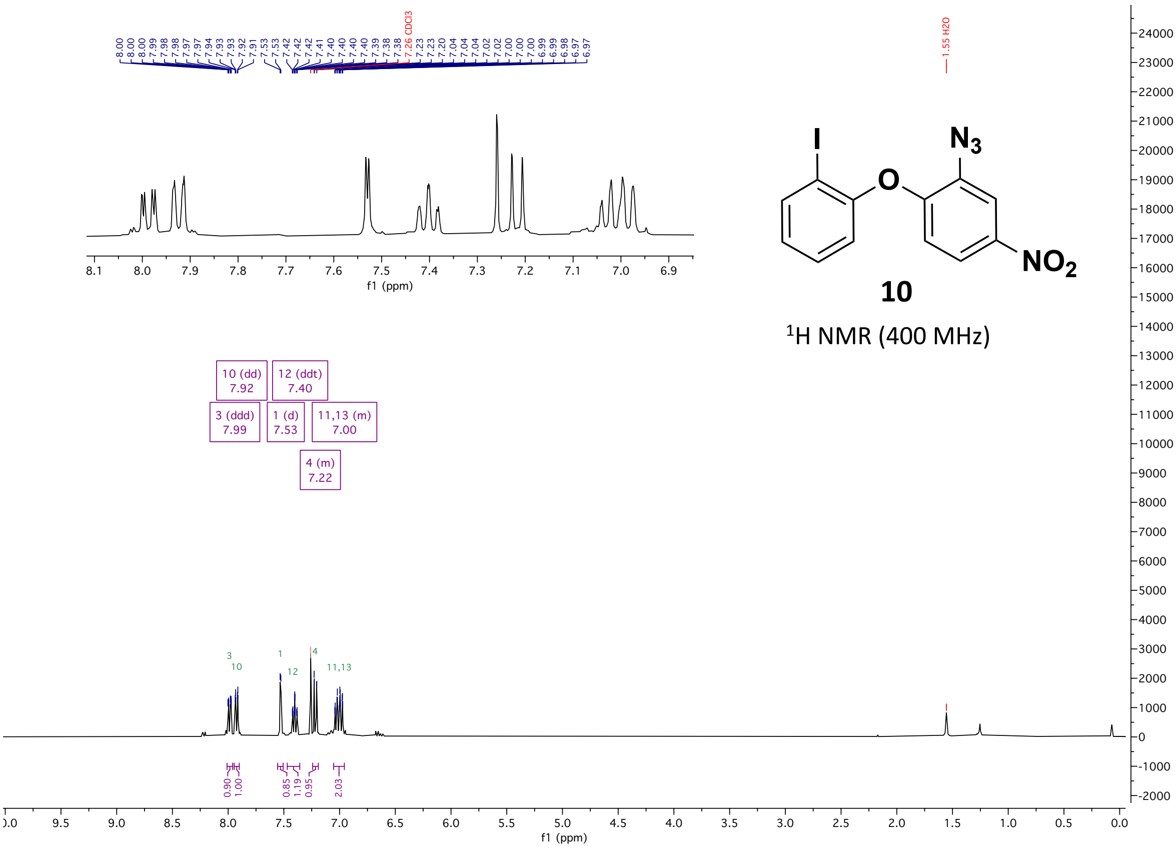

10

^1H NMR (400 MHz)

10

^{13}C NMR (101 MHz)

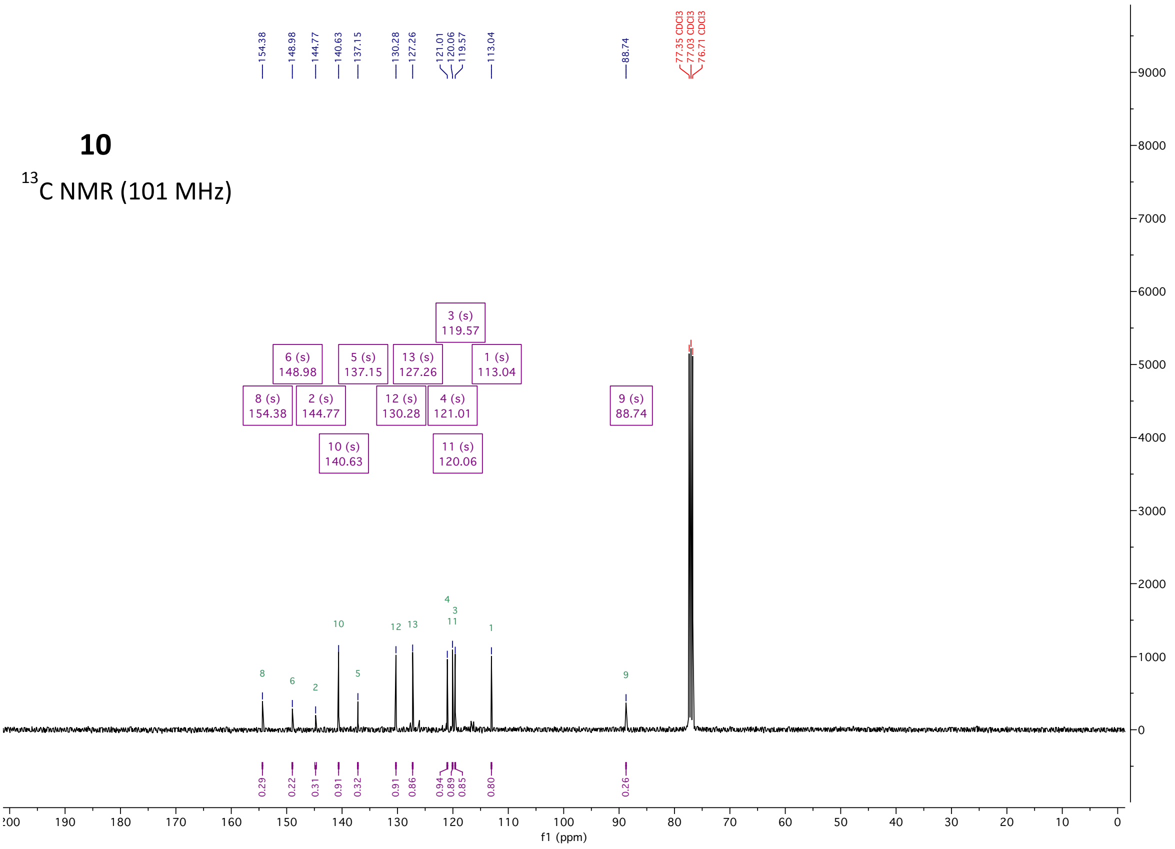

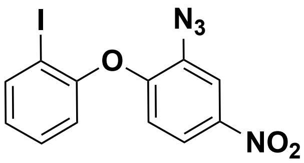

10
HMBC 2D NMR

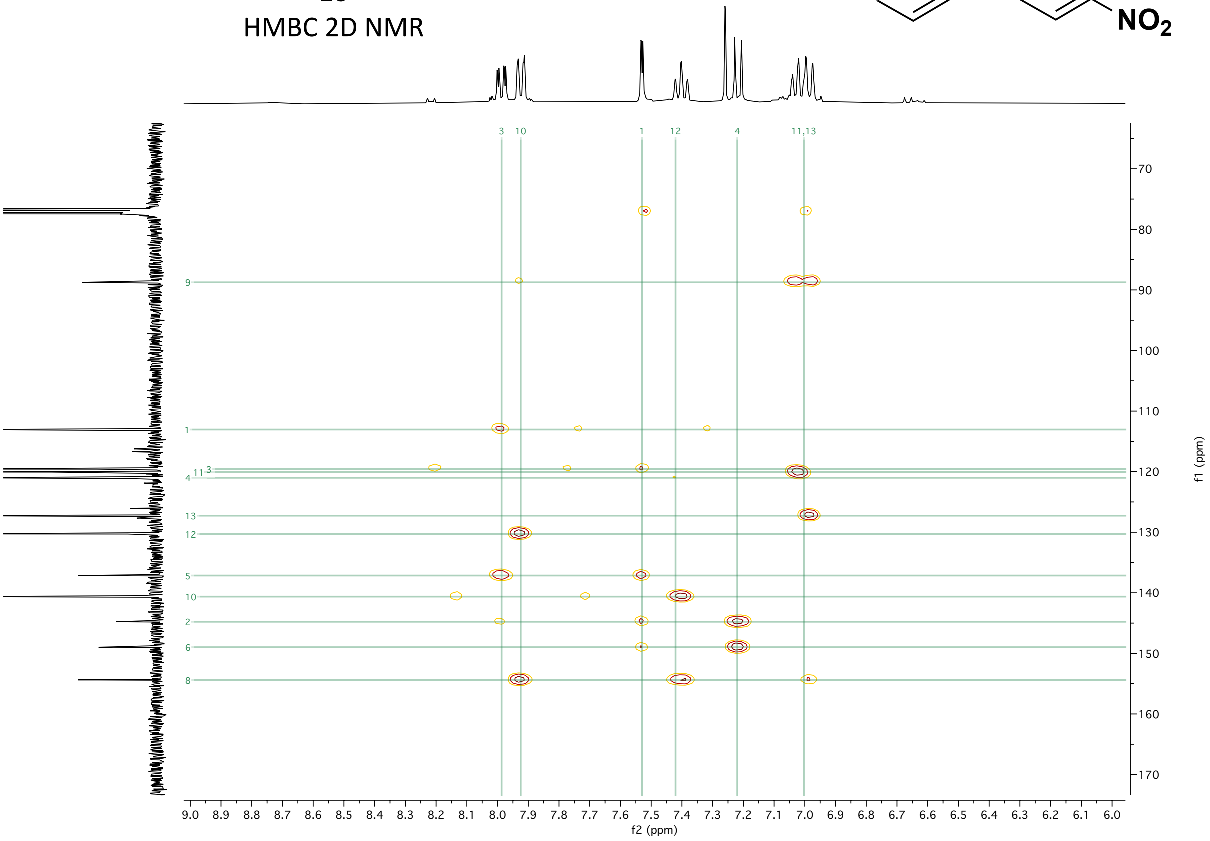

10
COSY 2D NMR

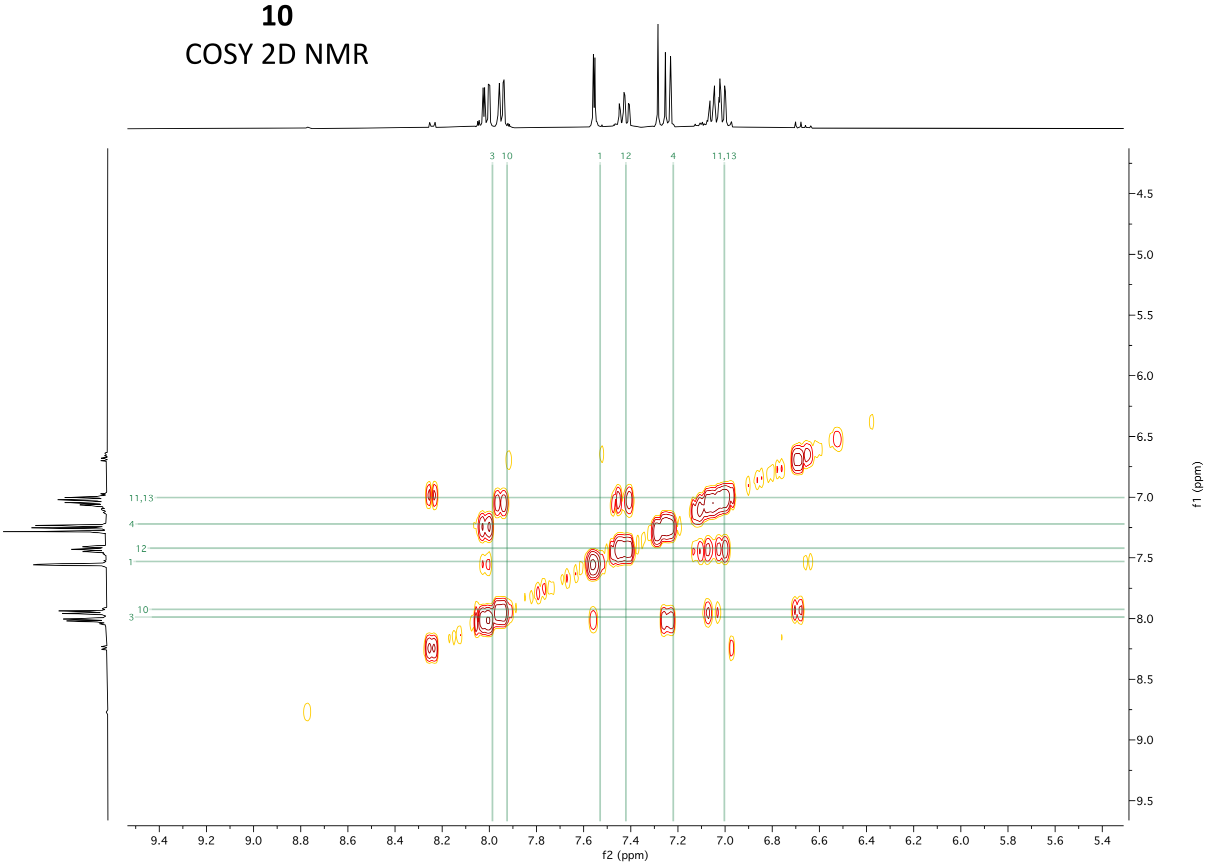

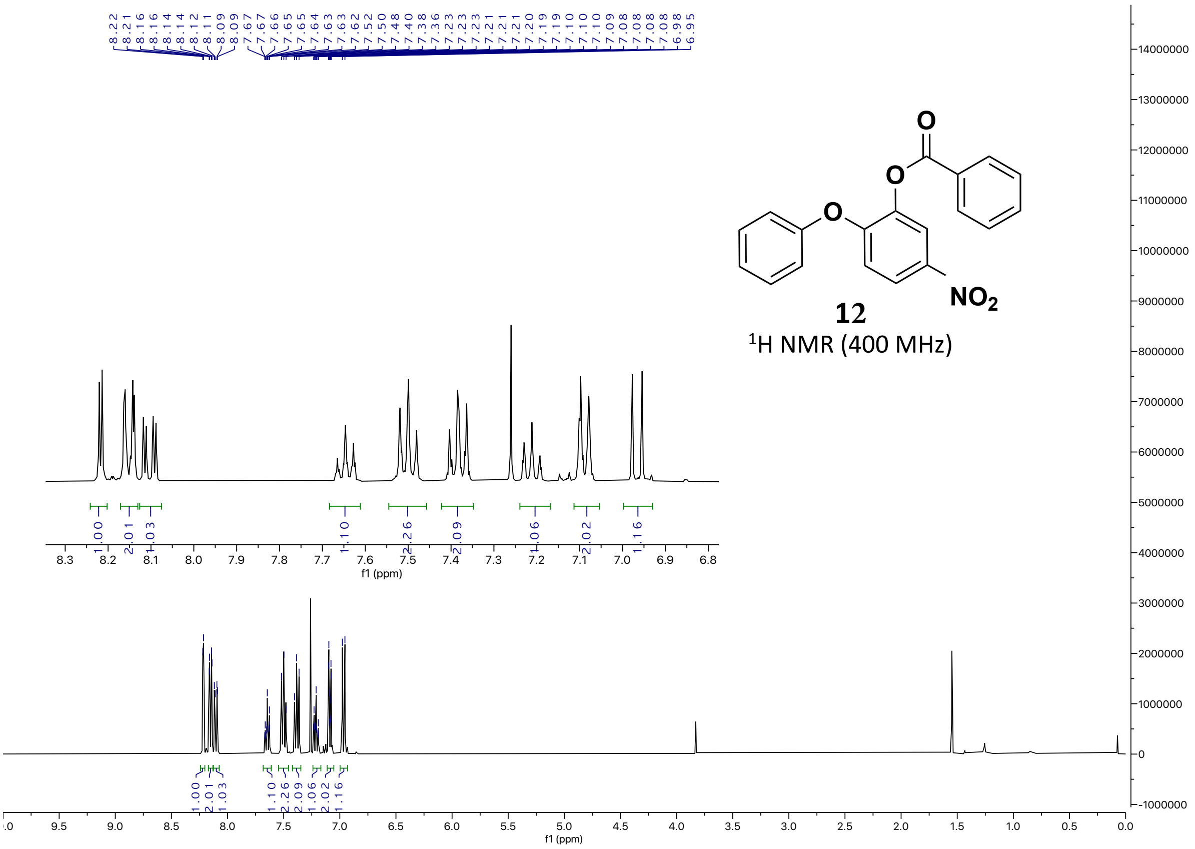

12

^{1}H NMR (400 MHz)

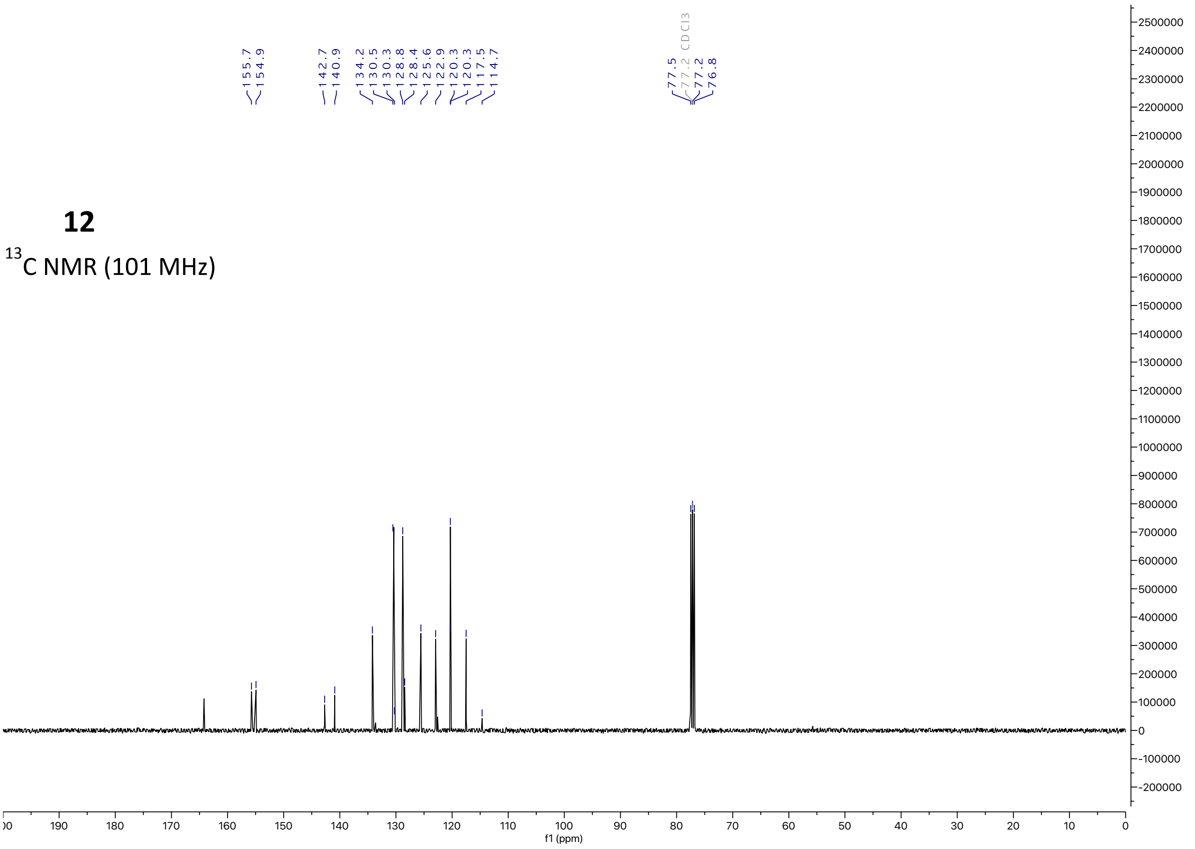

12

^{13}C NMR (101 MHz)

13

^{1}H NMR (400 MHz)

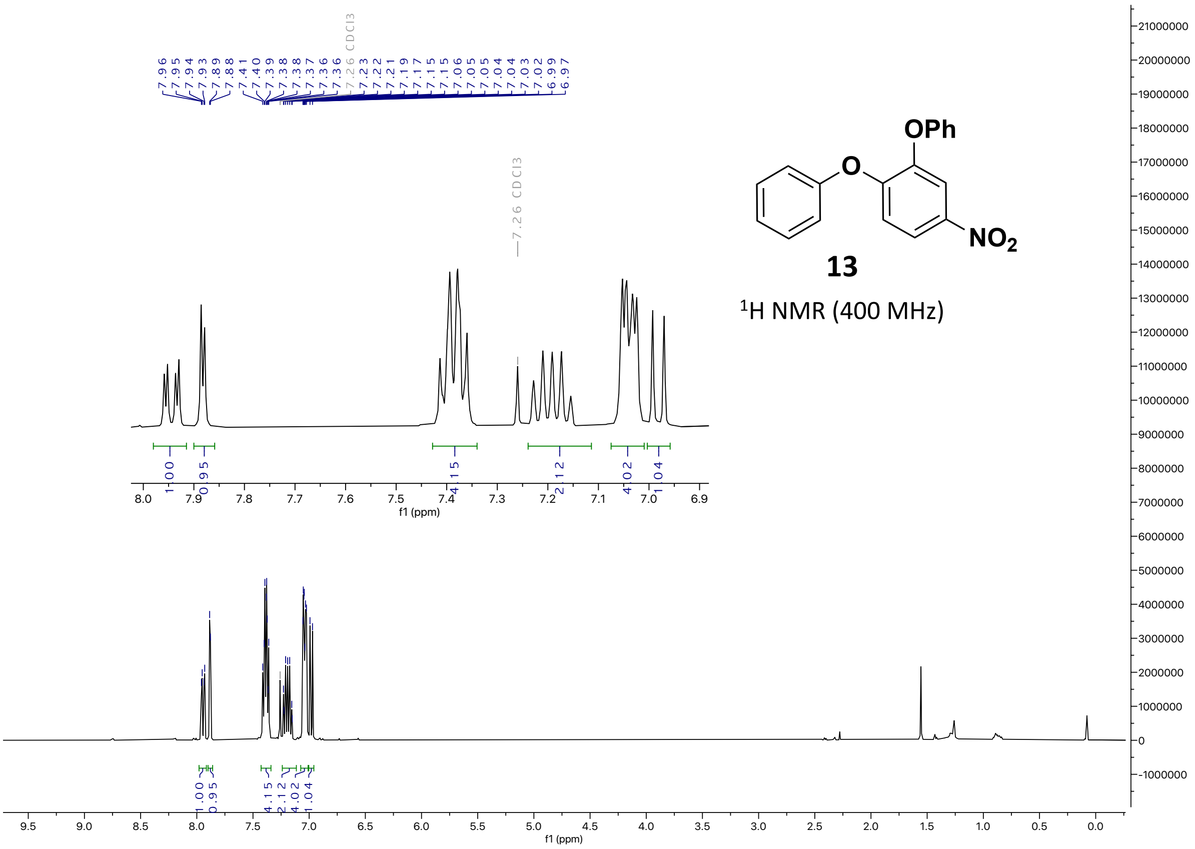

13

^{13}C NMR (101 MHz)

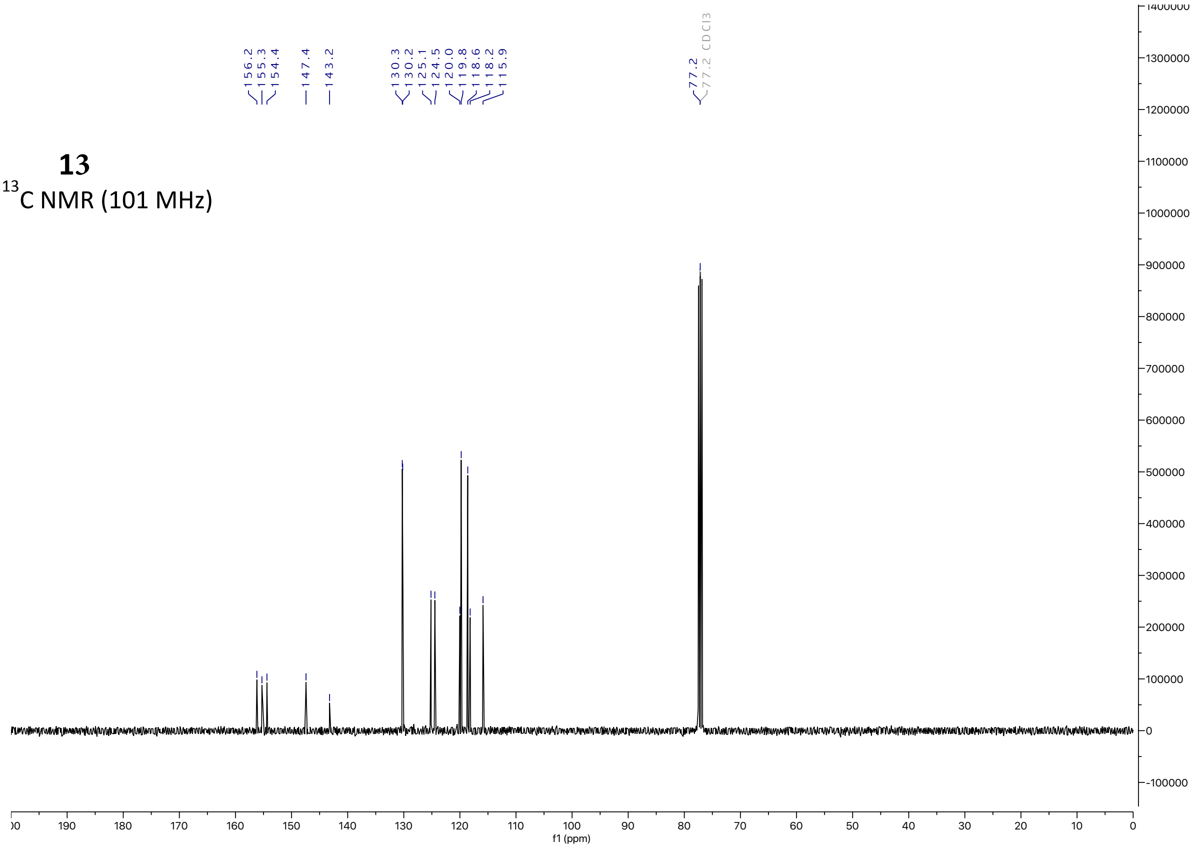

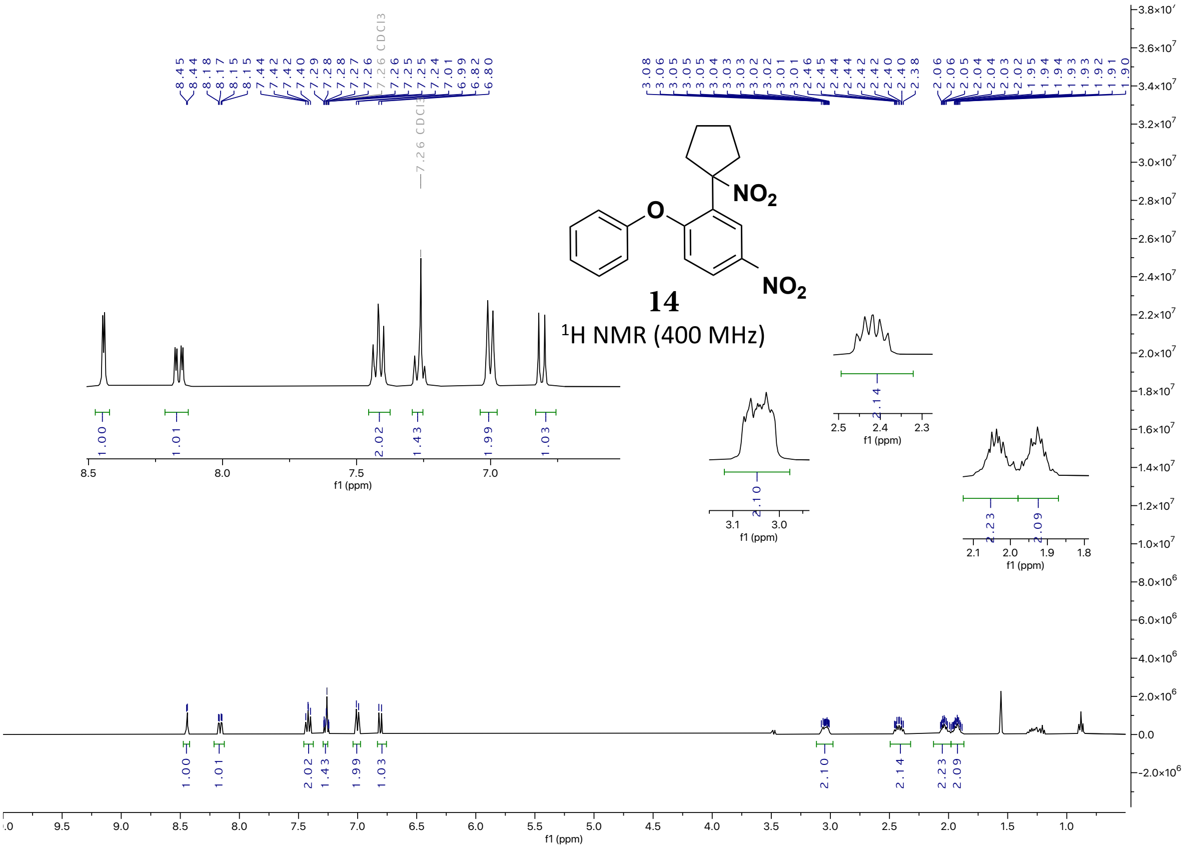
14
^{1}H NMR (400 MHz)
8.45 8.44 8.18 8.17 8.15 8.15 7.44 7.42 7.40 7.29 7.28 7.28 7.27 7.26 7.26 7.25 7.25 7.24 7.01 6.99 6.82 6.80
7.26 CDCl3
3.08 3.06 3.05 3.05 3.05 3.04 3.03 3.03 3.02 3.02 3.01 3.01 2.46 2.45 2.44 2.42 2.42 2.40 2.40 2.38 2.06 2.06 2.05 2.04 2.04 2.03 2.02 1.95 1.94 1.94 1.93 1.93 1.92 1.91 1.90
1.00 1.01 2.02 1.43 1.99 1.03 2.10 2.14 2.23 2.09
f1 (ppm)

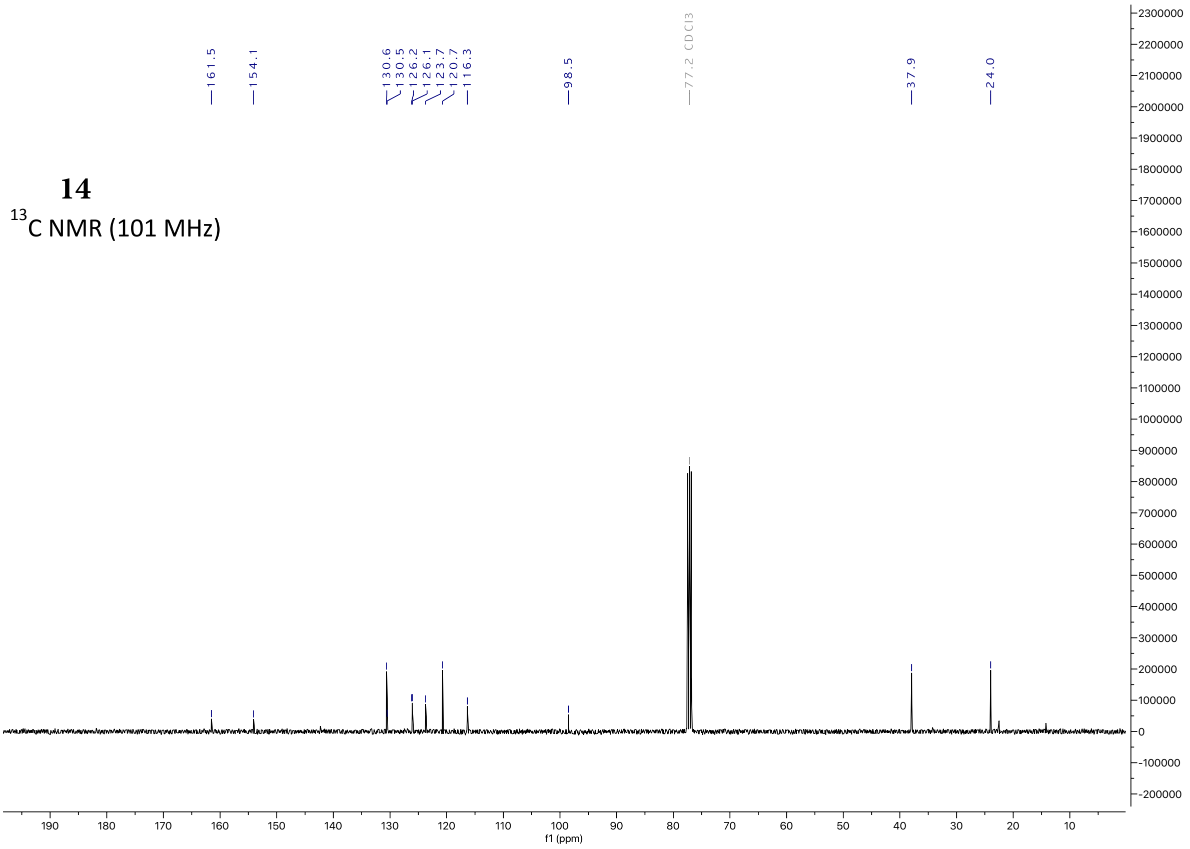
14
^{13}C NMR (101 MHz)
161.5 154.1 130.6 130.5 126.2 126.1 123.7 120.7 116.3 98.5 77.2 CDCl3 37.9 24.0
f1 (ppm)

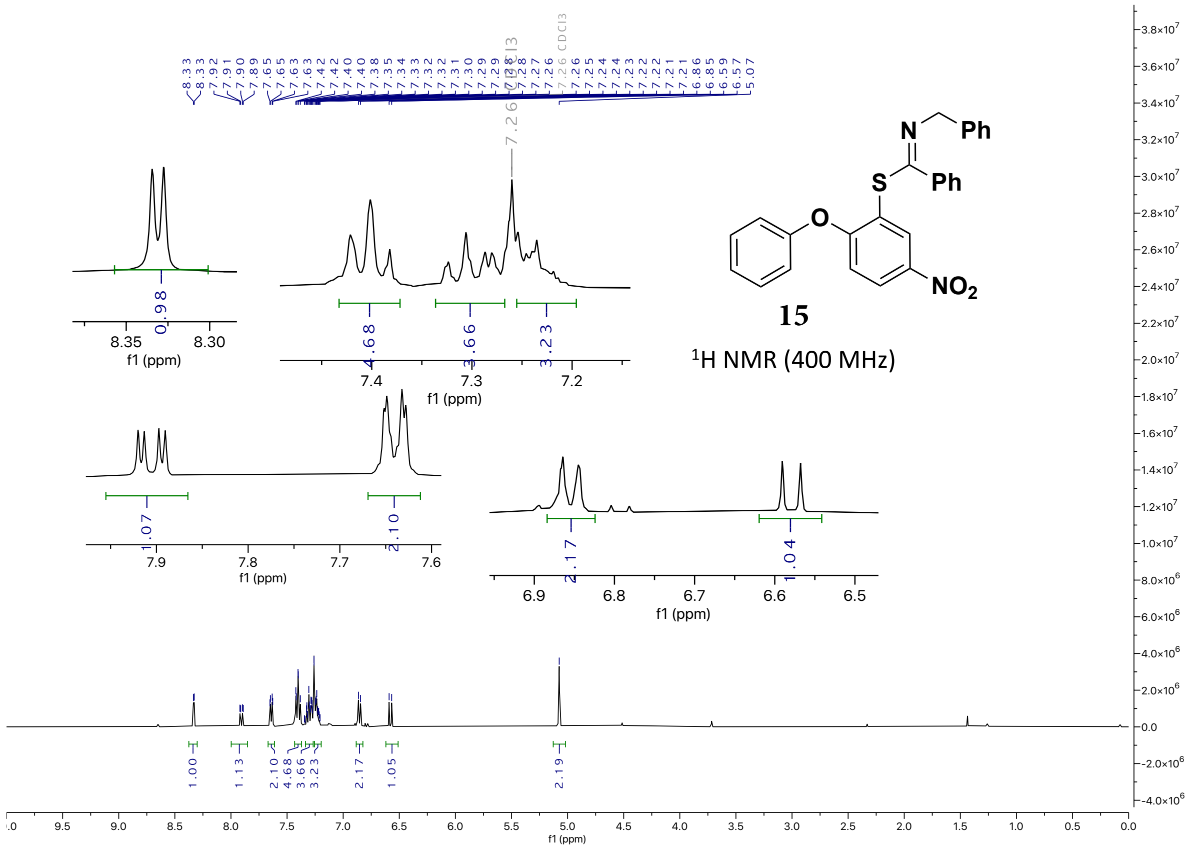

15

^{1}H NMR (400 MHz)

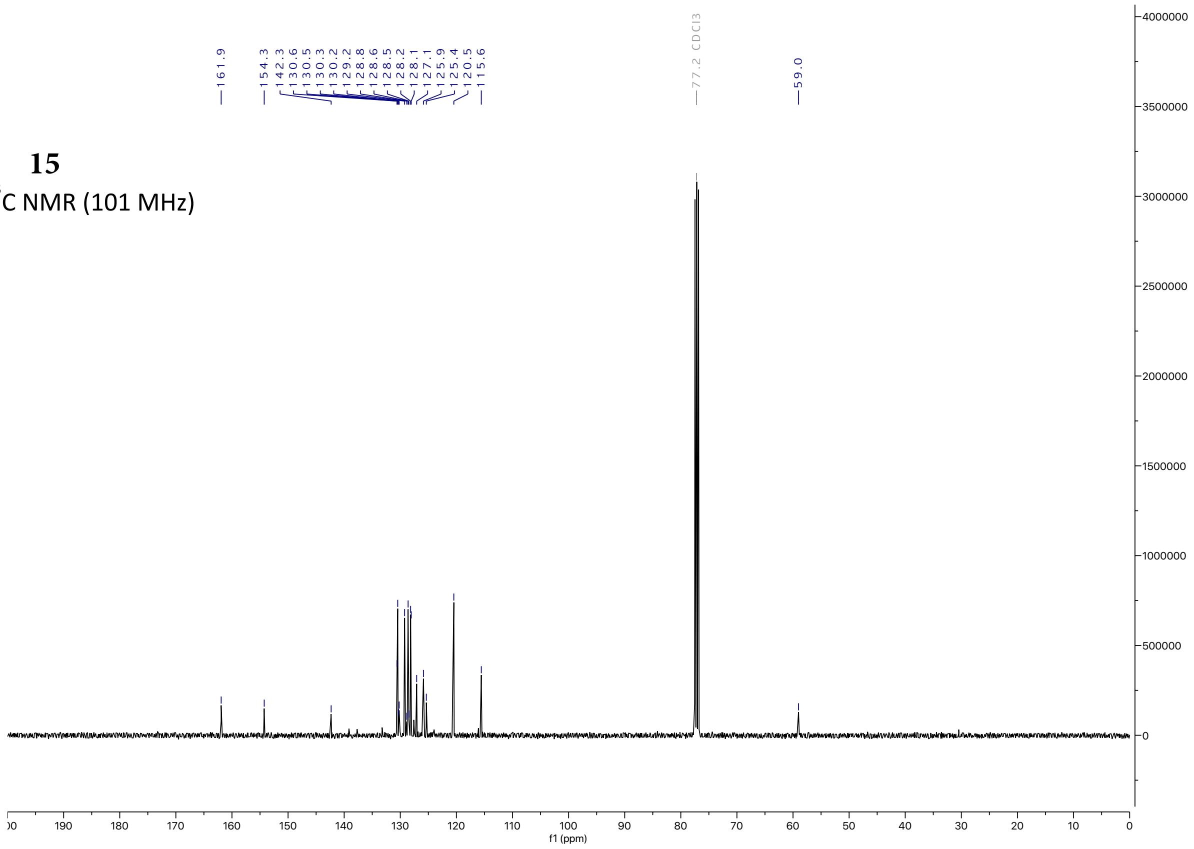

15

^{13}C NMR (101 MHz)

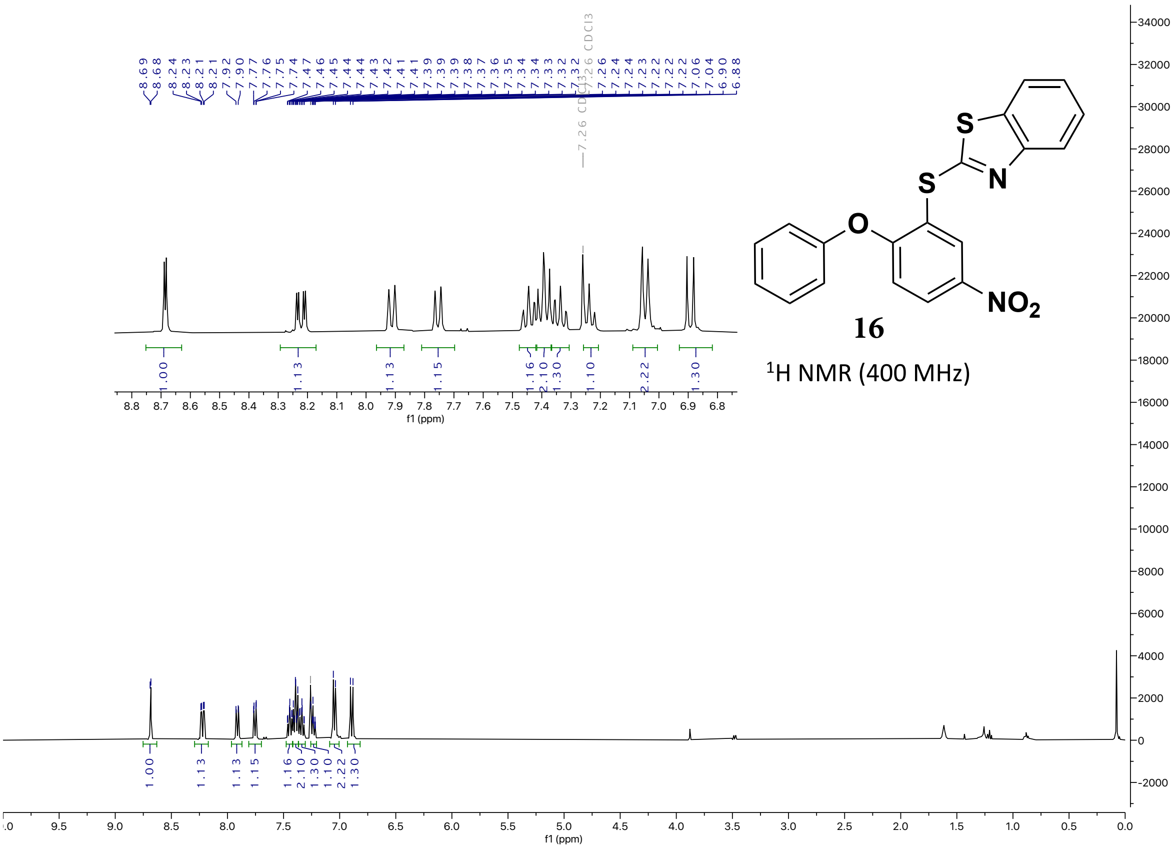
16
1H NMR (400 MHz)
NO2
f1 (ppm)

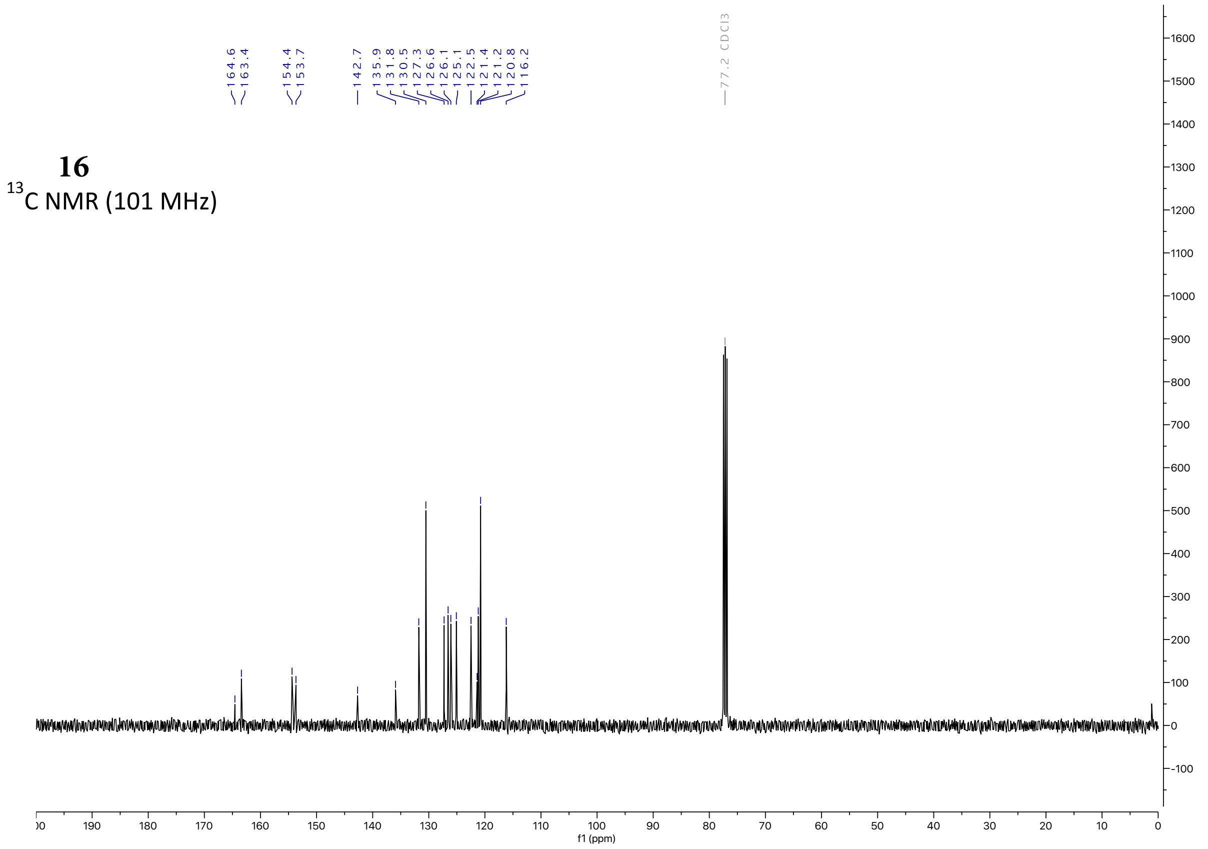
16
13C NMR (101 MHz)
f1 (ppm)

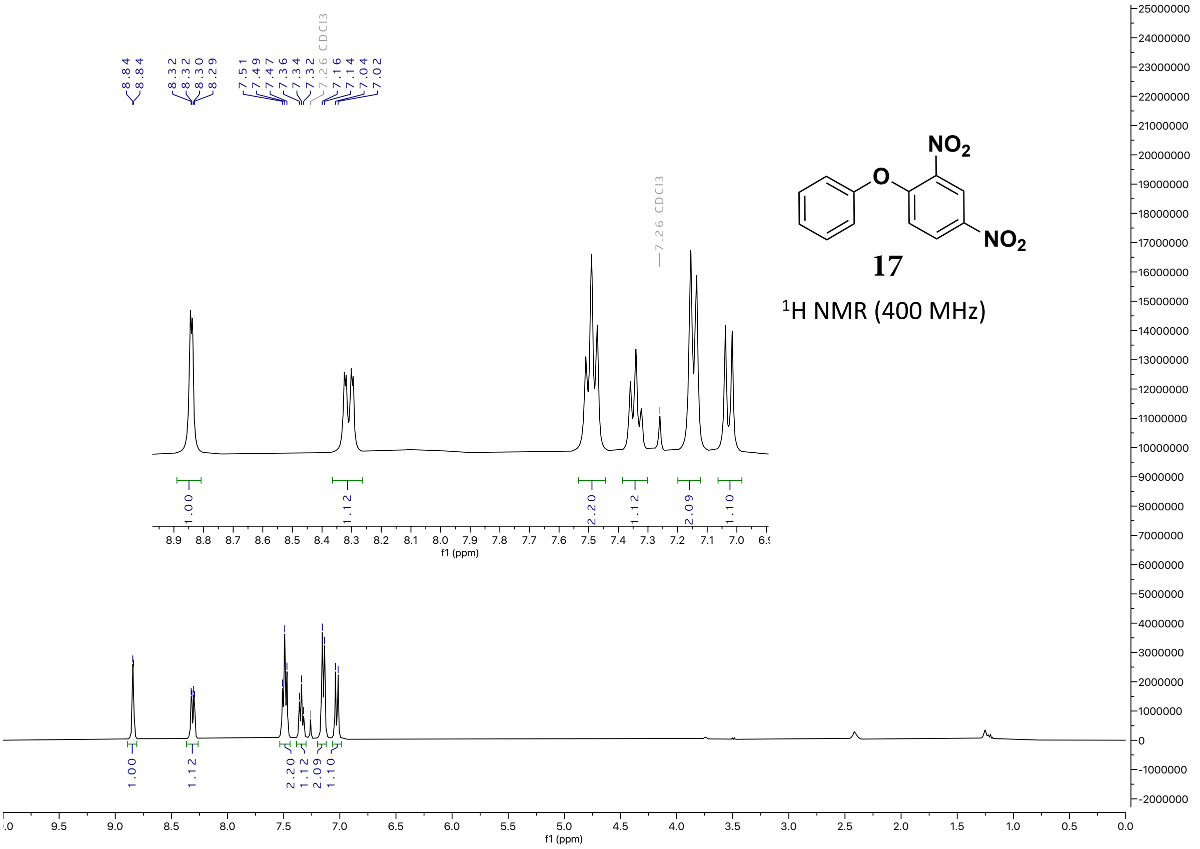

8.84
8.84
8.32
8.32
8.30
8.29
7.51
7.49
7.47
7.36
7.34
7.32
7.26 CDCl3
7.16
7.14
7.04
7.02
NO2
O
NO2
17
1H NMR (400 MHz)
1.00
1.12
2.20
1.12
2.09
1.10
f1 (ppm)

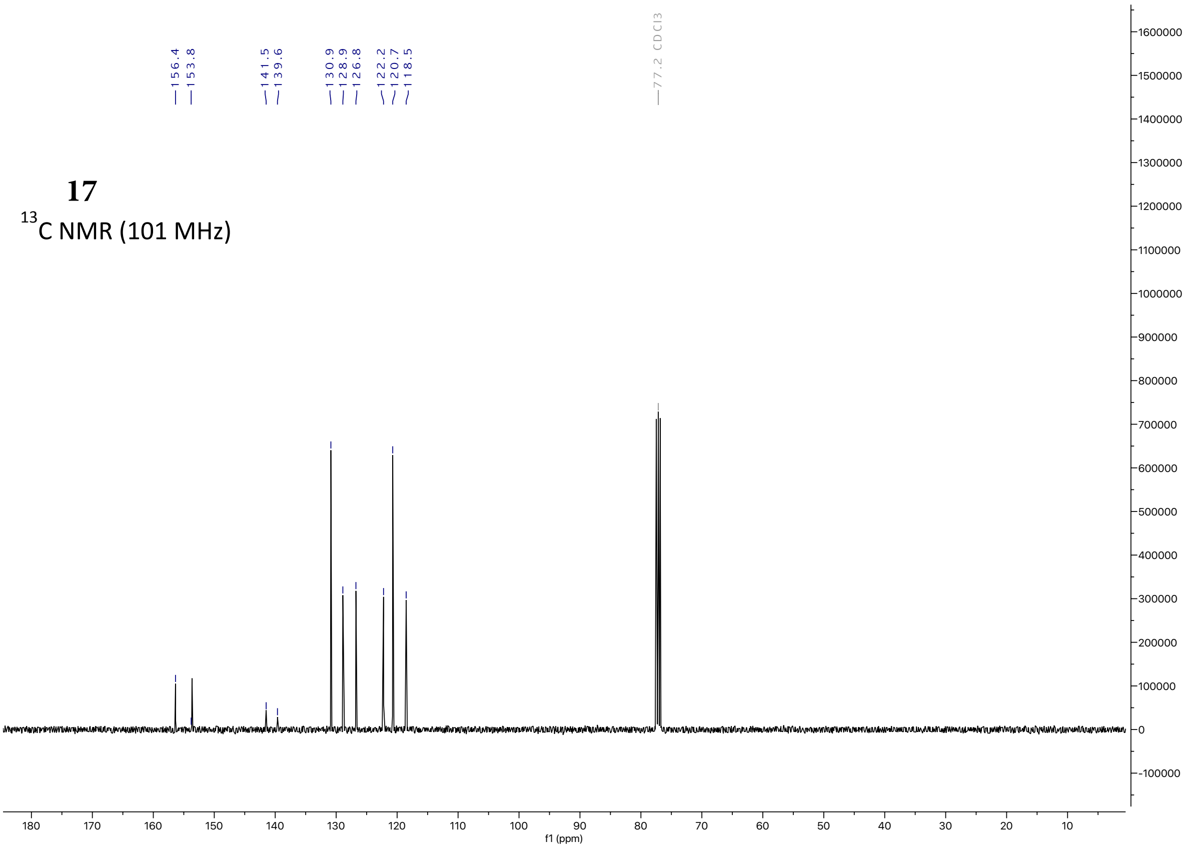

156.4
153.8
141.5
139.6
130.9
128.9
126.8
122.2
120.7
118.5
77.2 CDCl3
17
13C NMR (101 MHz)
f1 (ppm)
